# Four new Cyclohexenone with antibacterial activity from the coral-derived fungus *Aspergillus flavus*

**DOI:** 10.1007/s13659-026-00618-y

**Published:** 2026-05-12

**Authors:** Cili Wang, Jiarui Zhang, Lei Li, Sen Wang, Kai Li, Hu Hou, Pinglin Li

**Affiliations:** 1 Key Laboratory of Marine Drugs, School of Medicine and Pharmacy, Chinese Ministry of Education, Ocean University of China, Qingdao, 266003 China; 2 Key Laboratory of Marine Food Processing & Safety Control, College of Food Science and Engineering, Ocean University of China, Qingdao, 266003 China; 3 Laboratory for Marine Drugs and Bioproducts, Qingdao Marine Science and Technology Center, Qingdao, 266237 China

**Keywords:** *Aspergillus flavus*, Cyclohexenones, Aspergiflones A–D, Structure elucidation, Antibacterial activity

## Abstract

Marine fungus-derived natural products are an important source of new drugs and lead compounds. In this study, guided by the Global Natural Products Social (GNPS) molecular networking strategy, four new cyclohexenone derivatives, aspergiflones A–D (**1**–**4**), together with three known analogues (**5**–**7**), were isolated from the coral-derived fungus *Aspergillus flavus.* The structures of these compounds were elucidated through extensive spectroscopic analyses, comparison with literature data, hydrolysis reactions, quantum chemical calculations, and TDDFT-ECD calculations. Structurally, aspergiflone A (**1**) is a novel cyclohexenone dimer featuring an unprecedented skeleton in which two cyclohexenone units are connected at C-15 via a C−C single bond. Compound **1** exhibited potent antibacterial activity against *Escherichia coli*. Mechanism studies revealed that compound **1** disrupts bacterial cell morphology, increases cell membrane permeability, and decreases intracellular K^+^ ion levels. Additionally, transcriptome analysis revealed that the differentially expressed genes (DEGs) were mainly associated with cellular process regulation, stimulus response, catalytic, and binding functions. Several pathways were also significantly affected in *Escherichia coli*, including ABC transporters, cofactor biosynthesis, energy metabolism, and arginine biosynthesis. The finding indicated that aspergiflone A has considerable potential in food safety.

**Graphical abstract:**

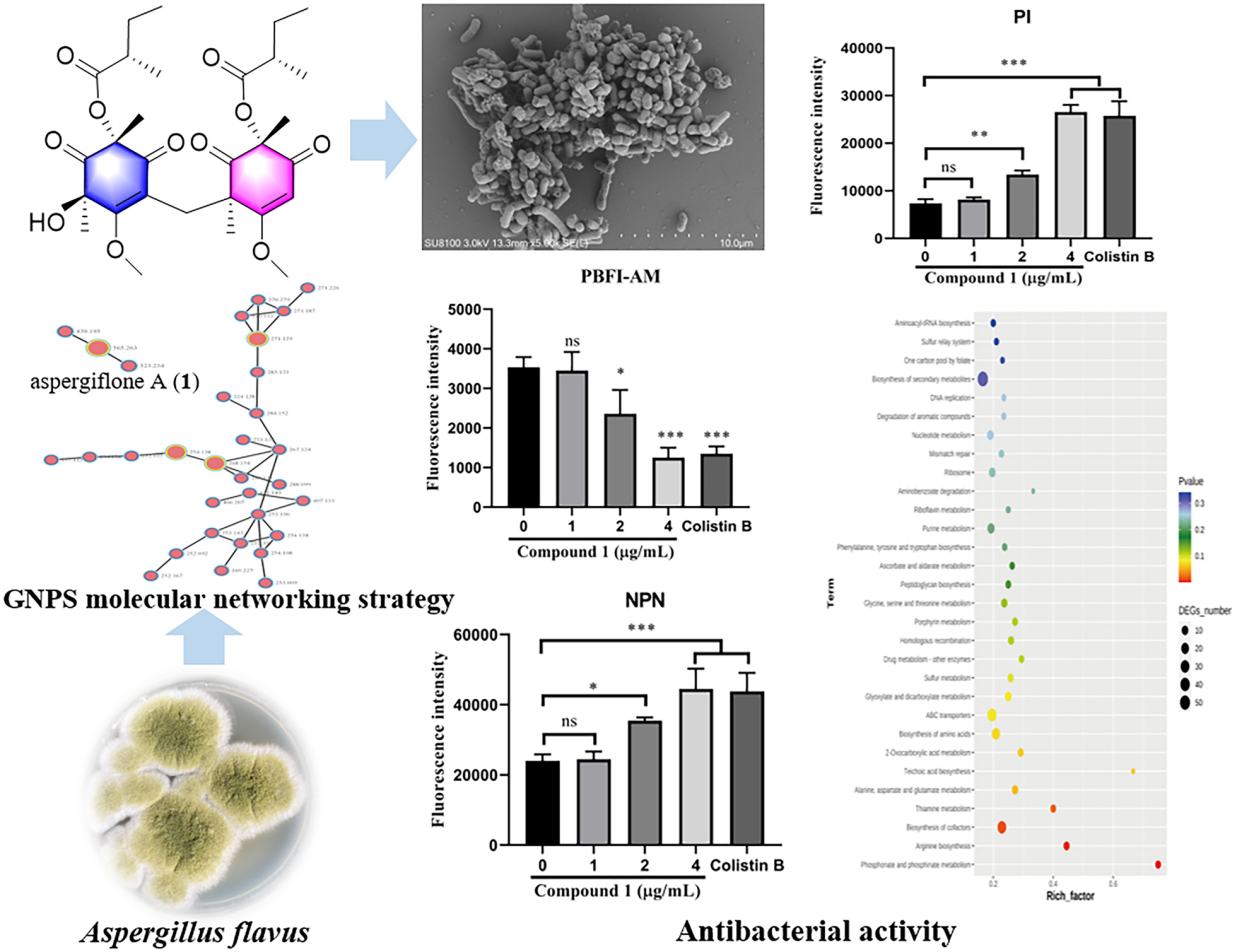

**Supplementary Information:**

The online version contains supplementary material available at 10.1007/s13659-026-00618-y.

## Introduction

The genus *Aspergillus* (Class: Eurotiomycetes, Family: Trichocomaceae) is an ancient fungal group first cataloged in 1729. To date, over 400 species of genus *Aspergillus* have been identified [1–4]. These fungi serve as invaluable resources in medicine, agriculture, and industry [5], contributing to the development of products such as the cholesterol-lowering drug lovastatin [6], the antifungal agent echinocandin B [7] and the industrial additive citric acid [8]. In recent years, numerous chemically diverse secondary metabolites have been discovered from this genus, including stephacidin A [9], wheldone [10], aspersteroids A − C [11], bisaspochalasin A − C [12], many of which exhibit significant bioactivities such as cytotoxicity, immunosuppressive and antimicrobial properties.

Cyclohexenones, typically characterized by a six-membered carbon ring containing one or two carbonyl groups and double bonds, represent a class of structures primarily isolated from genus *Aspergillus* [13–18], *Phoma* [19–22], *Paecilomyces* [23], *Altenaria* [24], *Diaporthe* [25], and *Cordia* [26], and *Phyllidiella* [27]. Since the first cyclohexenone was isolated from a blackleg fungus *Phoma lingam* [22], more than one hundred cyclohexenone derivatives have been isolated and characterized [13–27]. These compounds exhibit diverse biological activities, including cytotoxicity [15], antibacterial activity [16], and tyrosinase inhibitory activity [24], and have attracted growing research interest.

The development of a more efficient strategy is essential for natural product drug discovery. Molecular networking has been applied as a promising method for efficiently clustering and visualizing the small molecules with similar fragmentation patterns [28]. This clustering approach can identify the potentially known compounds and related analogs, facilitating dereplication [29]. Considering that the fungi of the genus *Aspergillus* has been recognized as a rich source of cyclohexenones [13–18], a coral-derived fungus, *Aspergillus flavus* was selected and analyzed using the LC − MS/MS molecular networking. Subsequently, LC−MS/MS-guided isolation led to four new cyclohexenone derivatives (**1**–**4**), together with three known analogues co-isolated, 1-amino-2,6-dimethyl-6-hydroxy-4-(2'-methyl-1-oxobutyl)−3-methoxy-2,4-cyclohexadien-1-one (**5**) [19], acetylphomaligol A (**6**) [23], and phomaligol A (**7**) [23]. Structurally, compound **1** is a novel cyclohexenone dimer featuring an unprecedented skeleton [30]. The isolated compounds were evaluated for in vitro antibacterial activity against four common foodborne pathogens. Herein, the isolation, structure elucidation, proposed biosynthetic pathway, and biological evaluation of compounds **1**–**4** are reported.

## Results and discussion

**Fig. 1:**
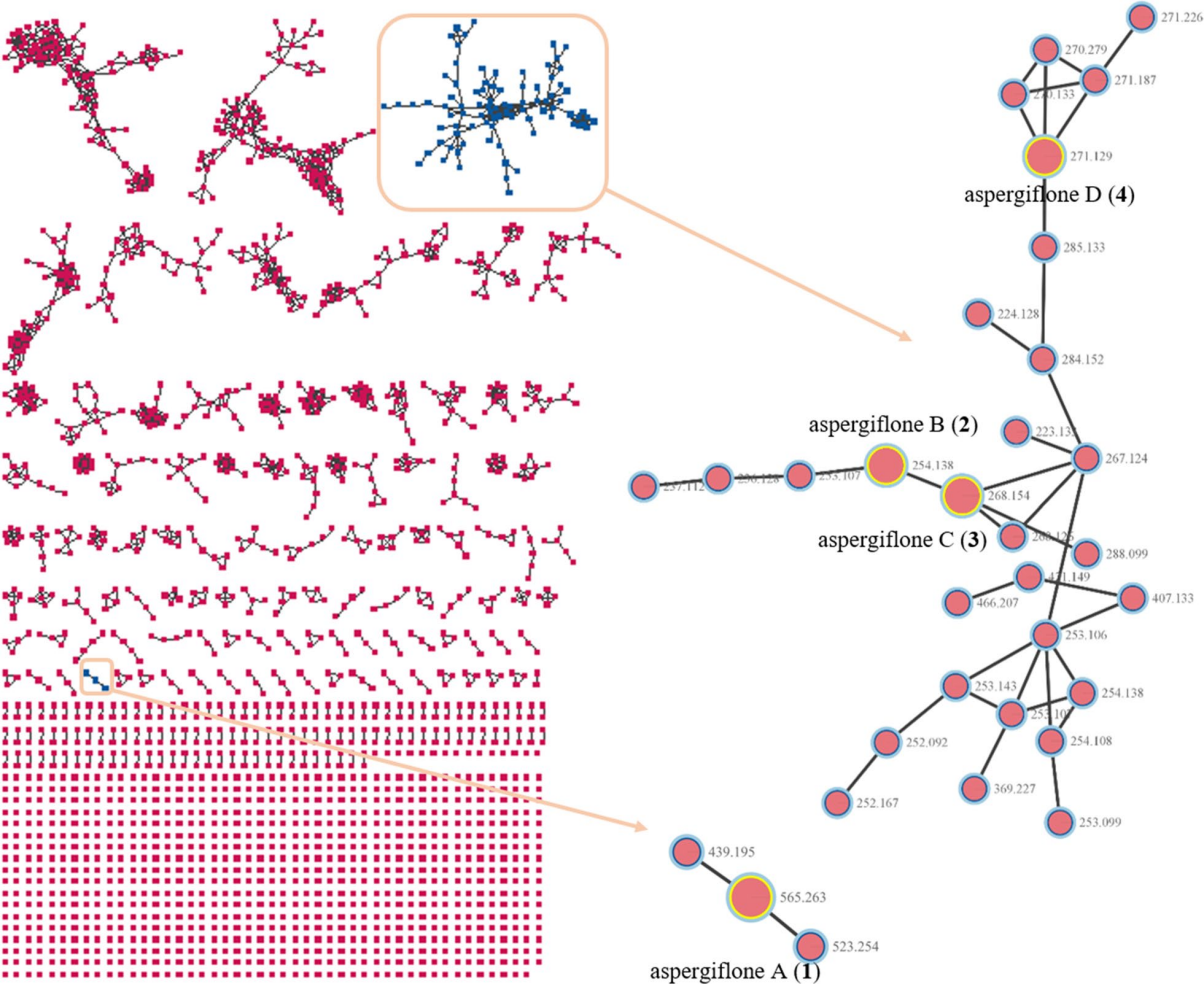
Molecular networks for secondary metabolites of *Aspergillus flavus*

**Fig. 2:**
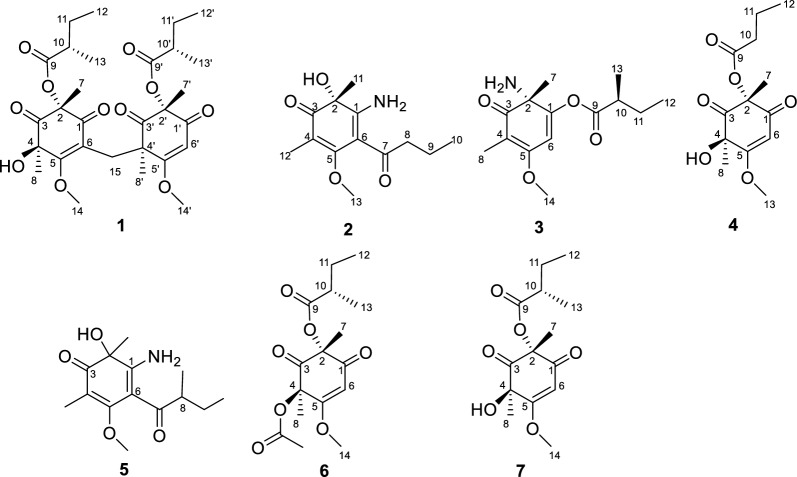
Structures of compounds **1**–**7**

The EtOAc extracts of *Aspergillus flavus* were analyzed using the LC − MS/MS molecular networking. The data were then screened on the Global Natural Product Social Molecular Networking (GNPS) library, with some sample-specific clusters identified (Fig. 1). Based on this observation, the specific fractions were traced through mass spectrometry for separation. This process led to the isolation of four new secondary metabolites (**1–4**) and three known analogues (**5–7**) (Fig. 2).

Aspergiflone A (**1**), was obtained as a colorless oil. The molecular formula was deduced as C_29_H_40_O_11_ based on the HRESIMS ion peak at m/z 565.2638 ([M + H]^+^, calcd for 565.2643), indicating ten degrees of unsaturation. The ^1^H NMR data (Table S1) indicated the presence of ten methyl groups, and one olefinic proton. The ^13^C NMR data (Table S1) and HSQC spectrum revealed 29 carbon signals, assigned to ten methyl carbons (two oxygenated and eight sp^3^-hybridized), three methylene carbons (all sp^3^ hybridized), three methine carbons (one olefinic and two sp^3^-hybridized), and thirteen non-hydrogenated carbons (three olefinic, four six sp^3^-hybridized and six carbonyls). These data collectively suggest that compound **1** possesses a bicyclic framework.

**Fig. 3:**
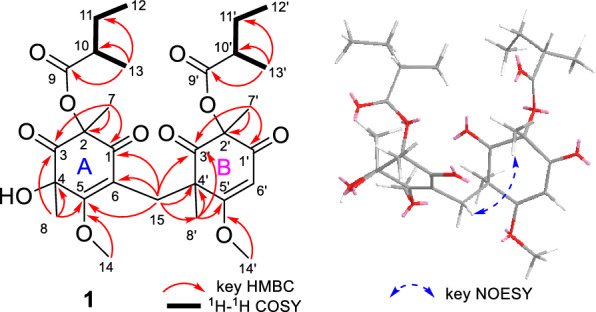
Key HMBC, ^1^H-^1^H COSY and NOESY correlations of** 1**

Analysis of the 1D and 2D NMR data (Table S1 and Fig. 3) revealed that the structure of compound **1** is similar to that of compound **7**, a known cyclohexenone previously isolated from the fungus *Paecilomyces lilacinus* [23]. The primary difference is that in compound **1**, two cyclohexenone residues are connected at C-15 through the C − C single bond, forming a cyclohexenone dimer. This structural variation is evident by the presence of a methylene carbon (*δ*_C_ 31.4) at C-15, which connects rings A and B at C-6 (*δ*_C_ 115.6) and C-4' (*δ*_C_ 53.0), feature absent in compound **7**. These differences are further supported by the HMBC correlations from H_2_−15 to C-1 (*δ*_C_ 191.9), C-5 (*δ*_C_ 168.5), C-6, C-4', C-3' (*δ*_C_ 204.7), and C-8' (*δ*_C_ 21.3).

**Fig. 4:**
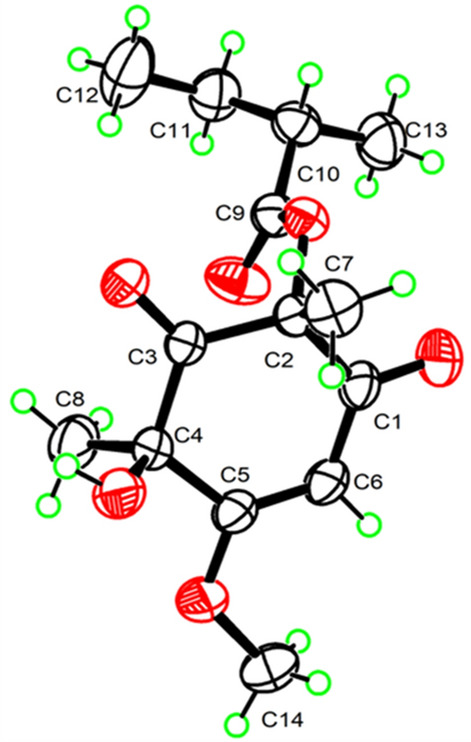
Perspective ORTEP drawings of the X-ray structures of **7** (displacement ellipsoids are drawn at the 50% probability level)

**Fig. 5:**
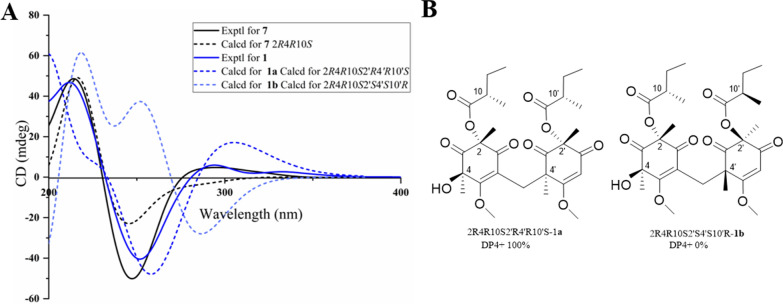
**A** Experimental and calculated ECD spectra of **1** and **7** **B** Results of DP4+ analyses for 2*R*4*R*10*S*2'*R*4'*R*10'*S*-**1**a and 2*R*4*R*10*S*2'*S*4'*S*10'*R*-**1b**

The chemical shifts around the chiral centers at C-2, C-4, C-10, C-2', and C-10' in compound **1** were nearly identical to those of compound **7** (Table S5), for which the absolute configuration was determined as 2*R*4*R*8*S* by single-crystal X-ray diffraction analysis [Flack parameter −0.12(7)] (Fig. 4). This suggested that the relative configuration of C-2, C-4, C-10, C-2', and C-10' in **1** was same as that of **7**. In the NOESY spectrum, correlations between H_3_−7'/H-15a (*δ*_H_ 3.27) suggested the *α*-orientation of H_3_−8' and the *β*-orientation of H_3_−7' (Fig. 3). To elucidate the configuration of the chiral centers within the bicyclic ring system of compound **1**, ECD calculation and DP4+ probability analyses were performed. The ECD spectra of 2*R*4*R*10*S*2'*R*4'*R*10'*S*-**1a**, and 2*R*4*R*10*S*2'*S*4'*S*10'*R* -**1b** were calculated using the time-dependent density functional theory (TDDFT) method at the CAM-B3LYP/6-311G (d, p) level. The experimental ECD spectrum of compound **1** matched the calculated ECD spectrum for the 2*R*4*R*10*S*2'*R*4'*R*10'*S* (Fig. 5A). Furthermore, DP4+ probability analysis (100% for 2*R*4*R*10*S*2'*R*4'*R*10'*S* configurations, Fig. 5B, Table S6 and Fig. S2) based on the gauge-independent atomic orbital (GIAO) method at the PCM/mPW1PW91/6–31 + G (d, p) level supported this relative configuration assignment. Considering the biosynthetic pathway, compound **7** could undergo the methyltransferases and polymerization to form aspergiflone A (**1**) [31–33], further supporting the accuracy of the configuration assignment of **1 (**In Sect. 5 of the Supporting Information).

**Fig. 6:**
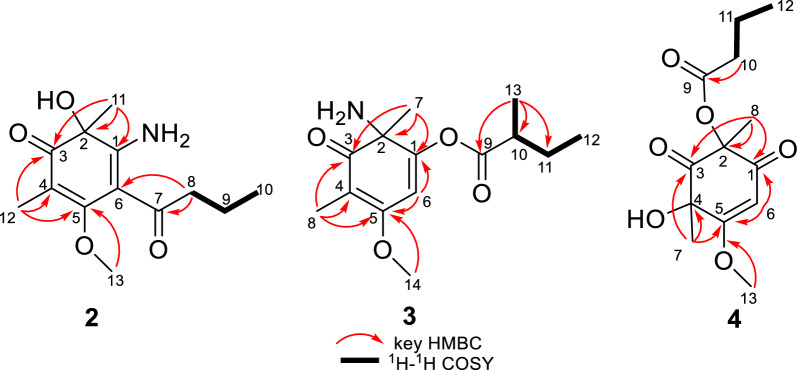
Key HMBC, ^1^H-^1^H COSY, and NOESY correlations of **2–4**

**Fig. 7:**
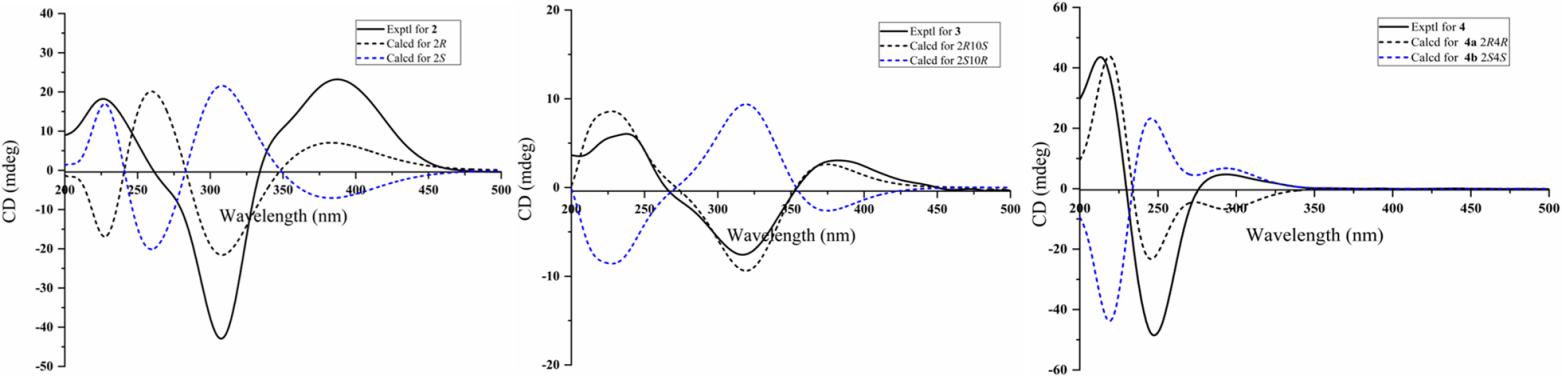
Experimental and calculated ECD spectra of** 2**–**4**

Aspergiflone B (**2**), isolated as a colorless oil, was determined to have the molecular formula C_13_H_19_NO_4_ base on the HRESIMS ion peak at *m/z* 254.1387 ([M + H]^+^, calcd. 254.1387), indicating five degrees of unsaturation. The 1D and 2D NMR data (Table S2 and Fig. 6) of compound **2** closely resembled those of compound **5** [19], with the only difference being the absence of a methyl group at C-8 in** 2**, as supported by the ^1^H-^1^H COSY correlations of H-8/H-9/H-10. Finally, the absolute configuration of **2** was defined as 2*R* in the TDDFT/ECD calculations (Fig. 7).

Aspergiflone C (**3**) was isolated as a colorless oil. The molecular formula C_14_H_21_NO_4_ was determined by the HRESIMS ion peak at m/z 268.1548 ([M + H]^+^, calcd. 268.1543), implying five degrees of unsaturation. The ^1^H NMR data (Table S3) indicated the presence of four methyls groups, one methoxyl group, and one olefinic proton. The ^13^C NMR data (Table S3) and HSQC spectrum revealed the presence of 14 carbons signals, which were classified as five methyl carbons (one oxygenated, one olefinic and three sp^3^-hybridized), one methylene carbons (one sp^3^-hybridized), two methine carbons (one olefinic and one sp^3^-hybridized), and six non-hydrogenated carbons (three olefinic, one sp^3^-hybridized and two carbonyls).

The HMBC correlations (Fig. 6) from H-8 to C-3 (*δ*_C_ 200.0), C-4 (*δ*_C_ 105.6), and C-5 (*δ*_C_ 169.7), as well as from H_3_−14 to C-5, indicated the presence of a carbonyl group at C-3 and a methoxy group at C-5, respectively. Combined with the HMBC correlations from H_3_−7 to C-1 (*δ*_C_ 150.1), C-2 (*δ*_C_ 74.7), and C-3, and from H-6 to C-1, C-5, a cyclohexenone skeleton was established. The presence of a 2-methylbutanoic acid group was inferred from the ^1^H–^1^H COSY correlations of H-12/H-11/H-10/H-13 (Fig. 6), together with the HMBC correlations from H_3_−13 to C-9 (*δ*_C_ 176.2), C-10 (*δ*_C_ 44.3), C-11 (*δ*_C_ 27.4). Based on the molecular formula, the presence of an amino group in compound** 3** was confirmed. A literature survey revealed that the chemical shifts was ranging from *δ*_C_ 160.8 to *δ*_C_ 175.0 when an amino group located at a double bond [13, 19]. Accordingly, the highfield chemical shift of C-1 (*δ*_C_ 150.1) indicated that the amino group was located at C-2 (*δ*_C_ 74.7), rather than at C-1. Thus, the 2-methylbutyric acid group was determined to be attached to C-1 (*δ*_C_ 150.1), which was further supported by its molecular formula and the required degrees of unsaturation.

To elucidate the absolute configuration of C-10, a hydrolysis reaction was performed. Acid hydrolysis of compound **3** yielded 2-methylbutanoic acid, which exhibited a positive optical rotation ($[\alpha]^{25}_{{\rm D}}$ + 9.0, *c* 0.05, MeOH) (Fig. S5). By comparing the optical rotation of the hydrolysate with those reference compounds [14], (+) and (−)−2-methylbutanoic acids, the absolute configuration at C-10 is defined as 10*S.* The DP4+ probability analysis suggested that the 2R*10S*configuration of **3** was in good correlation with the experimental data (Table S7 and Fig. S3). Finally, the absolute configuration of **3** was defined as 2*R*10*S* in the TDDFT/ECD calculations (Fig. 7).

Aspergiflone D (**4**), was obtained as a colorless oil. The molecular formula was deduced as C_13_H_18_O_6_ based on the HRESIMS ion peak at *m/z* 271.1174 ([M + H]^+^, calcd. 271.1176), implying five degrees of unsaturation. The 1D and 2D NMR data (Table S4 and Fig. 6) revealed that compound **4** closely resembled compound **7** [23]. The only difference was that the absence of a methyl at C-10 in** 4** compared to **7**, as confirmed by the ^1^H-^1^H COSY correlations of H-10/H-11/H-12 (Fig. 6). The nearly identical NMR data for the chiral centers in **4** and **7** suggested that the relative configuration of **4** is the same as that of **7**, which was further supported by DP4 + probability analysis (Table S7 and Fig. S3). The absolute configuration of **4** was identified as 2*R*4*R*, rather than 2*S*4*S*, by the TDDFT/ECD calculations (Fig. 7).

Foodborne pathogens are important source of food safety issues and have attracted increasing attention from governments and the public worldwide [34]. The antimicrobial activities of compounds **1**−**6** were evaluated against four foodborne pathogens, including *Staphylococcus aureus*, *Escherichia coli*, *Proteus* sp*,* and *Shigella flexneri.* Compounds **1**−**6** exhibited antibacterial activity against *Staphylococcus aureus*, *Escherichia coli*, and *Proteus* sp with MIC values ranging from 1.0 to 64 μg/mL (Table 1). Among them, compound **1** exhibited antibacterial activity against *Escherichia coli* equivalent to that of the positive control, ciprofloxacin.

**Table 1:** Antimicrobial activity of compounds **1**‒**6** (MIC, *μ*g/mL)

Compound	*Staphylococcus aureus*	*Escherichia coli*	*Proteus* sp	*Shigella flexneri*
**1**	4	1	16	> 64
**2**	4	32	32	> 64
**3**	16	> 64	32	> 64
**4**	16	> 64	> 64	> 64
**5**	64	2	16	> 64
**6**	> 64	16	> 64	> 64
ciprofloxacin	0.5	1	0.5	4

**Fig. 8:**
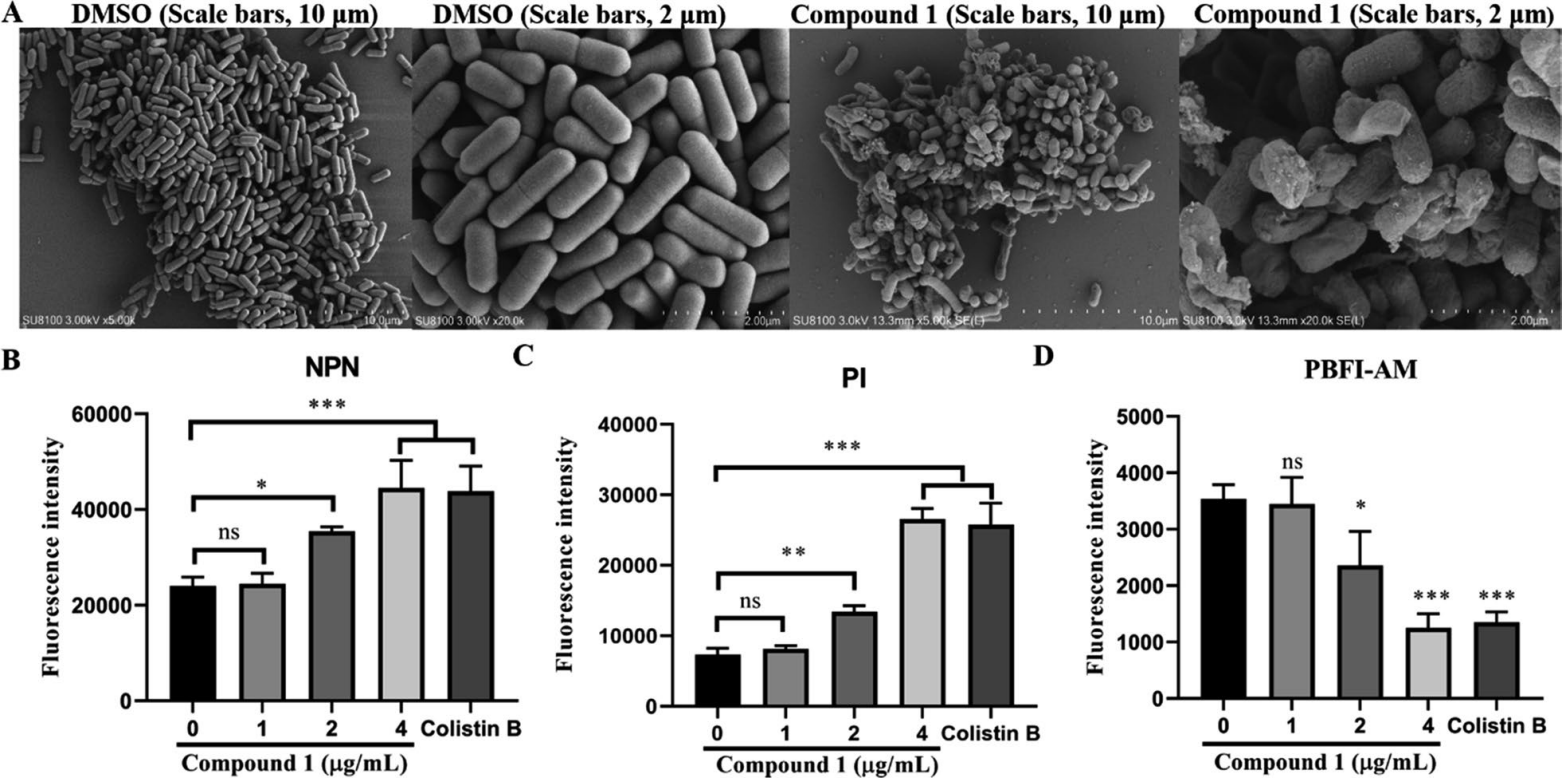
**A** SEM images of *Escherichia coli* hyphae treated by DMSO and compound **1.** Scale bars, 10 μm and 2 μm, respectively. **B** The outer membrane of Escherichia coli by measuring fluorescence intensity of NPN treated with increasing concentrations of compound** 1**. **C** The inner membrane of *Escherichia coli* by measuring fluorescence intensity of PI treated with increasing concentrations of compound **1**. **D** Intracellular changes of K^+^ probed with the K^+^-sensitive dye PBFI treated with increasing concentrations of compound **1**. Data represented as mean ± standard deviation (SD). *P < 0.05, **P < 0.01, ***P < 0.01. compared to the model group, respectively

To investigate the antibacterial mechanism of compound **1**, scanning electron microscope (SEM) was utilized, with dimethyl sulfoxide (DMSO) serving as the blank control. The surface morphology of cell structure was observed and analyzed under a microscope. As shown in Fig. 8A, the cell membrane of *Escherichia coli* exhibited a neat arrangement, plump structure, intact shape, and smooth surface. However, following treatment with compound** 1**, the hyphal surface appeared contracted, rough, and irregularly intertwined, indicating that compound** 1** can damage the ultrastructure of fungal hyphae.

Cell membrane, as an important barrier, retains cellular constituents while removing unwanted substances [35]. Many antimicrobial compounds have been reported to increase cell membrane permeability [36, 37]. Therefore, the outer membrane (OM) permeability and integral membrane (IM) permeability of bacteria were assessed using 1-N-phenylnaphthylamine (NPN) and propidium iodide (PI) fluorescence probe analysis, respectively. NPN, a hydrophobic fluorescent probe that emitted fluorescence upon interacting interacts with the hydrophobic regions of the phospholipid bilayer, was used to monitor the permeability of the OM [38]. PI, a cell impermeable nucleic acid intercalating dye that can penetrate only dead or damaged cells, was employed to assess integrity of the bacterial inner membrane (IM) [38].

As shown in Fig. 8B and C, compound **1** dose-dependently increased fluorescence intensity in both probe analyses, indicating that compound **1** could enhance the permeability of both the OM and IM of the bacterium. To further investigate the effect of compound **1** on cell membrane function, a potassium (K^+^) indicator fluorescent dye was used to assess K^+^ flux after treatment with different concentrations of compound **1** [39]. As shown in Fig. 8D, compound **1** dose-dependently decreased intracellular potassium levels, indicating that compound **1** disrupts the cell membrane by affecting K^+^ ions flux.

**Fig. 9:**
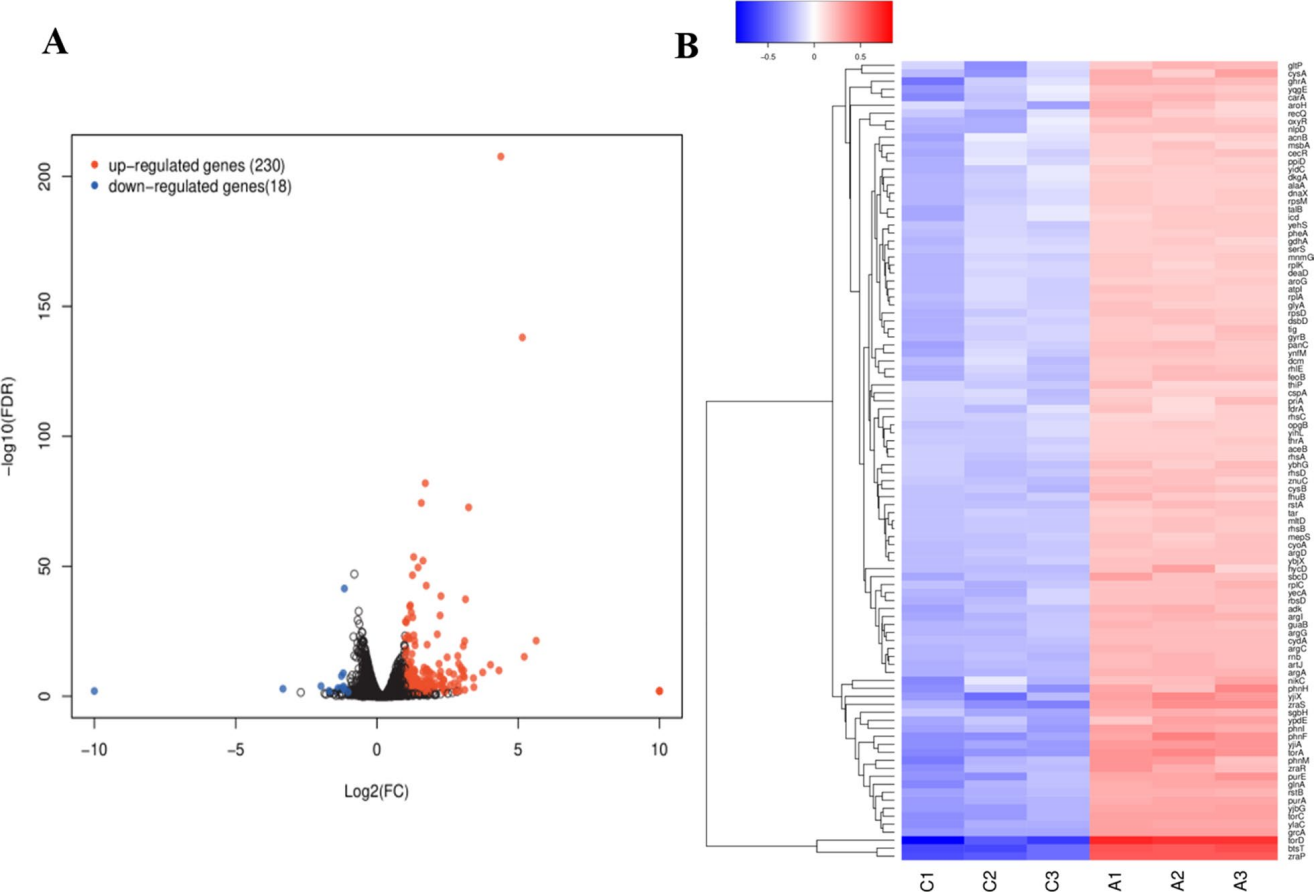
**A** Transcriptome analysis of *Escherichia coli* after treatment with compound **1**. **A** Volcano plot of expression of genes in *Escherichia coli* after treatment with compound **1**. Blue dots indicate downregulated genes, and red dots indicate upregulated genes. **B** Clustered heatmap of DEGs, with red cluster representing up-regulated and blue cluster representing down-regulated. The color from blue to red indicates higher gene expression

In order to better understand the potential antibacterial mechanism of compound **1** against *Escherichia coli* in gene expression, strand-specific prokaryotic transcriptome sequencing was performed [40]. By comparing the transcriptomic analysis of the control and treatment groups, 248 differentially expressed genes (DEGs) were identified in samples after FCS treated (|log2 (fold change)|> 1, FDR < 0.05), consisting of 230 upregulated genes and 18 downregulated genes (Fig. 9A and Table S10). The higher proportion of DEGs in *Escherichia coli* indicated its more cellular response to compound **1**, and this result was consistent with the high antibacterial activity of compound **1** against *Escherichia coli*. Additionally, the cluster analysis of DEGs after FCS treatment indicated that the gene expression patterns in samples were also mostly up-regulated (Fig. 9B), indicating that the treatment with compound **1** might target specific biological processes or pathways within *Escherichia coli* [41].

**Fig. 10:**
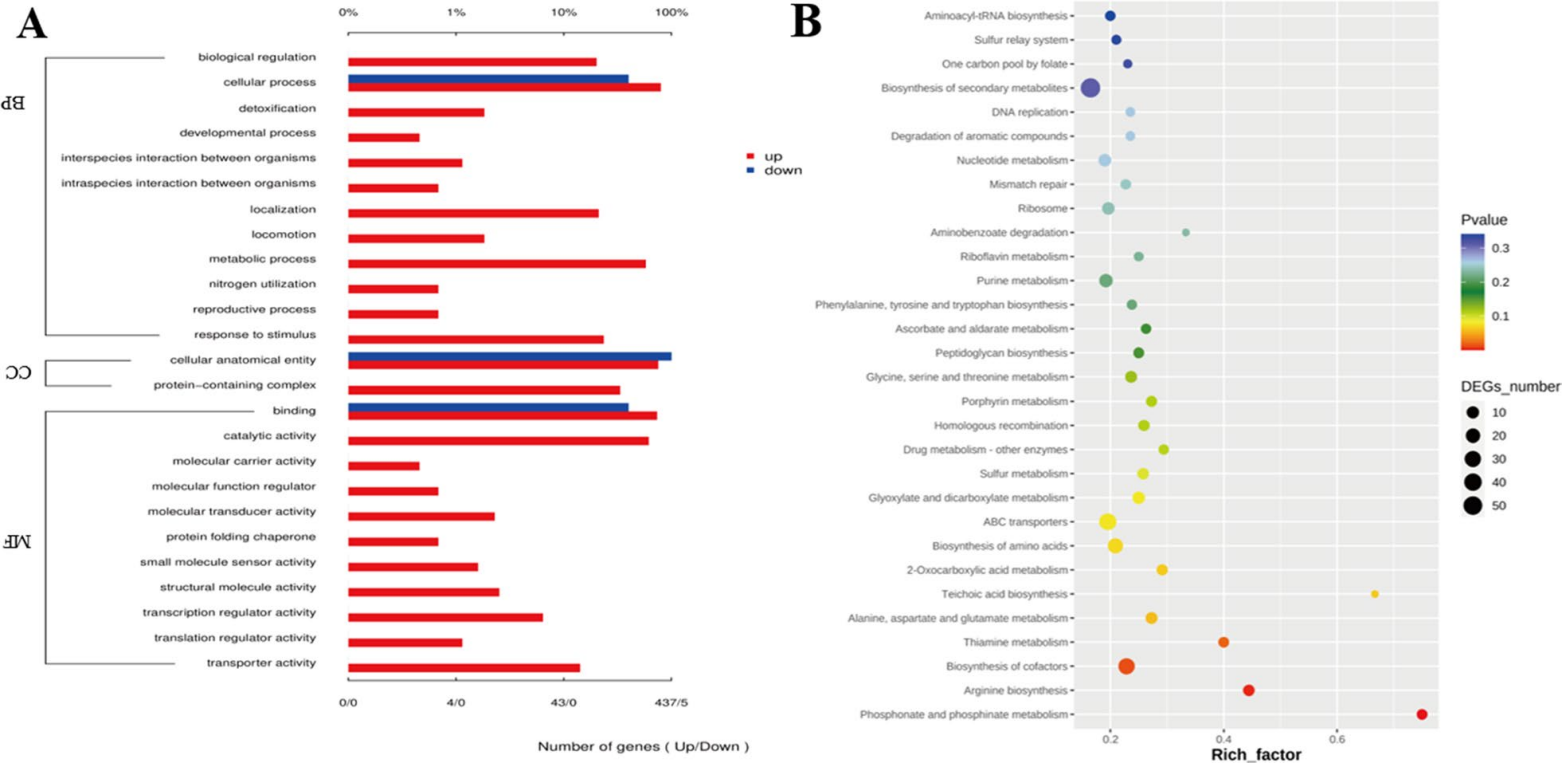
**A** Go enrichment analysis of DEGs annotated in three main categories: molecular function (MF), biological process (BP), and cellular component (CC). **B** KEGG pathway enrichment analysis of DEGs in *Escherichia coli* after treatment with compound **1**

Gene Ontology (GO) analysis, an internationally standardized system for classifying gene function, provides a controlled vocabulary and strictly defined concepts to describe gene functions and gene products in any organism [42]. As shown in Fig. 10A, DEGs annotated by GO enrichment analysis were further characterized into three categories, including biological process (BP), cellular component (CC) and molecular function (MF). Regarding the category of BP, the DEGs were mainly distributed in cellular process, biological regulation, response to stimulus, and metabolic process. For the category of CC, the DEGs were mainly distributed in cellular anatomical entity and protein-containing complex. With the category of MF, the main groups that DEGs distributed were binding, catalytic activity, transporter activity, and transcription regulator activity. The results revealed that many genes of *Escherichia coli*. involved in biological process, cellular component, and molecular function were altered, especially key functional modules in cellular process, transportation, binding, catalytic activity, and response to stimulus up-regulated, suggesting that exposure to compound **1** affected the metabolism and activity of *Escherichia coli*.

Systematic research on biological pathways is important for understanding and facilitating genomics research. Kyoto Encyclopedia of Genes and Genome (KEGG) enrichment analysis was further performed to categorize DEGs into different pathways, which could reveal the functional information and relationship at the molecular, cellular, and organism levels [43]. As shown in Fig. 10B, the results indicated that ABC transporters were the most enriched pathways in DEGs. The amino acid biosynthesis pathways including arginine biosynthesis, alanine, aspartate and glutamate metabolism, glycine, serine and threonine metabolism, sulfur metabolism were enriched. Other major pathways related to energy metabolism and cofactor synthesis involving in thiamine metabolism, biosynthesis of cofactors, phosphonate and phosphinate metabolism were enriched, together with pathways of DNA damage, repair involving in DNA replication, mismatch repair, homologous recombination enriched. The results above suggested that these pathways played important roles in antibacterial processes of *E. coli.*

## Conclusion

To the best of our knowledge, previous studies have reported over one hundred cyclohexenone derivatives [13–27]. In the study presented here, guided by the GNPS molecular networking strategy, four new cyclohexenones (**1**–**4**) were isolated from a coral-derived fungus *Aspergillus flavu*s. Structurally, aspergiflone A (**1**) is a rare cyclohexenone dimer, in which two cyclohexenone units are connected at C-15 through a C−C single bond, representing a novel carbon skeleton. Compound **1** showed strong antibacterial activity against *Escherichia coli*. The preliminary analysis of its mechanism of action suggested that compound **1** obviously disrupted the morphological integrity of the mycelium, enhanced the permeability of the cell membrane, and decreased potassium levels. The transcriptome analysis at gene level revealed DEGs mostly related to regulation of cellular process, stimulus response, catalytic and binding functions, and KEGG pathways involved in ABC transporters, energy metabolism, biosynthesis of cofactors, amino acid biosynthesis were affected, resulted in obvious antibacterial effect of compound **1** in *Escherichia coli*. In terms of structure-effective relationship, the cyclohexenone dimer appears to potentiate the antibacterial effect against *Escherichia coli*. These results provided a guide for the exploitation and application of this cyclohexenone derivative as an antibacterial candidate in food preservation technology.

## Experimental section

### General experimental procedures

Instructions of Optical rotations were measured using a Jasco P-1020 digital polarimeter. Ultraviolet (UV) spectra were recorded on a Beckman DU640 spectrophotometer. Circular dichroism (CD) spectra were measured using a Jasco J-810 spectropolarimeter. NMR spectra were measured using an Agilent spectrometer 500 MHz. The 7.26 ppm (^1^H) resonance of residual CHCl_3_ in CDCl_3_ and the 77.16 ppm (^13^C) resonance of CDCl_3_ were used as internal references for ^1^H and ^13^C NMR spectra, respectively. Crystallographic data were collected on a Bruker D8 Venture diffractometer (Bruker, Beijing, China) equipped with graphite-monochromatized Cu Kα radiation. High-resolution electrospray ionization mass spectrometry (HRESIMS) spectra were measured using a Micromass Q-Tof Ultima GLOBAL GAA076LC mass spectrometer. Semi-preparative HPLC was performed using a Waters 1525 pump equipped with a 2998 photodiode array detector and a YMC C18 column (YMC, 10 × 250 mm, 5 μm). For column chromatography, silica gel (200 − 300 mesh, 300 − 400 mesh, and H) was utilized.

### Fungal material

The fungal strain SY-61-WM was isolated from an unidentified soft coral (SY-2023–61) collected from the Xisha Islands (Yalong Bay) of the South China Sea in November 2023 (109°29′E, 18°13′N). The strain was identified as *Aspergillus flavus* based on the sequenced ITS region (GenBank accession no. ASM901741v1), with 100% similarity to *A. flavus*. The strain has been deposited at the State Key Laboratory of Marine Drugs, Ocean University of China, People’s Republic of China.

### Fermentation, extraction, and isolation

The fungal strain SY-61-WM was cultured on potato dextrose agar (PDA) at 28 °C for 3 days to prepare the seed culture. Agar plugs were then inoculated into 200 × 0.5 L Erlenmeyer flasks, each containing 120 mL of seawater supplemented with glucose 30 g/L, yeast extract 2 g/L, peptone 5 g/L, and KH_2_PO_4_ 0.5 g/L. These flasks were incubated at 28 °C for 33 days.

The fermented broth was macerated with EtOAc (60 L × 5). The combined EtOAc extracts were concentrated in vacuo and desalted by redissolving in MeOH, yielding a residue (90.0 g). The crude extract was subjected to silica gel vacuum column chromatography, eluted with a gradient of petroleum ether/ethyl acetate (300:1–1:1, *v/v*), followed by a gradient of CH_2_Cl_2_/MeOH (10:1–1:1, *v/v*), to obtain fourteen fractions (Fr.1–Fr.14), as monitored by TLC. Fr.6 was further fractionated by silica gel vacuum column chromatography (petroleum ether/ethyl acetate, 10:1 to 1:1, *v/v*) to give four subfractions Fr.6.1–Fr.6.4. Fr.6.2 was separated by semi-preparative HPLC (ODS, 5 µm, 250 × 10 mm; CH_3_OH/H_2_O, 55:45, *v/v*; 1.5 mL/min) to afford **5** (30.3 mg, *t*_R_ = 15 min). Fr.6.3 was separated by semi-preparative HPLC (ODS, 5 µm, 250 × 10 mm; CH_3_OH/H_2_O, 50:50, *v/v*; 1.5 mL/min) to afford **2** (2.2 mg, *t*_R_ = 20 min) and **3** (3.3 mg, *t*_R_ = 35 min). Fr.6.4 was separated by semi-preparative HPLC (ODS, 5 µm, 250 × 10 mm; CH_3_OH/H_2_O, 50:50, *v/v*; 1.5 mL/min) to afford **7** (4.5 mg, *t*_R_ = 35 min). Fr.8 was subjected to a silica gel vacuum column chromatography (petroleum ether/ethyl acetate, 4:1, *v/v*) to give seven subfractions Fr.8.1–Fr.8.7. Fr.8.5 was purified by semi-preparative HPLC (ODS, 5 µm, 250 × 10 mm; CH_3_OH/H_2_O, 60:40, *v/v*; 2 mL/min) to afford **4** (2.1 mg, *t*_R_ = 23 min) and **6** (5.6 mg, *t*_R_ = 30 min). Fr.8.7 was separated by semi-preparative HPLC (ODS, 5 µm, 250 × 10 mm; CH_3_OH/H_2_O, 55:45, *v/v*; 2 mL/min) to afford **1** (5.0 mg, *t*_R_ = 70 min).

Aspergiflone A (**1**). Colorless oil; $[\alpha]^{25}_{{\rm D}}$ + 67.7 (*c* 1.0, MeOH); ECD (*c* 0.5, MeOH) = Δε210 + 52.7, Δε250 − 46.5, Δε290 + 6.9; UV (MeOH) *λ*max (log *ε*) = 258 (1.57) nm; IR (KBr) *ν*max = 3625, 2831, 2509, 1751, 1740, 1722, 1595, 1364, 775 cm^−1^; HRESIMS *m/z* 565.2638 [M + H]^+^ (calcd. for C_29_H_41_O_11_, 565.2643); For ^1^H NMR and ^13^C NMR data see Table S1.

Aspergiflone B (**2**). Colorless oil; $[\alpha]^{25}_{{\rm D}}$ + 90.3 (*c* 1.0, MeOH); ECD (*c* 0.5, MeOH) = Δε228 + 17.8, Δε308 −48.2, Δε385 + 20.2; UV (MeOH) *λ*max (log *ε*) = 250 (1.67) nm; HRESIMS *m/z* 254.1387 [M + H]^+^ (calcd. for C_13_H_20_NO_4_, 254.1387); IR (KBr) *ν*max = 3450, 2823, 1721, 1700, 774 cm^−1^; For ^1^H NMR and ^13^C NMR data see Table S2.

Aspergiflone C (**3**). Colorless oil; $[\alpha]^{25}_{{\rm D}}$ + 80.7 (*c* 0.5, MeOH); ECD (*c* 0.5, MeOH) = Δε228 + 6.5, Δε316 −8.7, Δε382 + 3.07; UV (MeOH) *λ*max (log *ε*) = 250 (2.50) nm; HRESIMS *m/z* 268.1548 [M + H]^+^ (calcd. for C_14_H_22_NO_4_, 268.1543); IR (KBr) *ν*max = 2779, 1643, 1600, 1371, 721 cm^−1^; For ^1^H NMR and ^13^C NMR data see Table S3.

Aspergiflone D (**4**). Colorless oil; $[\alpha]^{25}_{{\rm D}}$ −13.9 (*c* 0.5, MeOH); ECD (*c* 0.5, MeOH) = Δε212 + 44.8, Δε245 −66.2; UV (MeOH) *λ*max (log *ε*) = 248 (2.61) nm; IR (KBr) *ν*max = 3545, 2778, 1741, 1619, 1364, 774 cm^−1^; HRESIMS *m/z* 271.1174 [M + H]^+^ (calcd. for C_13_H_19_O_6_, 271.1176); For ^1^H NMR and ^13^C NMR data see Table S4.

### Molecular networking

The EtOAc extract from *Aspergillus flavus* were tested by LC–MS/MS analysis using an Q Exactive Orbitrap system equipped with a diode array detector. Separation was achieved on a 250 × 2.1 mm i.d., 2.2 μm, Thermo Fisher Acclaim RSLC 120 C_18_ column. A gradient of MeOH/H_2_O from 5:95 to 100:0 in 97 min was used for analysis condition. The ESI dual source, Full MS-ddMS2 (DDA) mode, and positive ion mode were employed. MS scans were operated from m/z 50−1500. And auto MS/HRMS fragmentation was performed at three collision energies (30, 40, and 50 eV). A mass tolerance of 0.02 Da was set for the MS/MS fragment ion and the precursor ion. The MS/MS data were converted to mzXML file format using MS-Convert and were deposited in GNPS Web platform (http://gnps.ucsd.edu/ProteoSAFe/status.jsp?task=eb4c402362ef46e1a4a52820eb48a39b) for molecular networking analysis.

### Antimicrobial screening

Antibacterial activity against *Staphylococcus aureus BNCC*186335, *Escherichia coli* BNCC133264, *Proteus sp* BNCC107965 and *Shigella flexneri* BNCC186377 was evaluated using the agar diffusion method. The tested strains were cultured in liquid LB medium at 28 °C for 12 h. Compounds **1**−**6** and the positive control, ciprofloxacin, were dissolved in methanol at a concentration of 0.1 mg/mL, and inoculated with 5 × 10^5^ CFU/mL of pathogenic cells. The minimum inhibitory concentrations (MICs) were determined using the broth microdilution method, defined as the lowest concentration that resulted in complete inhibition of bacterial growth. Ciprofloxacin was used as the positive control. All assays were performed in triplicate.

### Mycelial morphology observation of Escherichia coli

Compound **1** was dissolved to a concentration of 4 μg/mL. A solvent blank control group and three parallel experimental groups were established. Each Petri dish containing the medium was inoculated with a bacterial culture, by placing a single bacterial plug in each dish. The hyphal morphology was then observed using a SEM SU8100 (Hitachi, Ltd., Tokyo, Japan).

### Detection of cell membrane permeability

1-N-phenylnaphthylamine (NPN) and propidium iodide (PI) fluorescence probes were used to assess the OM permeability and IM permeability. Bacteria cells were mixed with PI (0.5 μM) and NPN (10 μM) probes (MedChemExpress, Shanghai, China), respectively, and incubated in the dark for 30 min. Colistin B was used as a positive control. After incubation, 19 *μ*L of bacterial suspension was mixed with 10 *μ*L of compound **1** in a 96-well black plate. Following an additional 2 h incubation in the dark, the NPN fluorescence intensity was measured using an excitation wavelength of 350 nm and an emission wavelength of 420 nm. And the PI fluorescence intensity was measured using an excitation wavelength of 535 nm and an emission wavelength of 615 nm.

### Mitochondrial potassium uptake

Bacteria cells were mixed with the K^+^-sensitive fluorescent indicator PBFI AM (Maokangbio, Shanghai, China, 10 μM) using a loading protocol that allowed exclusive labeling of the mitochondria, and incubated in the dark for 30 min. Colistin B was used as a positive control. After incubation, 190 *μ*L of the bacterial suspension was mixed with 10 *μ*L of compound **1** in a 96-well black plate. Fluorescence intensity was measured using an excitation wavelength of 340 nm and an emission wavelength of 500 nm.

### Transcriptomic analysis

*Escherichia coli* was grown overnight in MHB to the exponential phase at 37 °C. Then, cells were incubated with compound 1 (4 µg mL − 1) for 2 h. The bacteria were washed by fresh PBS for three times, then the bacteria were centrifuged at 12000 r.p.m. for 10 min at 4 °C. The deep sequencing was performed by Ling En Biotechnology Co., Ltd. RNA purity and concentration were measured using NanoDrop 2000 spectrophotometers (Thermo Fisher Scientific). RNA integrity was analysed using Agilent Bioanalyzer 2100 (Agilent Technologies). Ribosomal RNA was removed using Epicentre Ribo ZeroTM rRNA Removal Kits (bacteria). Subsequently, the sequencing libraries were constructed using the VAHTS Total RNA-seq Library Prep Kit for Illumina (Vazyme, catalogue no. NR603). First-strand cDNA was synthesized using reverse transcriptase, and strand specificity was obtained by replacing dTTP with dUTP in the second-strand marking buffer. Following this, the cDNA fragments were end repaired with the addition of a single ‘A’ base at the 3’-end of each strand. All the analyses were performed using the free online platform of Majorbio Cloud Platform (Majorbio, Shanghai, China). Each experiment was performed in triplicate. Differentially expressed genes (DEGs) were conducted using DEseq2 (|log2 (fold change)|> 1, FDR < 0.05). The ClusterProfiler was used to analyze Gene Ontology (GO) and Kyoto Encyclopedia of Genes and Genomes (KEGG) analyses.

## Supplementary Information

Additional file 1: The sequenced ITS region of *Aspergillus flavus*; Crystal data and structure refinement for compound **7**; NMR data assignments of **1**–**4**; The determination of relative and absolute configuration for compounds **1**–**4**; Plausible biosynthetic pathway of **1**; Hydrolysis of **3** for determination of absolute configuration; Computational details; The 1D and 2D NMR spectra of **1**–**4**. Crystal data of compound **7.**

Additional file 2: Description detailed of DEGs

## Data Availability

The experimental data supporting this work are accessible within the article and its Additional file.
