# Supplementary material for: Four new Cyclohexenone with antibacterial activity from the coral-derived fungus Aspergillus flavus
Source: Nat Prod Bioprospect. 2026 May 12;16(1):64. doi: 10.1007/s13659-026-00618-y (PMC13161379; doi:10.1007/s13659-026-00618-y)
Supplement: Supplementary file 1 — Additional file 1: The sequenced ITS region of Aspergillus flavus; Crystal data and structure refinement for compound 7; NMR data assignments of 1–4; The determination of relative and absolute configuration for compounds 1–4; Plausible biosynthetic pathway of 1; Hydrolysis of 3 for determination of absolute configuration; Computational details; The 1D and 2D NMR spectra of 1–4. Crystal data of compound 7. [file 13659_2026_618_MOESM1_ESM.doc]

**Supporting Information for Original article**

**Four New Cyclohexenone with Antibacterial Activity from the Coral-derived Fungus *Aspergillus flavus***

Cili Wang ^a,b,c‡^, Jiarui Zhang ^a,c‡^, Lei Li ^a,c^, Sen Wang ^a,c^, Kai Li ^a,c^, Hu Hou ^b,c,*^, Pinglin Li ^a,c,*^

^a^ Key Laboratory of Marine Drugs, Chinese Ministry of Education, School of Medicine and Pharmacy, Ocean University of China, Qingdao 266003, China

^b^ Key Laboratory of Marine Food Processing & Safety Control, College of Food Science and Engineering, Ocean University of China, Qingdao 266003, China

^c^ Laboratory for Marine Drugs and Bioproducts, Qingdao Marine Science and Technology Center, Qingdao 266237, China

^*^ Correspondence: houhu@ouc.edu.cn (Hu Hou); lipinglin@ouc.edu.cn (Ping-Lin. Li).

^‡^ These authors contributed equally to this work.

Table

[1. The sequenced ITS region of *Aspergillus flavus* 3](#__RefHeading___Toc215075380)

[2. Crystal data and structure refinement for compound **7** 4](#__RefHeading___Toc215075381)

[3. NMR data assignments of **1**‒**4**. 5](#__RefHeading___Toc215075382)

[4. The determination of relative and absolute configuration for compounds **1**‒**4** 10](#__RefHeading___Toc215075383)

[4.1 Conformational search 10](#__RefHeading___Toc215075384)

[4.2 Quantum chemical NMR calculation 10](#__RefHeading___Toc215075385)

[4.3 Elucidation of absolute configuration by TDDFT-ECD 15](#__RefHeading___Toc215075386)

[5. Plausible biosynthetic pathway of **1** 16](#__RefHeading___Toc215075387)

[6. Hydrolysis of 3 for determination of absolute conﬁguration 17](#__RefHeading___Toc215075388)

[7. Reference 18](#__RefHeading___Toc215075417)

[8. Computational details 19](#__RefHeading___Toc215075418)

[9. The 1D and 2D NMR spectra of **1**–**4** 32](#__RefHeading___Toc215075419)

1. The sequenced ITS region of *Aspergillus flavus*

AGGACTTACGAGTGTAGGGTTCTAGCGAGCCCAACCTCCCACCCGTGTTTACTGTACCTTAGTTGCTTCGGCGGGCCCGCCATTCATGGCCGCCGGGGGCTCTCAGCCCCGGGCCCGCGCCCGCCGGAGACACCACGAACTCTGTCTGATCTAGTGAAGTCTGAGTTGATTGTATCGCAATCAGTTAAAACTTTCAACAATGGATCTCTTGGTTCCGGCATCGATGAAGAACGCAGCGAAATGCGATAACTAGTGTGAATTGCAGAATTCCGTGAATCATCGAGTCTTTGAACGCACATTGCGCCCCCTGGTATTCCGGGGGGCATGCCTGTCCGAGCGTCATTGCTGCCCATCAAGCACGGCTTGTGTGTTGGGTCGTCGTCCCCTCTCCGGGGGGGACGGGCCCCAAAGGCAGCGGCGGCACCGCGTCCGATCCTCGAGCGTATGGGGCTTTGTCACCCGCTCTGTAGGCCCGGCCGGCGCTTGCCGAACGCAAATCAATCTTTTCCAGGTTGACCTCGGATCACGTAGGGATACCCGCTGAACTTAAGCATATCATAGCCGGGAGGAA

1. Crystal data and structure refinement for compound 7

| Identification code | compound 7 |
| --- | --- |
| Empirical formula | C_14_H_20_O_6_ |
| Formula weight | 284.30 |
| Temperature/K | 291 K |
| Space group | P 1211 |
| a/Å | 9.5470(9) |
| b/Å | 7.3936(7) |
| c/Å | 11.2182(10) |
| α/° | 90 |
| β/° | 107.588(5) |
| γ/° | 90 |
| Volume/Å^3^ | 754.84(12) |
| Z | 2 |
| ρ_calc_g/cm^3^ | 1.251 |
| Mu/mm^‑1^ | 0.820 |
| F (000) | 304.0 |
| Radiation | Cu Kα (λ = 1.54178) |
| Goodness-of-fit on F^2^ | 0.853 |
| Final R indexes [I>=2σ (I)] | R_1_ = 0.0312, wR_2_ = 0.853 |
| Final R indexes [all data] | R_1_ = 0.0373, wR_2_ = 0.1039 |
| Largest diff. peak/hole / e Å^-3^ | 0.71/-0.29 |
| Flack parameter | -0.12(7) |


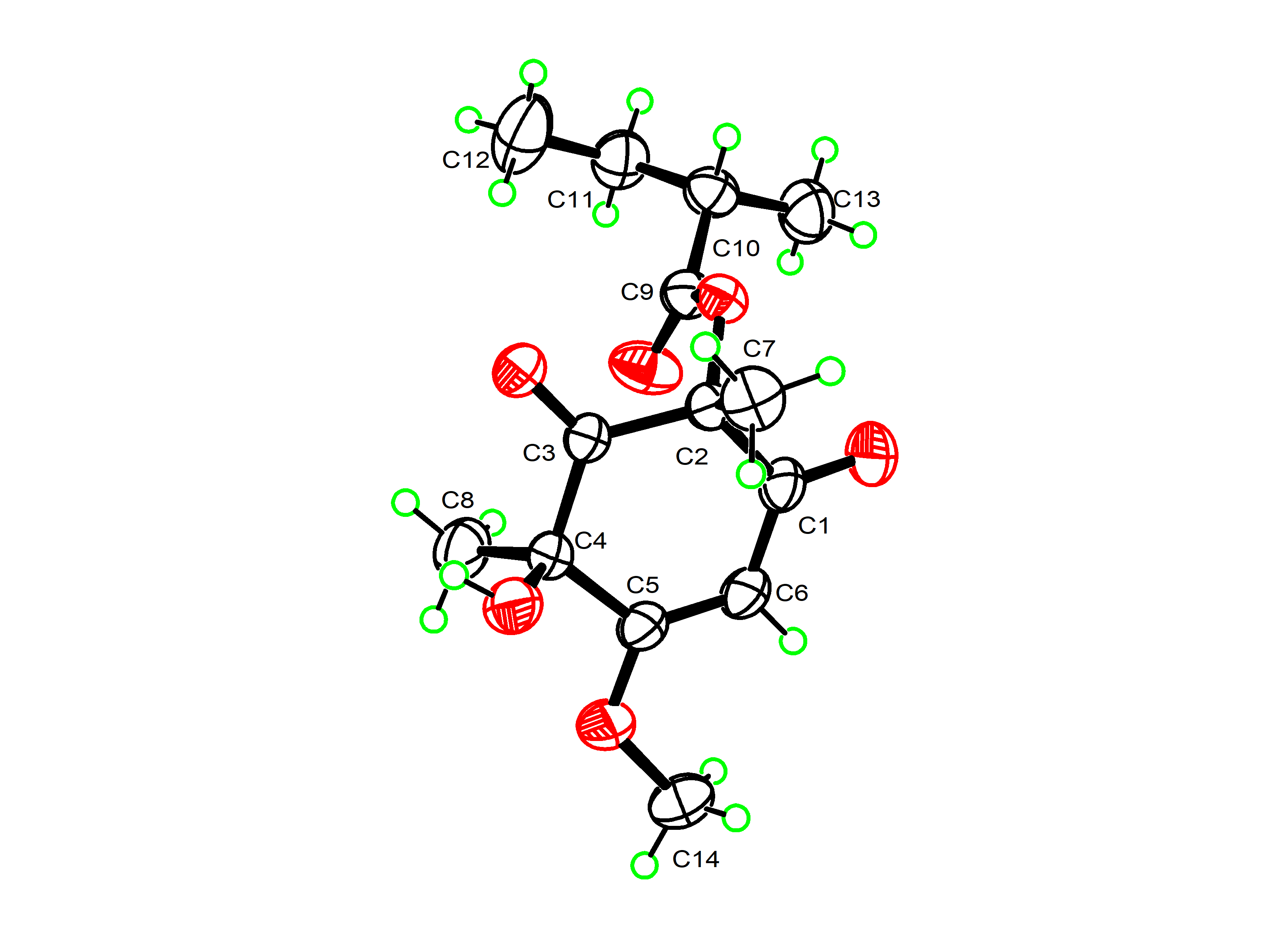


**Figure S1**. Perspective ORTEP drawings of the X-ray structures of **7** (displacement ellipsoids are drawn at the 50% probability level) (Deposition number: CCDC 2455380).

1. NMR data assignments of 1‒4.

**Table S1.** 1D and 2D NMR data of aspergiflone A (**1**)

| **No.** | **1^a^** | | | | |
| --- | --- | --- | --- | --- | --- |
|  | ***δ*_C_^b^, type** | ***δ*_H_^c^ (*J* in Hz)** | **^1^H-^1^H COSY** | **HMBC** | **NOESY** |
| 1 | 191.9, C |  |  |  |  |
| 2 | 80.7, C |  |  |  |  |
| 3 | 205.0, C |  |  |  |  |
| 4 | 75.4, C |  |  |  |  |
| 5 | 168.5, C |  |  |  |  |
| 6 | 115.6, C |  |  |  |  |
| 7 | 24.1, CH_3_ | 1.73, s |  | C-1, C-2, C-3 |  |
| 8 | 26.3, CH_3_ | 1.75, s |  | C-3, C-4, C-5 |  |
| 9 | 175.8, C |  |  |  |  |
| 10 | 40.0, CH | 2.49, m | H-11a, H-11b, H-13 |  |  |
| 11a | 26.9, CH_2_ | 1.70, m | H-10, H-12 |  |  |
| 11a |  | 1.50, m | H-10, H-12 |  |  |
| 12 | 11.5, CH_3_ | 0.95, m | H-11a, H-11b |  |  |
| 13 | 16.4, CH_3_ | 1.16, t, (7.0) |  | C-9, C-10, C-11 |  |
| 14 | 60.5, CH_3_ | 4.13, s |  |  |  |
| 15a | 31.4, CH_2_ | 3.27, d, (13.5) |  | C-1, C-5, C-6, C-3', C-4', C-8' |  |
| 15b |  | 2.69, d, (13.5) |  | C-1, C-5, C-6, C-3', C-4', C-8' |  |
| 1' | 192.9, C |  |  |  |  |
| 2' | 82.0, C |  |  |  |  |
| 3' | 204.7, C |  |  |  |  |
| 4' | 53.0, C |  |  |  |  |
| 5' | 177.9, C |  |  |  |  |
| 6' | 99.1, CH | 5.54, s |  | C-1', C-5' |  |
| 7' | 23.5, CH_3_ | 1.67, s |  | C-1', C-2', C-3' |  |
| 8' | 21.3, CH_3_ | 1.37, s |  | C-3', C-4', C-5' |  |
| 9' | 175.8, C |  |  |  |  |
| 10' | 40.1, CH | 2.49, m | H-11'a, H-11'b, H-13 |  |  |
| 11'a | 26.8, CH_2_ | 1.70, m | H-10', H-12' |  |  |
| 11'b |  | 1.50, m | H-10', H-12' |  |  |
| 12' | 11.5, CH_3_ | 0.95, m | H-11'a, H-11'b |  |  |
| 13' | 16.3, CH_3_ | 1.16, t, (7.0) | H-10' | C-9', C-10', C-11' |  |
| 14' | 56.7, CH_3_ | 3.74, s |  |  |  |

^a^ In chloroform-*d*. ^b^ Recorded at 125 MHz. ^c^Recorded at 500 MHz.

**Table S2.** 1D and 2D NMR data of aspergiflone B (**2**)

| **No.** | **2^a^** | | | | |
| --- | --- | --- | --- | --- | --- |
|  | ***δ*_C_^b^, type** | ***δ*_H_^c^ (*J* in Hz)** | **^1^H-^1^H COSY** | **HMBC** | **NOESY** |
| 1 | 171.4, C |  |  |  |  |
| 2 | 76.3, C |  |  |  |  |
| 3 | 199.9, C |  |  |  |  |
| 4 | 107.1, C |  |  |  |  |
| 5 | 172.8, C |  |  |  |  |
| 6 | 99.7, C |  |  |  |  |
| 7 | 200.4, C |  |  |  |  |
| 8a | 44.8, CH_2_ | 2.70, m | H-9a, H-9b | C-6, C-7 |  |
| 8b |  | 2.70, m | H-9a, H-9b | C-6, C-7 |  |
| 9a | 18.9, CH_2_ | 1.64, m | H-8a, H-8b, H-10 |  |  |
| 9a |  | 1.64, m | H-8a, H-8b, H-10 |  |  |
| 10 | 14.2, CH_3_ | 0.93, t, (7.0) | H-9a, H-9b |  |  |
| 11 | 34.7, CH_3_ | 1.57, s |  | C-1, C-2, C-3 |  |
| 12 | 8.9, CH_3_ | 1.90, s |  | C-3, C-4, C-5 |  |
| 13 | 60.4, CH_3_ | 3.79 s |  | C-5 |  |

^a^ In chloroform-*d*. ^b^ Recorded at 125 MHz. ^c^Recorded at 500 MHz.

**Table S3.** 1D and 2D NMR data of aspergiflone C (**3**)

| **No.** | **3^a^** | | | | |
| --- | --- | --- | --- | --- | --- |
|  | ***δ*_C_^b^, type** | ***δ*_H_^c^ (*J* in Hz)** | **^1^H-^1^H COSY** | **HMBC** | **NOESY** |
| 1 | 150.1, C |  |  |  |  |
| 2 | 74.7, C |  |  |  |  |
| 3 | 200.0, C |  |  |  |  |
| 4 | 105.6, C |  |  |  |  |
| 5 | 169.7, C |  |  |  |  |
| 6 | 96.2, CH | 7.35, s |  | C-1, C-5 |  |
| 7 | 32.6, CH_3_ | 1.48, s |  | C-1, C-2, C-3 |  |
| 8 | 7.4, CH_3_ | 1.79, s |  | C-3, C-4, C-5 |  |
| 9 | 176.2, C |  |  |  |  |
| 10 | 44.3, CH | 2.33, m | H-11a, H-11b, H-13 |  |  |
| 11a | 27.4, CH_3_ | 1.53, m | H-10, H-12 |  |  |
| 11b |  | 1.73, m | H-10, H-12 |  |  |
| 12 | 11.9, CH_3_ | 0.95, t, (7.5) | H-11a, H-11b |  |  |
| 13 | 17.4, CH_3_ | 1.22, s | H-10 | C-9, C-10, C-11 |  |
| 14 | 56.5, CH_3_ | 3.93 s |  | C-5 |  |

^a^ In chloroform-*d*. ^b^ Recorded at 125 MHz. ^c^Recorded at 500 MHz.

**Table S4.** 1D and 2D NMR data of aspergiflone D (**4**)

| **No.** | **4^a^** | | | | |
| --- | --- | --- | --- | --- | --- |
|  | ***δ*_C_^b^, type** | ***δ*_H_^c^ (*J* in Hz)** | **^1^H-^1^H COSY** | **HMBC** | **NOESY** |
| 1 | 191.8, C |  |  |  |  |
| 2 | 81.5, C |  |  |  |  |
| 3 | 202.9, C |  |  |  |  |
| 4 | 73.7, C |  |  |  |  |
| 5 | 173.2, C |  |  |  |  |
| 6 | 100.1, CH | 5.57, s |  | C-1, C-5 |  |
| 7 | 24.4, CH_3_ | 1.70, s |  | C-1, C-2, C-3 |  |
| 8 | 23.6, CH_3_ | 1.65, s |  | C-3, C-4, C-5 |  |
| 9 | 173.1, C |  |  |  |  |
| 10a | 35.1, CH_2_ | 2.41, m | H-11a, H-11b | C-9 |  |
| 10b |  | 2.41, m | H-11a, H-11b | C-9 |  |
| 11a | 18.4, CH_3_ | 1.65, m | H-10a, H-10b, H-12 |  |  |
| 11b |  | 1.65 m | H-10a, H-10b, H-12 |  |  |
| 12 | 13.6, CH_3_ | 0.97, t, (7.0) | H-11a, H-11b |  |  |
| 13 | 57.1, CH_3_ | 3.88, s |  | C-1 |  |

^a^ In chloroform-*d*4. ^b^ Recorded at 125 MHz. ^c^Recorded at 500 MHz.

**Table S5**. Partial ^1^H NMR and ^13^C NMR data of compounds **1** and **7**

| No. | **1**^a^ | | No. | **7**^a^ | |
| --- | --- | --- | --- | --- | --- |
|  | *δ*_C_^b^, type | *δ*_H_^c^ (*J* in Hz) |  | *δ*_C_^b^, type | *δ*_H_^c^ (*J* in Hz) |
| 7' | 23.5, CH_3_ | 1.67, s | 7 | 24.2, CH_3_ | 1.65, s |
| 7 | 24.1, CH_3_ | 1.73, s | 7 | 24.2, CH_3_ | 1.65, s |
| 8 | 26.3, CH_3_ | 1.75, s | 8 | 23.5, CH_3_ | 1.69, s |
| 9 | 175.8, C |  | 9 | 176.0, C |  |
| 10 | 40.0, CH | 2.49, m | 10 | 40.0, CH | 2.49, m |
| 11a | 26.9, CH_2_ | 1.70, m | 11a | 26.8, CH_2_ | 1.72, m |
| 11a |  | 1.50, m | 11b |  | 1.50, m |
| 12 | 11.5, CH_3_ | 0.95, m | 12 | 11.5, CH_3_ | 0.95, m |
| 13 | 16.4, CH_3_ | 1.16, t, (7.0) | 13 | 16.3, CH_3_ | 1.16, t, (7.0) |
| 9' | 175.8, C |  | 9 | 176.0, C |  |
| 10' | 40.1, CH | 2.49, m | 10 | 40.0, CH | 2.49, m |
| 11'a | 26.8, CH_2_ | 1.70, m | 11a | 26.8, CH_2_ | 1.72, m |
| 11'b |  | 1.50, m | 11b |  | 1.50, m |
| 12' | 11.5, CH_3_ | 0.95, m | 12 | 11.5, CH_3_ | 0.95, m |
| 13' | 16.3, CH_3_ | 1.16, t, (7.0) | 13 | 16.3, CH_3_ | 1.16, t, (7.0) |

^a^ In chloroform-*d*. ^b^ Recorded at 125 MHz. ^c^Recorded at 500 MHz.

1. The determination of relative and absolute configuration for compounds 1‒4

4.1 Conformational search

Conformational search of all [possible](javascript:;) configurations were carried out by MacroModel integrated in Maestro V11.9 (Schrödinger Inc.).^1^ The OPLS3e force field^2^ and an energy below a threshold of 10 kJ mol^-1^ were employed.^3^ Eliminating redundant conformer used root-mean-squared-distance (RMSD) cutoff of 0.5 Å and the maximum iterations was 2500. After energy minimization, the [unstable](javascript:;) configurations were excluded.

4.2 Quantum chemical NMR calculation

In order to establish the relative configuration of molecules **1**‒**4**, ^13^C NMR chemical shifts were calculated by Gaussian 09 program package.^4^ Excluding unstable conformers by conformational search, the remaining conformers were optimized with the density functional theory (DFT) at the B3LYP/6-31G (d, p) level,^5^ and all minima displayed no imaginary frequencies by vibrational frequency analysis at the same level. The populations of conformers were calculated according to the Boltzmann distribution theory and their relative Gibbs free energy. GIAO calculations of NMR shielding were accomplished for all stable conformations by DFT GIAO model at PCM/mPW1PW91/6-31+G**^6^ level for CP3 calculations and DP4+ calculations. The qccNMR results were shown in the following Tables S6‒S8 and Figures S2‒S3.

**Table S6. Experimental NMR data and calculated NMR data for compound 1**

| Nuclei | SP^2^ | Experiment **1** | Calculation | |
| --- | --- | --- | --- | --- |
|  |  |  | Conf.1 (2*R*4*R*8*R*10*R*19*S*24*S)* | Conf.2 (2*R*4*R*8*S*10*S*19*S*24*R*) |
| C | X | 205.0 | 196.9 | 197.29 |
| C | X | 204.7 | 191.8 | 192.72 |
| C | X | 192.9 | 177.6 | 177.17 |
| C | X | 191.9 | 176.7 | 176.63 |
| C | X | 177.9 | 164.8 | 164.53 |
| C | X | 175.8 | 163.8 | 164.11 |
| C | X | 175.8 | 163.2 | 162.97 |
| C | X | 168.5 | 158.8 | 156.84 |
| C | X | 115.6 | 102.8 | 105.49 |
| C | X | 99.1 | 89.4 | 90.43 |
| C |  | 82.0 | 77.7 | 77.74 |
| C |  | 80.7 | 74.1 | 76.08 |
| C |  | 75.4 | 72.0 | 72.40 |
| C |  | 60.5 | 53.0 | 53.68 |
| C |  | 53.0 | 52.0 | 49.93 |
| C |  | 56.7 | 48.5 | 49.54 |
| C |  | 40.0 | 37.8 | 38.06 |
| C |  | 40.1 | 37.1 | 36.39 |
| C |  | 31.4 | 28.3 | 26.46 |
| C |  | 26.3 | 26.9 | 25.03 |
| C |  | 26.9 | 26.2 | 24.36 |
| C |  | 26.8 | 24.8 | 23.73 |
| C |  | 24.1 | 21.8 | 22.03 |
| C |  | 23.5 | 20.9 | 20.57 |
| C |  | 21.3 | 20.4 | 18.41 |
| C |  | 16.3 | 15.1 | 15.35 |
| C |  | 16.4 | 13.7 | 10.09 |
| C |  | 11.5 | 8.8 | 9.16 |
| C |  | 11.5 | 8.4 | 7.41 |
| H | X | 5.54 | 6.01 | 6.64 |
| H |  | 4.13 | 4.90 | 5.58 |
| H |  | 4.13 | 4.86 | 5.14 |
| H |  | 4.13 | 4.66 | 5.10 |
| H |  | 3.74 | 4.57 | 5.08 |
| H |  | 3.74 | 4.14 | 4.75 |
| H |  | 3.74 | 4.10 | 4.73 |
| H |  | 2.69 | 3.58 | 4.04 |
| H |  | 3.27 | 2.88 | 3.77 |
| H |  | 1.75 | 2.81 | 3.52 |
| H |  | 2.49 | 2.73 | 3.39 |
| H |  | 2.49 | 2.61 | 3.11 |
| H |  | 1.67 | 2.29 | 2.91 |
| H |  | 1.75 | 2.19 | 2.81 |
| H |  | 1.7 | 2.06 | 2.78 |
| H |  | 1.67 | 2.01 | 2.74 |
| H |  | 1.37 | 1.98 | 2.70 |
| H |  | 1.7 | 1.95 | 2.68 |
| H |  | 1.75 | 1.91 | 2.65 |
| H |  | 1.73 | 1.89 | 2.64 |
| H |  | 1.37 | 1.87 | 2.61 |
| H |  | 1.67 | 1.85 | 2.59 |
| H |  | 1.73 | 1.83 | 2.57 |
| H |  | 1.5 | 1.80 | 2.54 |
| H |  | 1.16 | 1.75 | 2.50 |
| H |  | 1.16 | 1.69 | 2.42 |
| H |  | 1.16 | 1.66 | 2.38 |
| H |  | 1.73 | 1.61 | 2.29 |
| H |  | 0.95 | 1.57 | 2.25 |
| H |  | 1.37 | 1.53 | 2.17 |
| H |  | 1.5 | 1.49 | 2.12 |
| H |  | 1.16 | 1.45 | 2.08 |
| H |  | 1.16 | 1.37 | 1.98 |
| H |  | 0.95 | 1.29 | 1.93 |
| H |  | 1.16 | 1.25 | 1.89 |
| H |  | 0.95 | 1.17 | 1.82 |
| H |  | 0.95 | 1.14 | 1.77 |
| H |  | 0.95 | 1.10 | 1.73 |
| H |  | 0.95 | 1.06 | 1.62 |


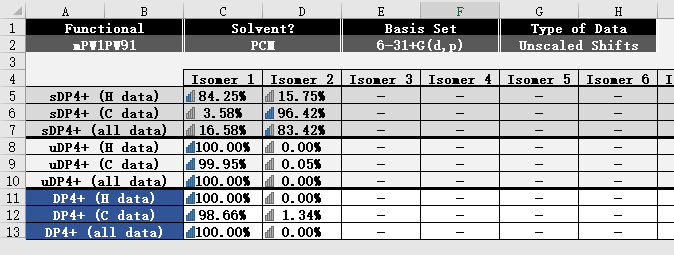


**Figure S2**. The DP4+ results between calculated and experimental NMR data for **1**

**Table S7. Experimental NMR data and calculated NMR data for compound 3**

| Nuclei | SP^2^ | Experiment **3** | Calculation | |
| --- | --- | --- | --- | --- |
|  |  |  | Conf.1 (2*R*10*R)* | Conf.2 (2*R*10*S*) |
| C | X | 200 | 182.79 | 182.86 |
| C | X | 176.2 | 163.31 | 164.30 |
| C | X | 150.1 | 154.79 | 155.71 |
| C | X | 169.7 | 153.67 | 154.51 |
| C | X | 105.6 | 99.91 | 102.65 |
| C | X | 96.2 | 93.73 | 96.72 |
| C |  | 74.7 | 57.30 | 61.51 |
| C |  | 56.5 | 48.07 | 52.71 |
| C |  | 44.3 | 39.31 | 43.77 |
| C |  | 32.6 | 25.97 | 31.71 |
| C |  | 27.4 | 23.48 | 29.35 |
| C |  | 17.4 | 15.41 | 19.22 |
| C |  | 11.9 | 8.55 | 14.16 |
| C |  | 7.4 | 4.15 | 10.12 |
| H | X | 7.35 | 8.86 | 8.54 |
| H |  | 3.93 | 5.18 | 5.64 |
| H |  | 3.93 | 5.92 | 5.35 |
| H |  | 3.93 | 5.89 | 5.31 |
| H |  | 2.33 | 4.61 | 4.11 |
| H |  | 1.79 | 4.55 | 3.97 |
| H |  | 1.53 | 3.97 | 3.61 |
| H |  | 1.22 | 3.80 | 3.22 |
| H |  | 1.79 | 3.75 | 3.12 |
| H |  | 1.48 | 3.64 | 3.03 |
| H |  | 1.79 | 3.60 | 2.99 |
| H |  | 1.48 | 3.51 | 2.97 |
| H |  | 1.73 | 3.42 | 2.86 |
| H |  | 1.22 | 3.36 | 2.79 |
| H |  | 1.48 | 3.29 | 2.69 |
| H |  | 1.22 | 3.25 | 2.60 |
| H |  | 0.95 | 3.14 | 2.57 |
| H |  | 0.95 | 3.11 | 2.52 |
| H |  | 0.95 | 2.94 | 2.31 |


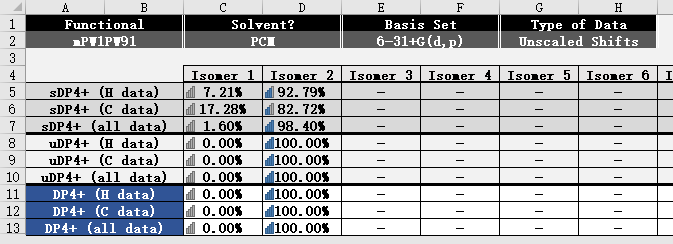


**Figure S3**. The DP4+ results between calculated and experimental NMR data for **3**

**Table S8. Experimental NMR data and calculated NMR data for compound 4**

| Nuclei | SP^2^ | Experiment **4** | Calculation | |
| --- | --- | --- | --- | --- |
|  |  |  | Conf.1 (2*R*4*R)* | Conf.2 (2*R*4*S*) |
| C | X | 202.9 | 190.4 | 197.4 |
| C | X | 191.8 | 175.9 | 175.4 |
| C | X | 173.2 | 160.4 | 161.0 |
| C | X | 173.1 | 158.4 | 158.8 |
| C | X | 100.1 | 92.7 | 91.8 |
| C |  | 81.5 | 82.5 | 82.8 |
| C |  | 73.7 | 69.7 | 72.4 |
| C |  | 57.1 | 49.7 | 51.2 |
| C |  | 35.1 | 31.2 | 32.7 |
| C |  | 23.6 | 25.0 | 25.4 |
| C |  | 18.4 | 16.5 | 23.7 |
| C |  | 24.4 | 16.1 | 18.4 |
| C |  | 13.6 | 9.9 | 10.4 |
| H | X | 5.57 | 6.10 | 6.30 |
| H |  | 3.88 | 4.67 | 5.04 |
| H |  | 3.88 | 4.10 | 4.45 |
| H |  | 3.88 | 4.08 | 4.42 |
| H |  | 1.65 | 2.70 | 4.41 |
| H |  | 1.70 | 2.46 | 2.83 |
| H |  | 2.41 | 2.38 | 2.63 |
| H |  | 1.65 | 2.26 | 2.56 |
| H |  | 1.70 | 2.21 | 2.56 |
| H |  | 1.65 | 2.13 | 2.40 |
| H |  | 1.65 | 1.96 | 2.21 |
| H |  | 1.65 | 1.57 | 2.01 |
| H |  | 2.41 | 1.50 | 1.91 |
| H |  | 1.70 | 1.38 | 1.84 |
| H |  | 0.97 | 1.32 | 1.63 |
| H |  | 0.97 | 1.20 | 1.52 |
| H |  | 0.97 | 0.73 | 1.06 |


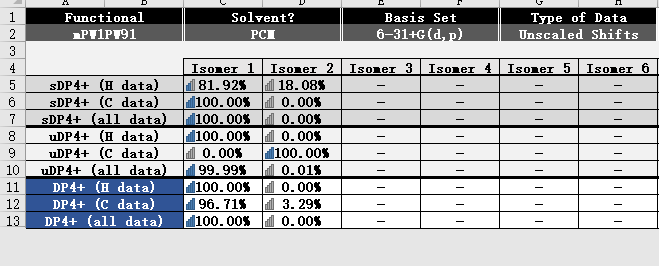


**Figure S4**. The DP4+ results between calculated and experimental NMR data for **4**

4.3 Elucidation of absolute configuration by TDDFT-ECD

To determine absolute configurations of compounds **1**–**4**, and **7**, the spin-allowed excitation energies and rotatory (Rn) and oscillator strengths (fn) of the lowest excited states of stable conformers were calculated for ECD spectra using TD-DFT method at the CAM-B3LYP/6-311G(d,p) level^7^ with IEFPCM solvent model for methanol in agreement with the experiment condition. All the calculations in this article were performed using the Gaussian 10. Electronic transitions were expanded as Gaussian curves with a FQHM (full width at half maximum) for each peak of 0.40 eV. The ECD spectra were combined after Boltzmann weighting according to their population contribution.

1. Plausible biosynthetic pathway of 1

Aspergiflone A (**1**) represented a new carbon skeleton in the family of cyclohexenone, which were considered to origin the precursor, phomaligol A (**7**), a known co-isolate.^8^ Firstly, the **7** under acetylation to form acetylphomaligol A (**6**), a known co-isolate.^8, 9^ **7** underwent dehydroxylation to form phomaligadione A, a known cyclohexanone previously isolated from fungus *Phoma lingam.*^10, 11^ Phomaligadione A underwent the catalyzed reaction of methyltransferases to form the key intermediate **7a.**^12^ Finally, **7a** and **7** underwent polymerization to yield aspergiflone A (**1**) shown in Scheme 1.^13, 14^

**Scheme 1.** Proposed biosynthetic pathways of compounds **1**, **6**, **7**

1. Hydrolysis of 3 for determination of absolute conﬁguration

Compound **3** was dissolved in 6N HCl (0.6 mL) and heated to 100 °C for 1 h. The solution was cooled and extracted with EtOAc twice. The EtOAc layer was concentrated under reduced pressure.^15^ The EtOAc extract was separated by semi-preparative HPLC (ODS, 5 µm, 250 × 4.6 mm; CH_3_OH/H_2_O, 70:30, *v/v*; 1.0 mL/min).


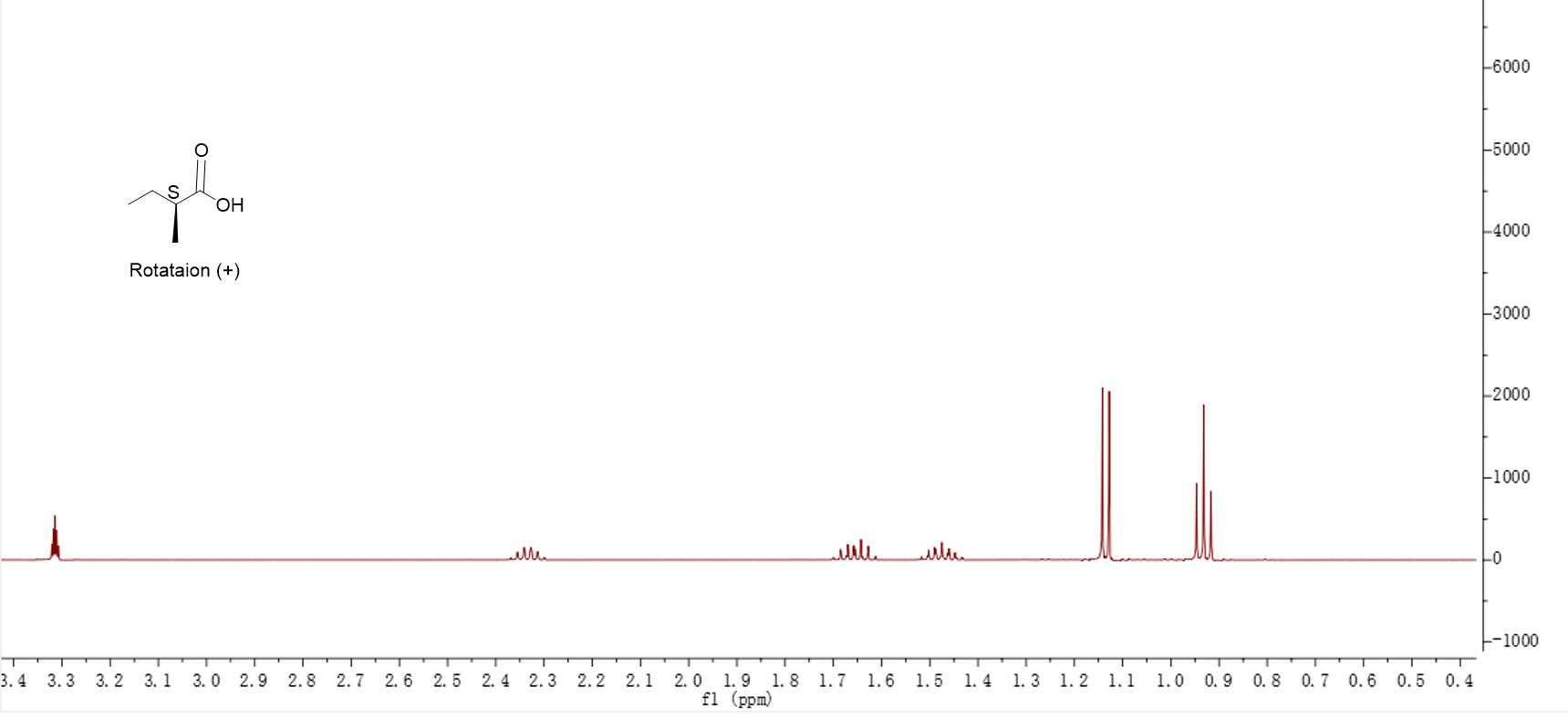


**Figure S5.** ^1^H NMR spectrum of (+) 2-methylbutanoic acid

1. Reference
2. Schrödinger Release 2019-1: MacroModel, Schrödinger, LLC, New York, NY, 2019.

2.Roos, K.; Wu, C.; Damm, W.; Reboul, M.; Stevenson, J. M.; Lu, C.; Dahlgren, M. K.; Mondal, S.; Chen, W.; Wang, L.; Abel, R.; Friesner, R. A.; Harder, E. D., OPLS3e: Extending Force Field Coverage for Drug-Like Small Molecules. *J Chem Theory Comput* **2019,** *15*, 1863-1874.

3 Smith, S. G.; Goodman, J. M., Assigning Stereochemistry to Single Diastereoisomers by GIAO NMR Calculation: The DP4 Probability. *J. Am. Chem. Soc.* **2010,** *132*, 12946-12959.

4. M. Frisch, G. Trucks, H. Schlegel, G. Scuseria, G. M. Robb, J. Cheeseman, G. Scalmani, V. Barone, G. Petersson, H. Nakatsuji, H. Gaussian, Inc. Wallingford, CT, **2016.**

5. Su, L. H.; Geng, C. A.; Li, T. Z.; Ma, Y. B.; Huang, X. Y.; Zhang, X. M.; Chen, J. J., Artatrovirenols A and B: Two Cagelike Sesquiterpenoids from Artemisia atrovirens. *J. Org. Chem.* **2020,** *85*, 13466-13471.

6. Li, S. W.; Cuadrado, C.; Yao, L. G.; Daranas, A. H.; Guo, Y. W., Quantum Mechanical-NMR-Aided Configuration and Conformation of Two Unreported Macrocycles Isolated from the Soft Coral Lobophytum sp.: Energy Calculations versus Coupling Constants. *Org. Lett.* **2020,** *22*, 4093-4096.

7. Suramitr, S.; Piriyagagoon, A.; Wolschann, P.; Hannongbua, S., Theoretical study on the structures and electronic properties of oligo(p-phenylenevinylene) carboxylic acid and its derivatives: effects of spacer and anchor groups. *Theor. Chem. Acc.* **2012,** *131*, 1-15.

8. Elbandy, M.; Shinde, P. B.; Hong, J.; Bae, K. S.; Kim, M. A.; Lee, S. M.; Jung, J. H., α-Pyrones and Yellow Pigments from the Sponge-Derived Fungus *Paecilomyces lilacinus*. *Bull. Korean Chem. Soc* **2009,** *30*, 188-192.

9. Vetting, M. W.; Yu, M.; Rendle, P. M.; Blanchard, J. S., The Substrate-induced Conformational Change of Mycobacterium tuberculosis Mycothiol Synthase. *J. Biol. Chem.* **2006,** *281*, 2795-2802.

10. Funabashi, M.; Grove, T. L.; Wang, M.; Varma, Y.; McFadden, M. E.; Brown, L. C.; Guo, C.; Higginbottom, S.; Almo, S. C.; Fischbach, M. A., A metabolic pathway for bile acid dehydroxylation by the gut microbiome. *Nature* **2020,** *582*, 566-570.

11. Pedras, M. S. C.; Morales, V. M.; Taylor, J. L., Phomaligols and phomaligadiones: new metabolites from the blackleg fungus. *Tetrahedron* **1993,** *49*, 8317.

12. Abdelraheem, E.; Thair, B.; Varela, R. F.; Jockmann, E.; Popadić, D.; Hailes, H. C.; Ward, J. M.; Iribarren, A. M.; Lewkowicz, E. S.; Andexer, J. N.; Hagedoorn, P. L.; Hanefeld, U., Methyltransferases: Functions and Applications. *ChemBioChem* **2022,** *23,* e202200212.

13. Shende, V. V.; Khatri, Y.; Newmister, S. A.; Sanders, J. N.; Lindovska, P.; Yu, F.; Doyon, T. J.; Kim, J.; Houk, K. N.; Movassaghi, M.; Sherman, D. H., Structure and Function of NzeB, a Versatile C–C and C–N Bond-Forming Diketopiperazine Dimerase. *J. Am. Chem. Soc.* **2020,** *142*, 17413-17424.

14. Christoff, R. M.; Al Bayer, M.; Soares da Costa, T. P.; Perugini, M. A.; Abbott, B. M., Enhancing allosteric inhibition of dihydrodipicolinate synthase through the design and synthesis of novel dimeric compounds. *RSC Med. Chem.* **2023,** *14*, 1698-1703.

1. Computational details


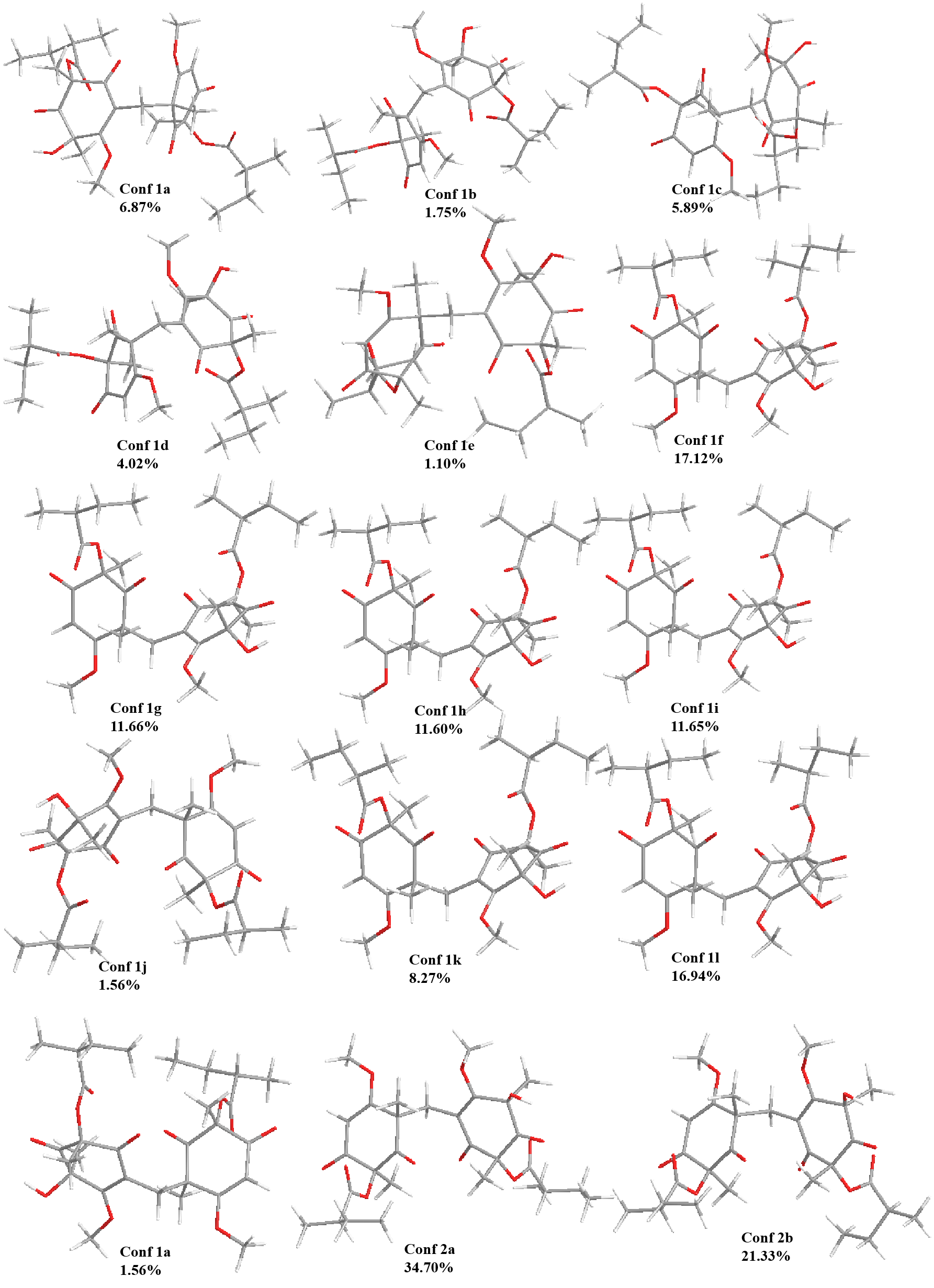


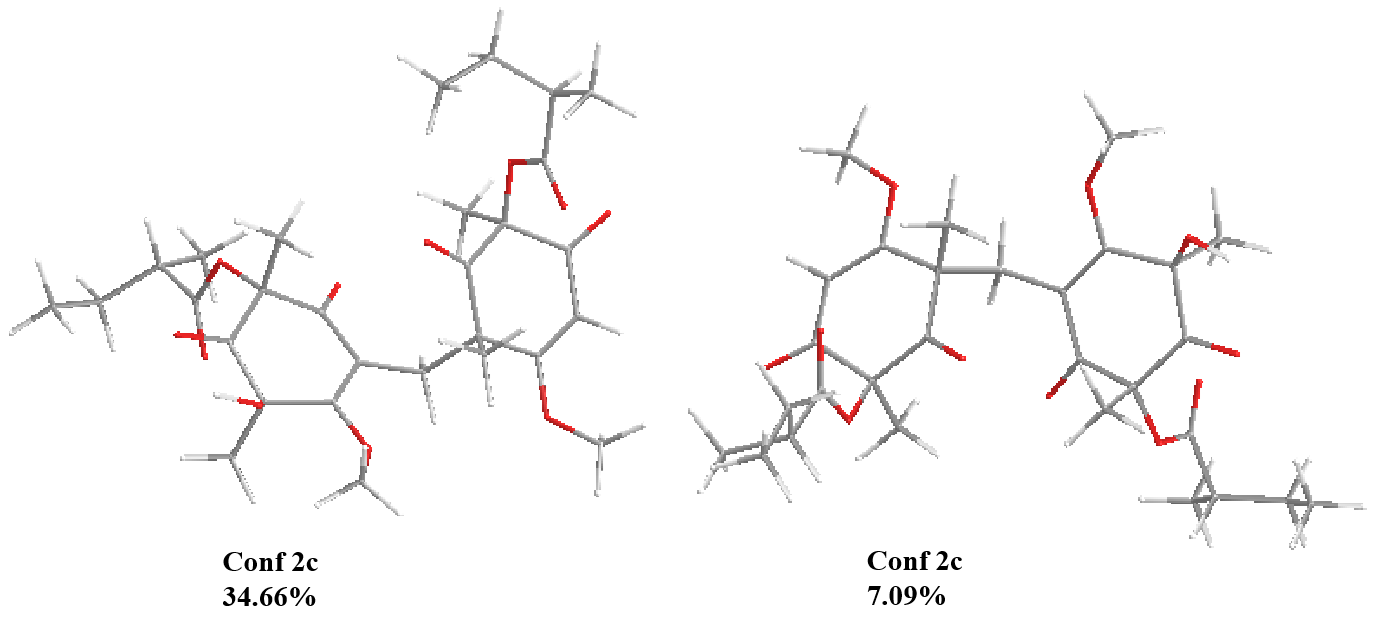


**Figure S6.** Stable conformers of compound **1** for two [relative](javascript:;) configurations


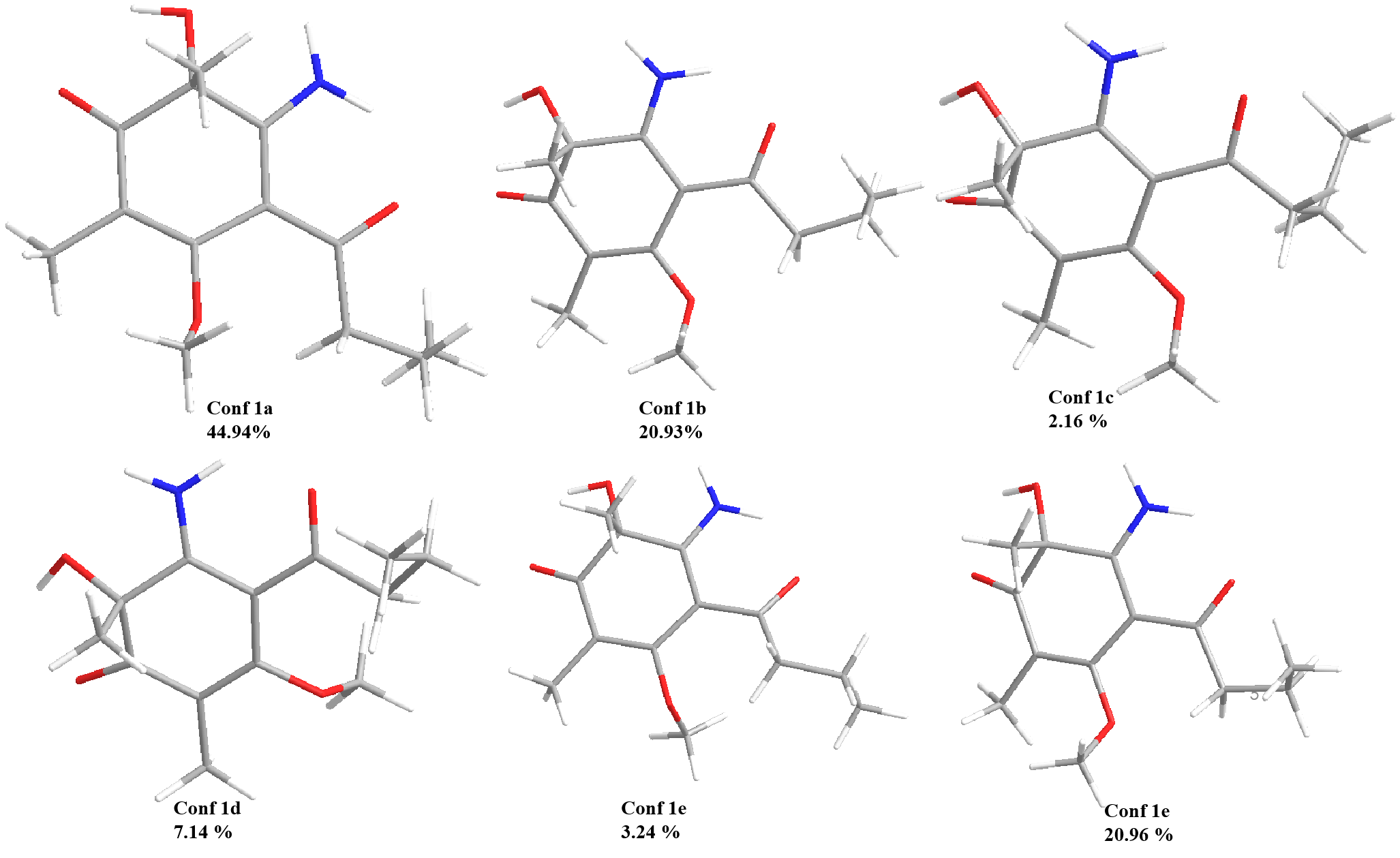


**Figure S7.** Stable conformers of compound **2** for [relative](javascript:;) configuration


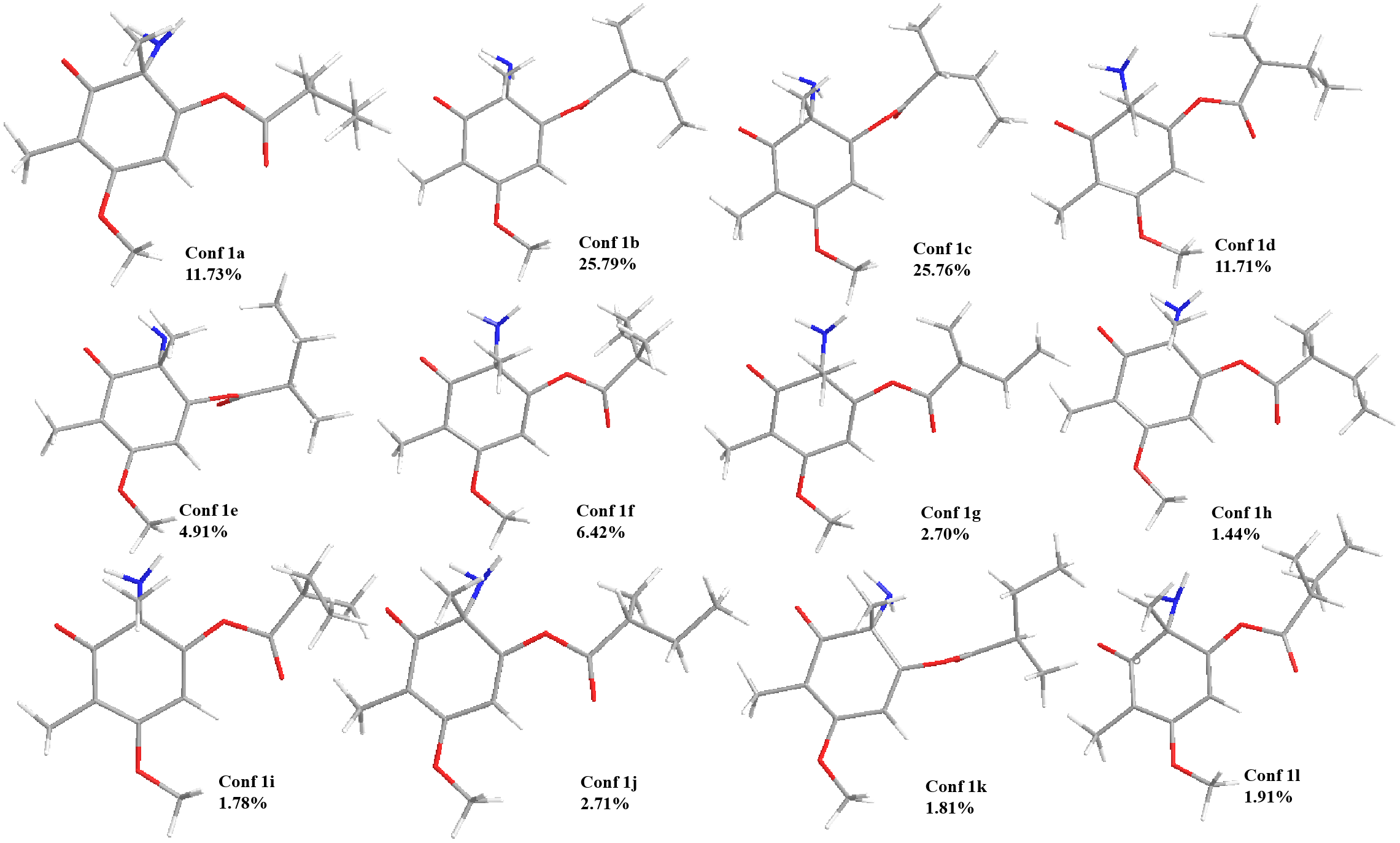
**
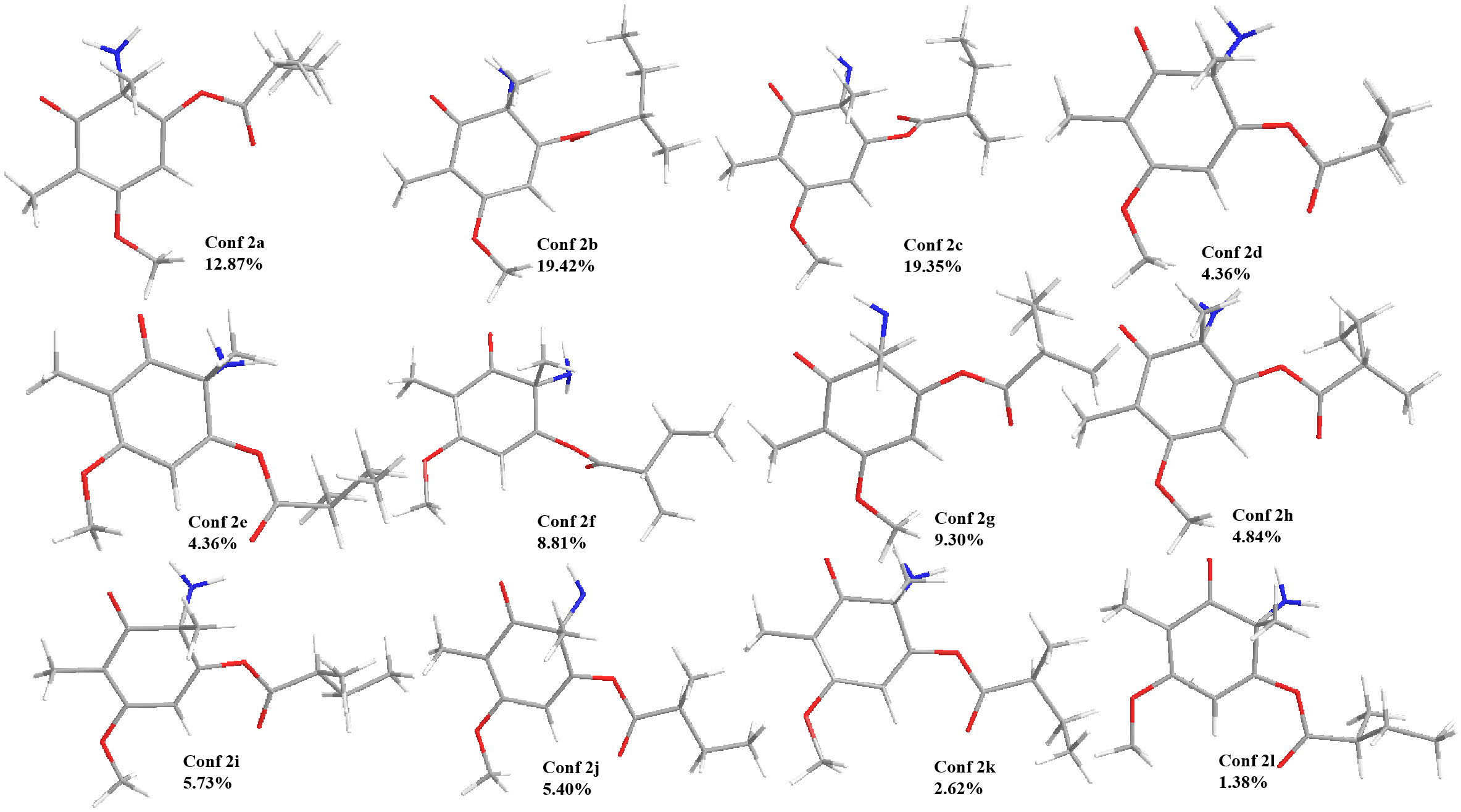
**

**Figure S8.** Stable conformers of compound **3** for two [relative](javascript:;) configurations


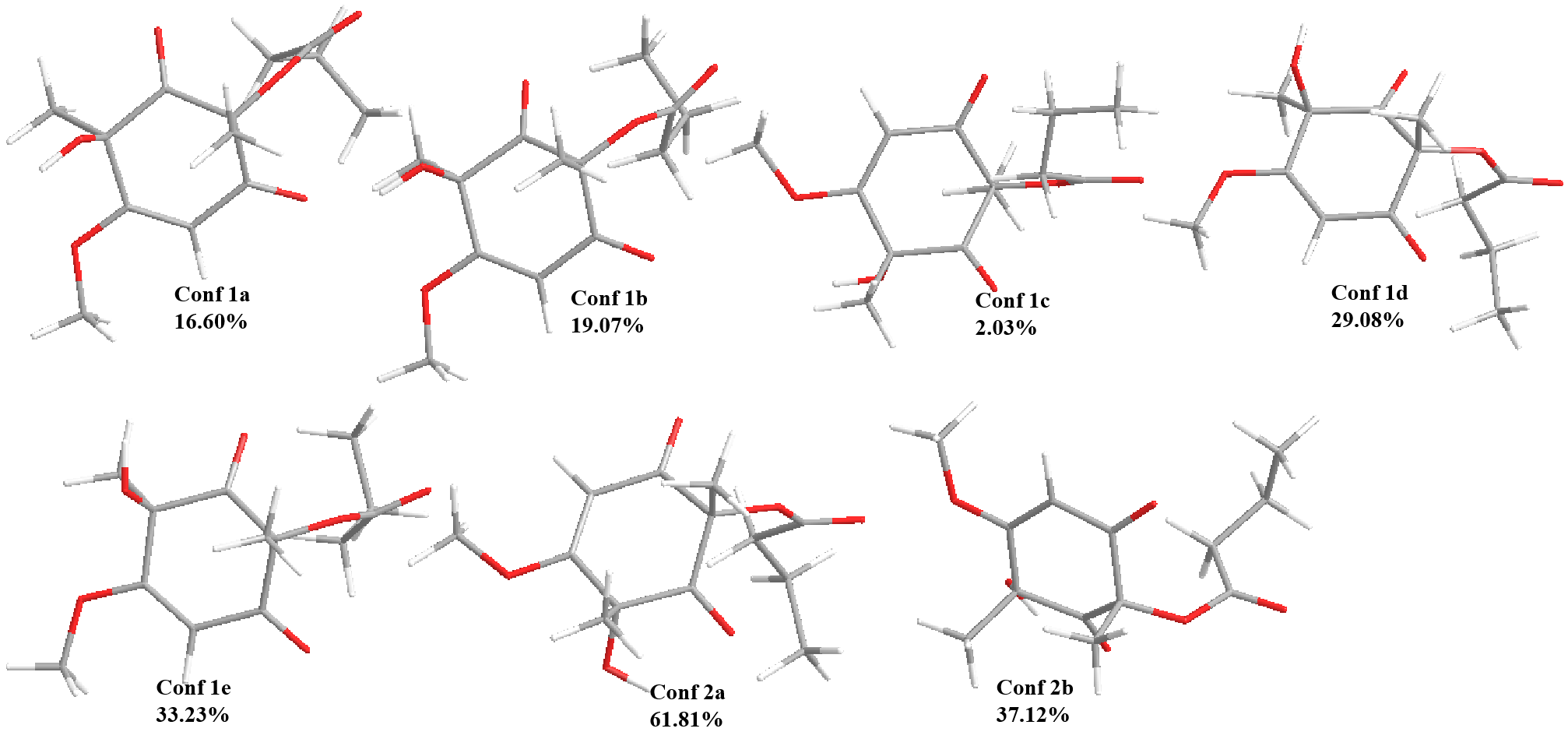


**Figure S9.** Stable conformers of compound **4** for two [relative](javascript:;) configurations


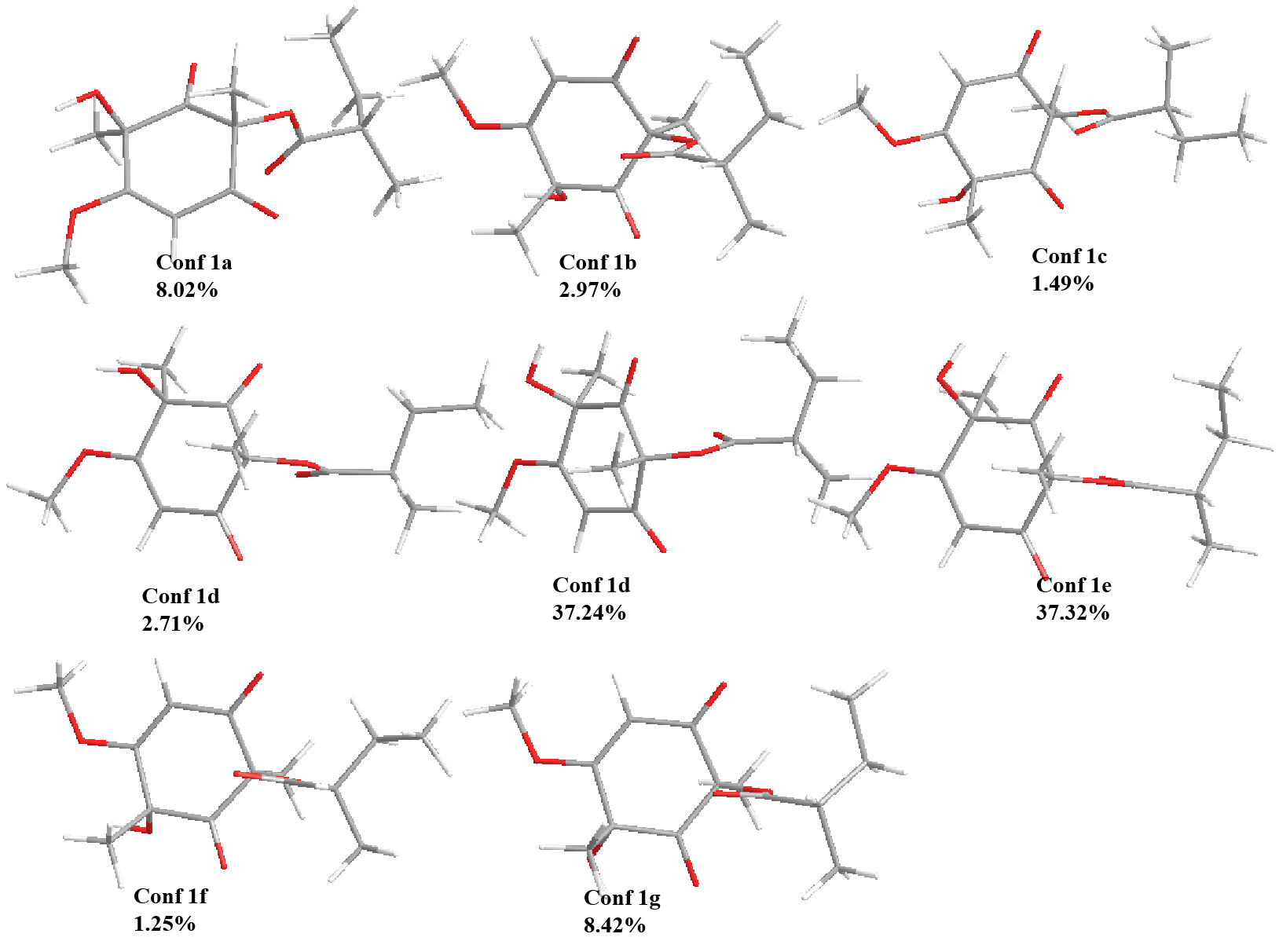


**Figure S10.** Stable conformers of compound **7** for [relative](javascript:;) configuration

**Table S9.** Optimized Z-Matrixes of compounds **1**–**4** and **7** in the Gas Phase (Å) at B3LYP/6-31G (d, p) level

1. The 1D and 2D NMR spectra of 1–4

**Figure S11.** The positive HRESIMS spectrum of compound **1**


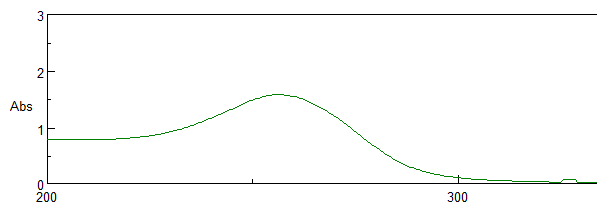


**Figure S12.** UV spectrum of compound **1**

**
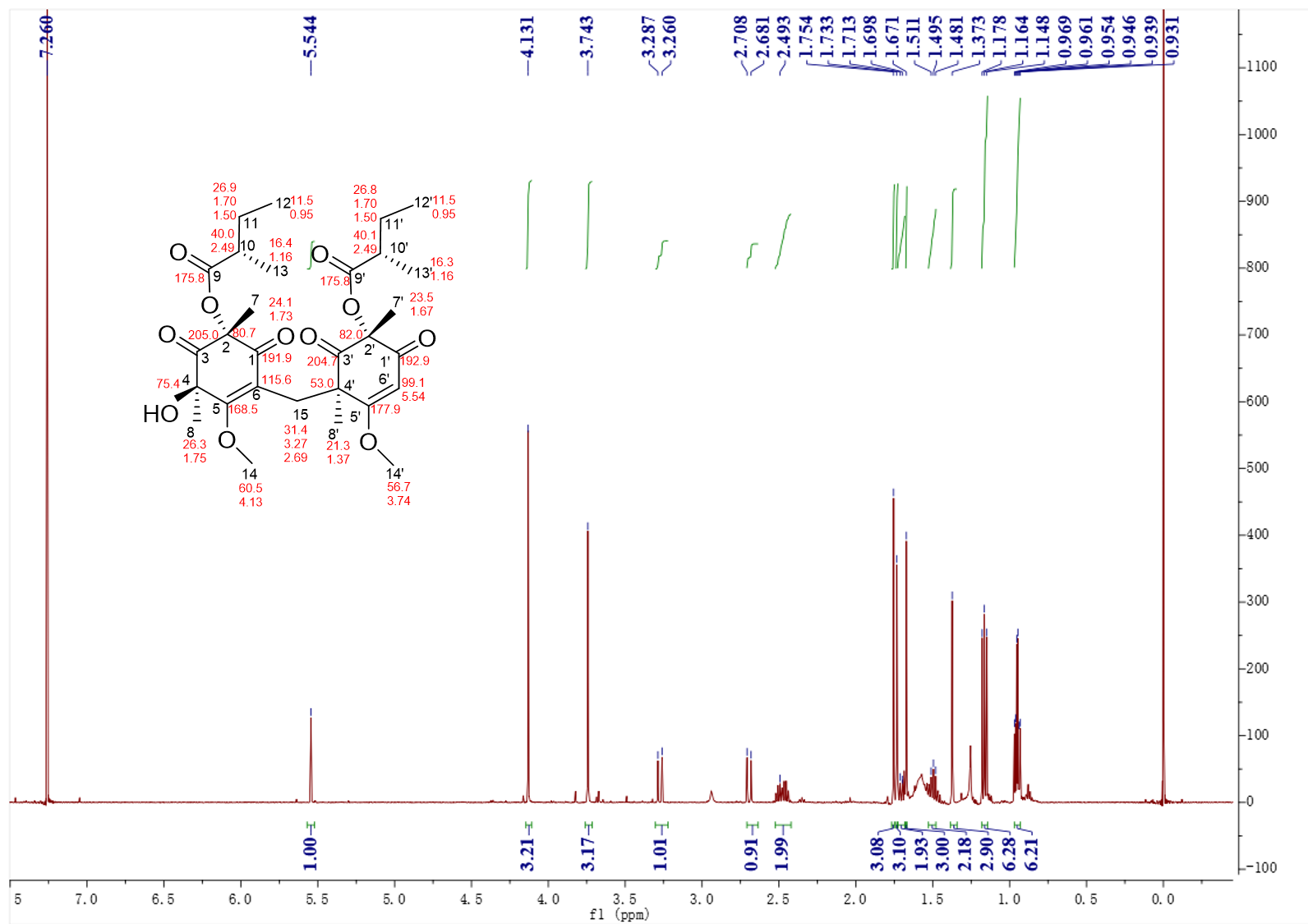
**

**Figure S13.** ^1^H NMR spectrum (500 MHz, CDCl_3_) of compound **1**


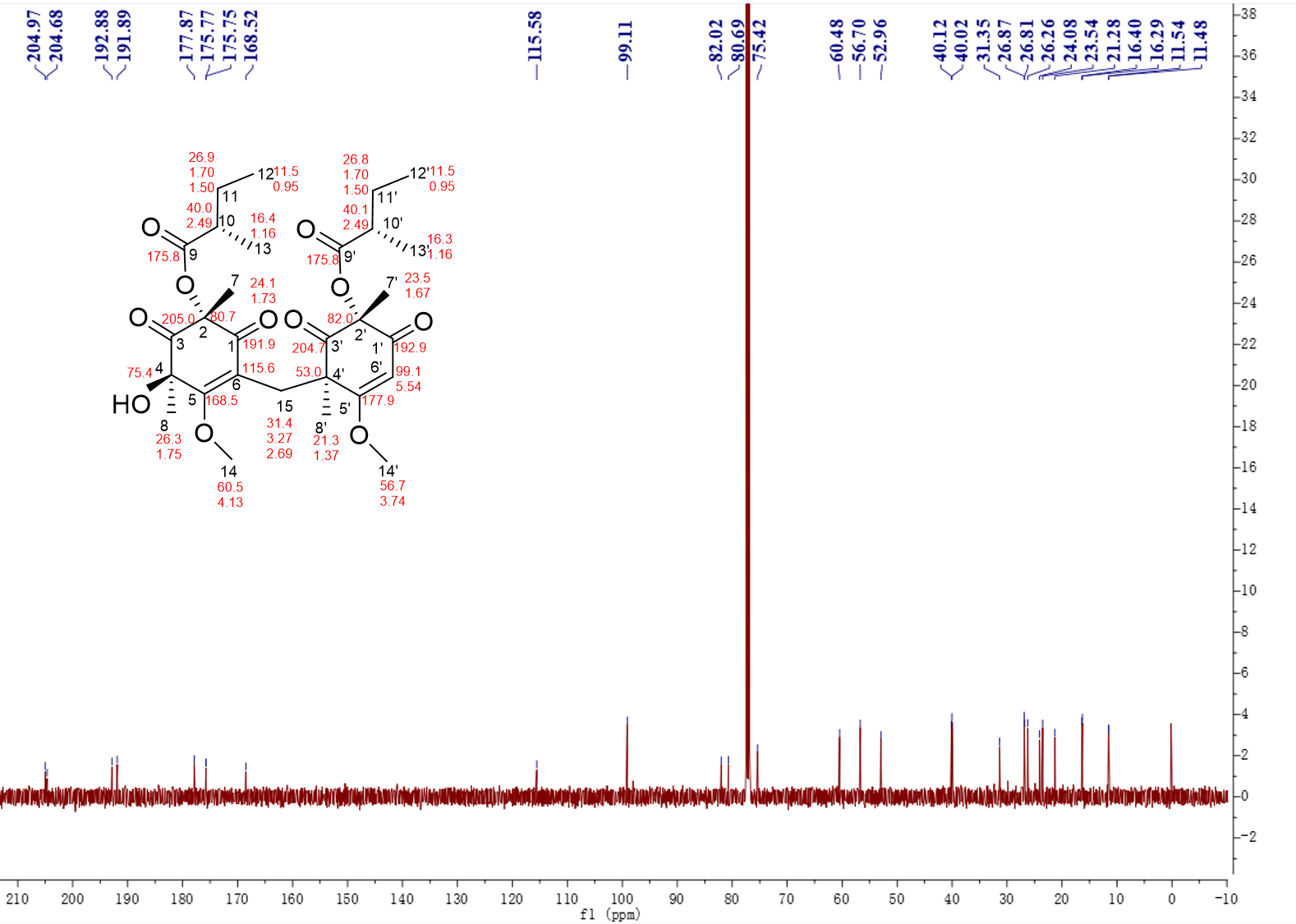


**Figure S14.** ^13^C NMR spectrum (125 MHz, CDCl_3_) of compound **1**


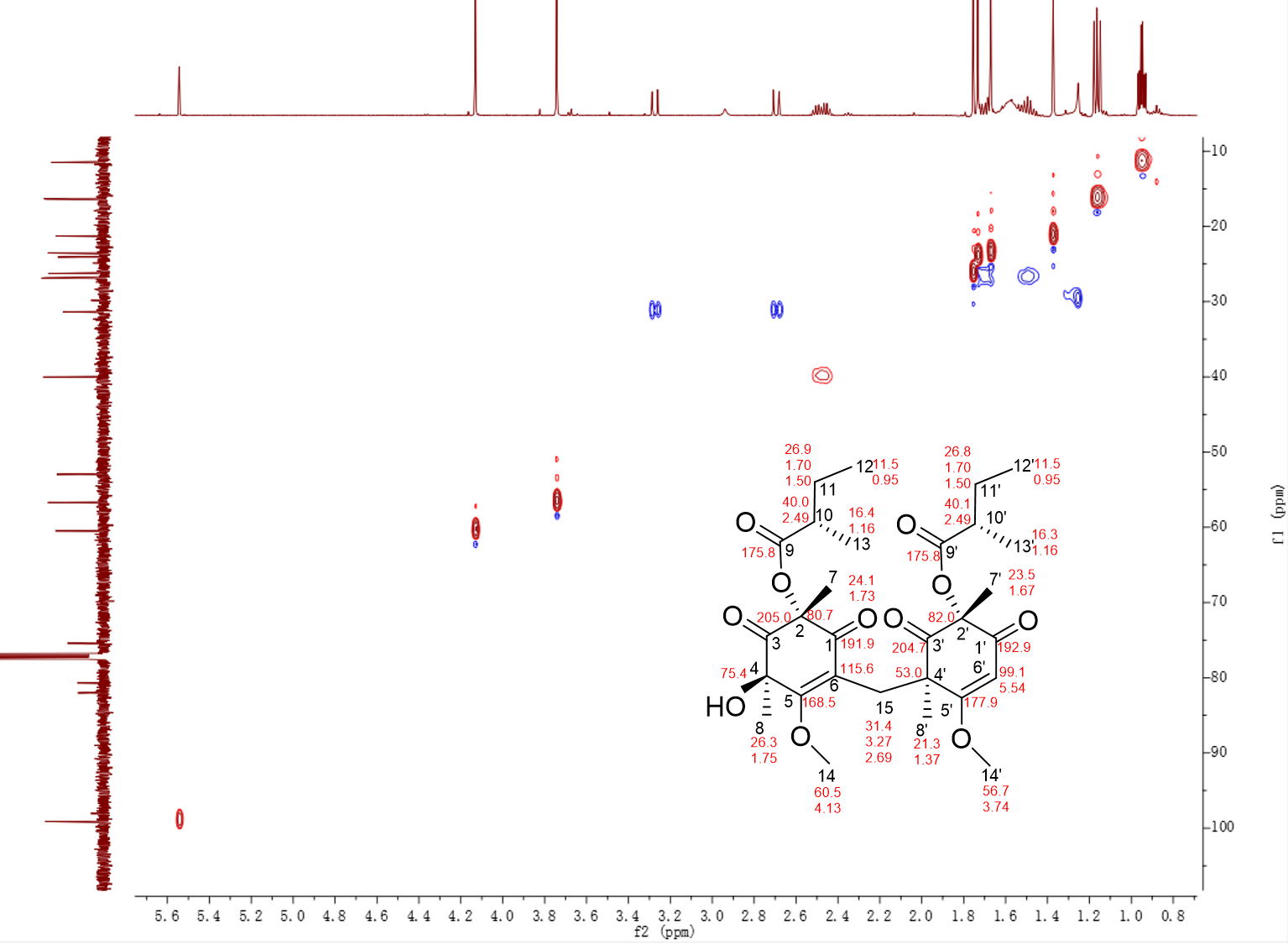


**Figure S15.** HSQC (500 MHz, CDCl_3_) of compound **1**


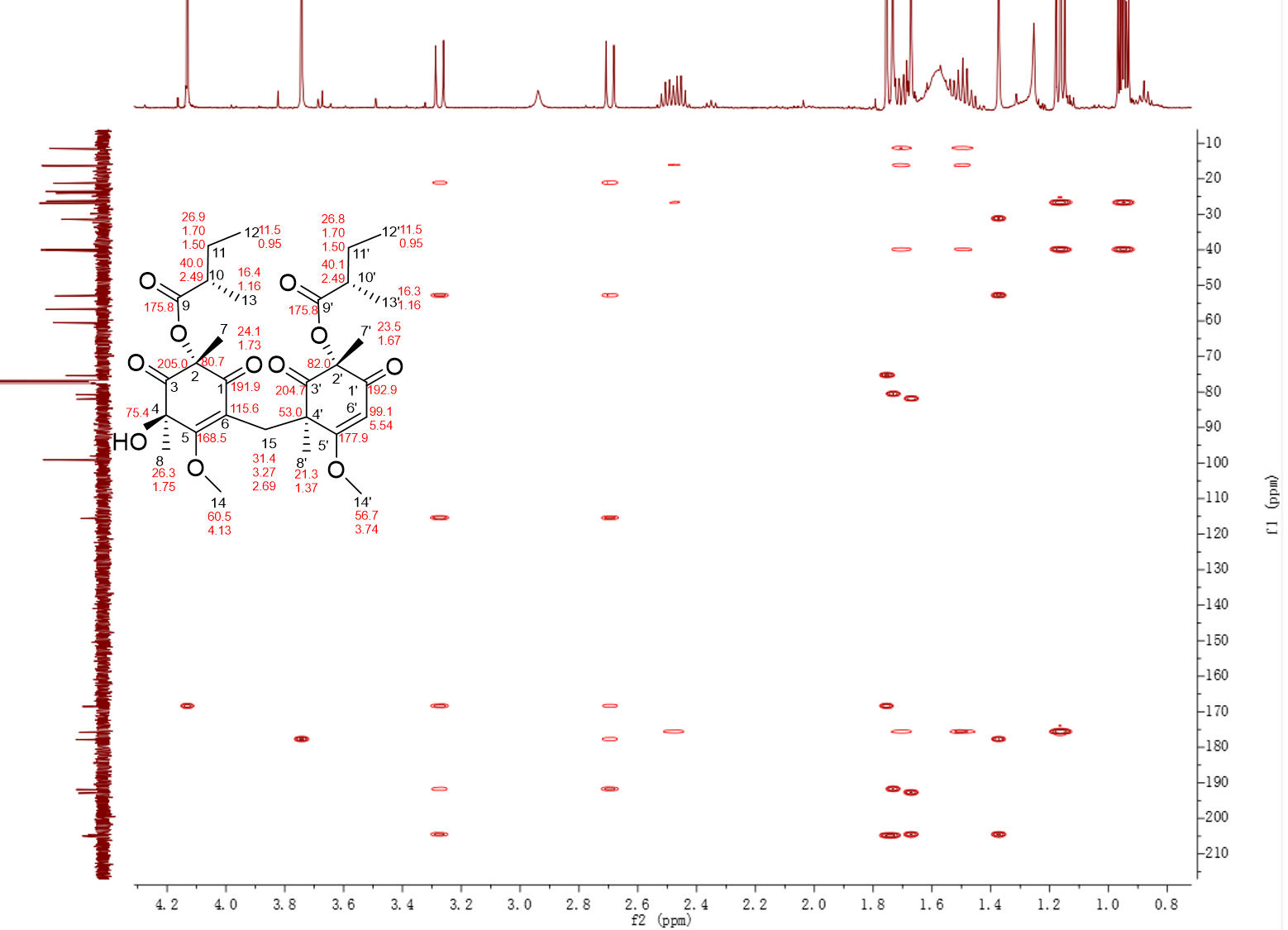


**Figure S16.** HMBC (125 MHz, CDCl_3_) of compound **1**


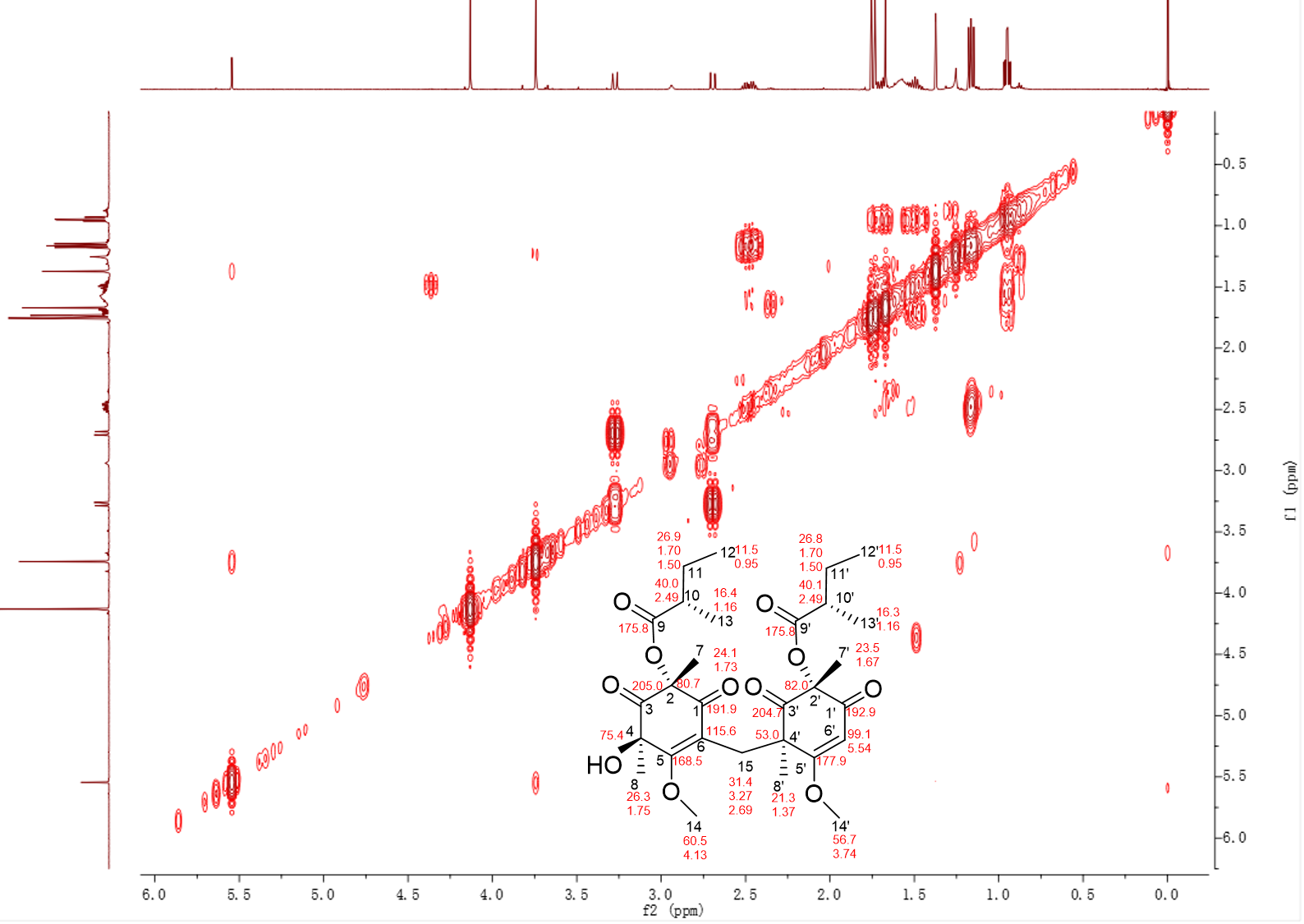


**Figure S17.** ^1^H-^1^H COSY (500 MHz, CDCl_3_) of compound **1**


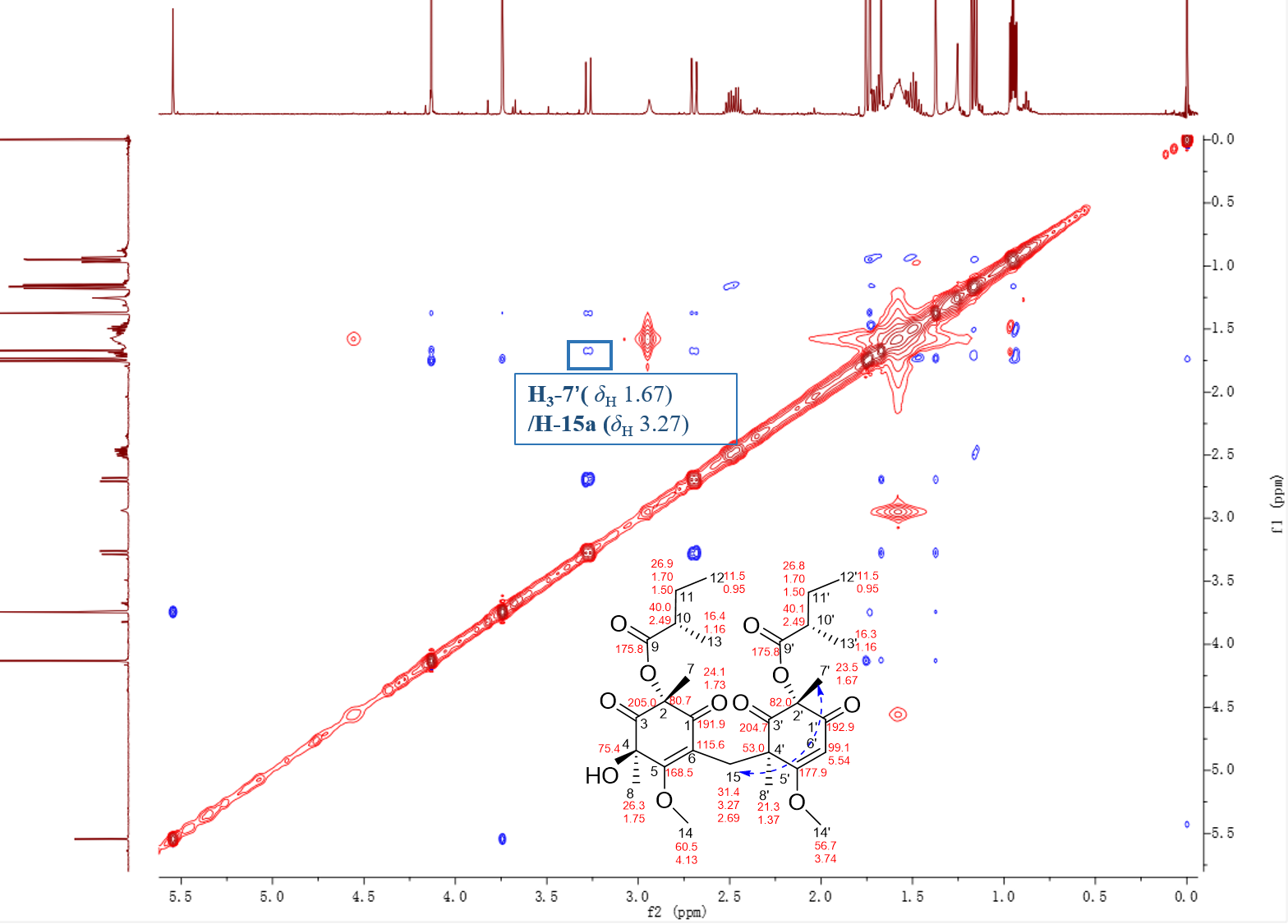


**Figure S18.** NOESY (500 MHz, CDCl_3_) of compound **1**

**Figure S19.** The positive HRESIMS spectrum of compound **2**


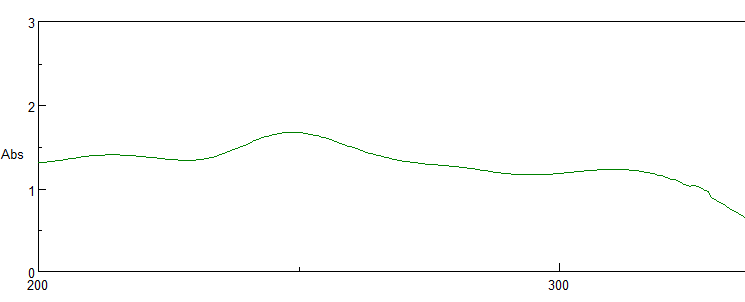


**Figure S20.** UV spectrum of compound **2**


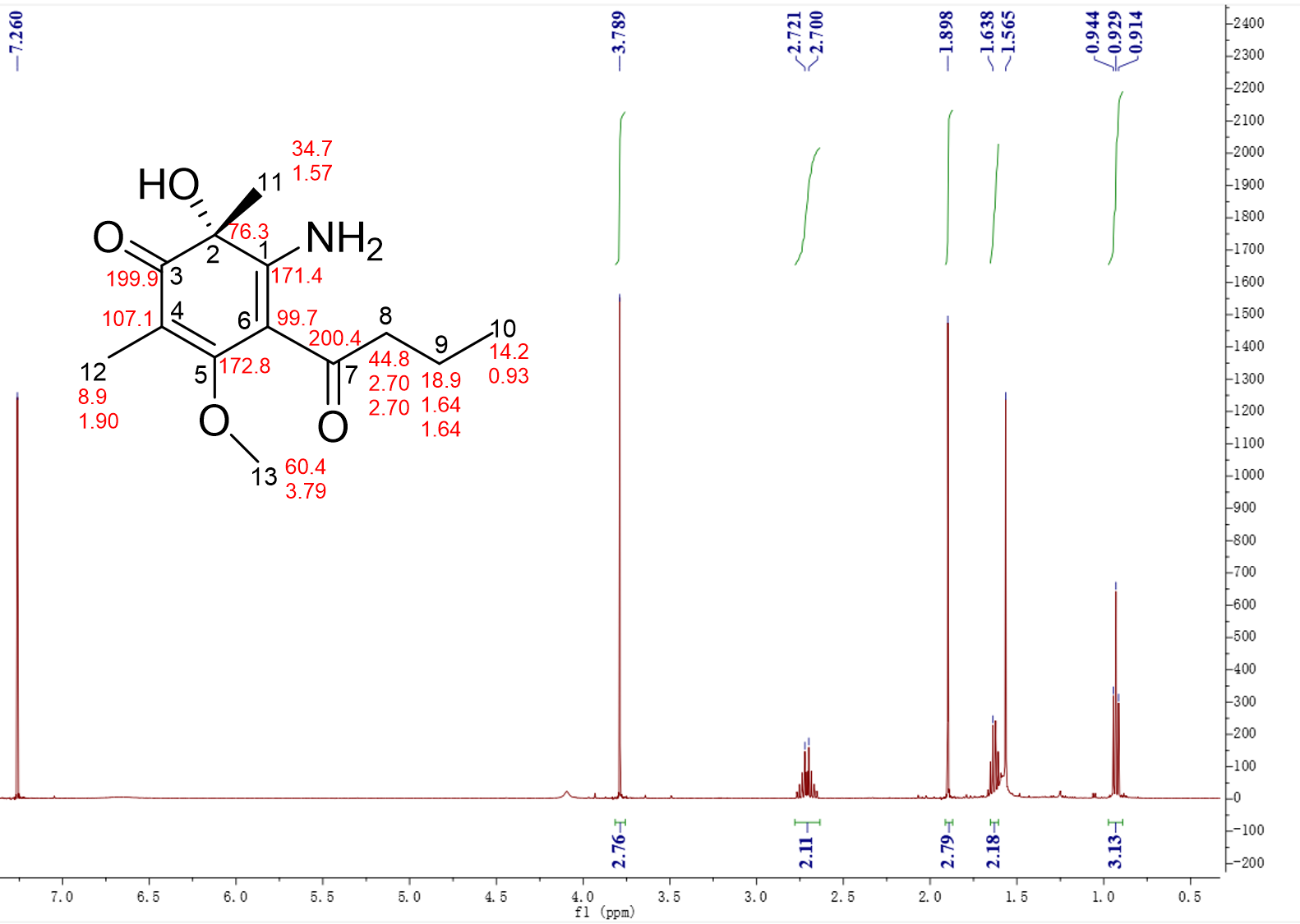


**Figure S21.** ^1^H NMR spectrum (500 MHz, CDCl_3_) of compound **2**


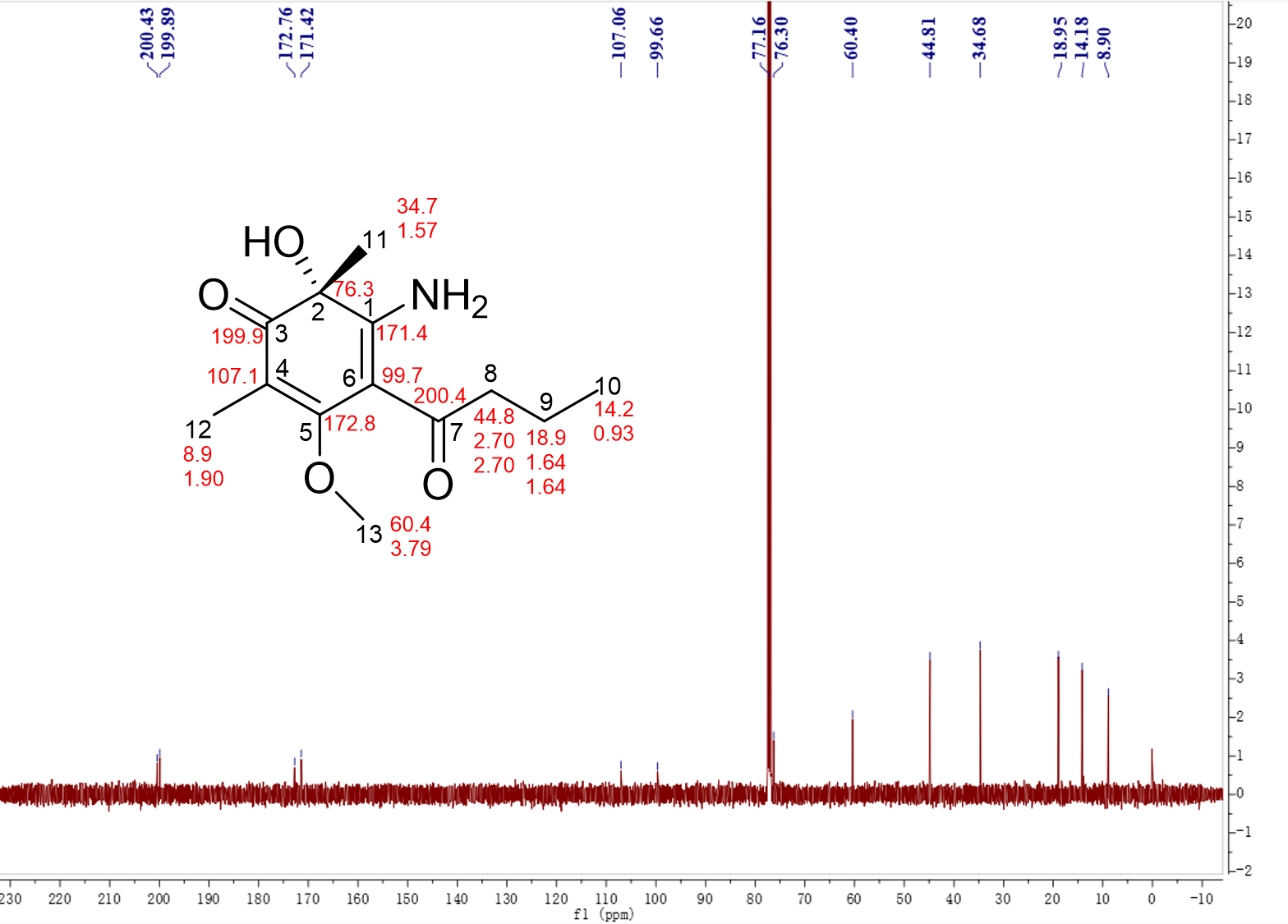


**Figure S22.** ^13^C NMR spectrum (125 MHz, CDCl_3_) of compound **2**


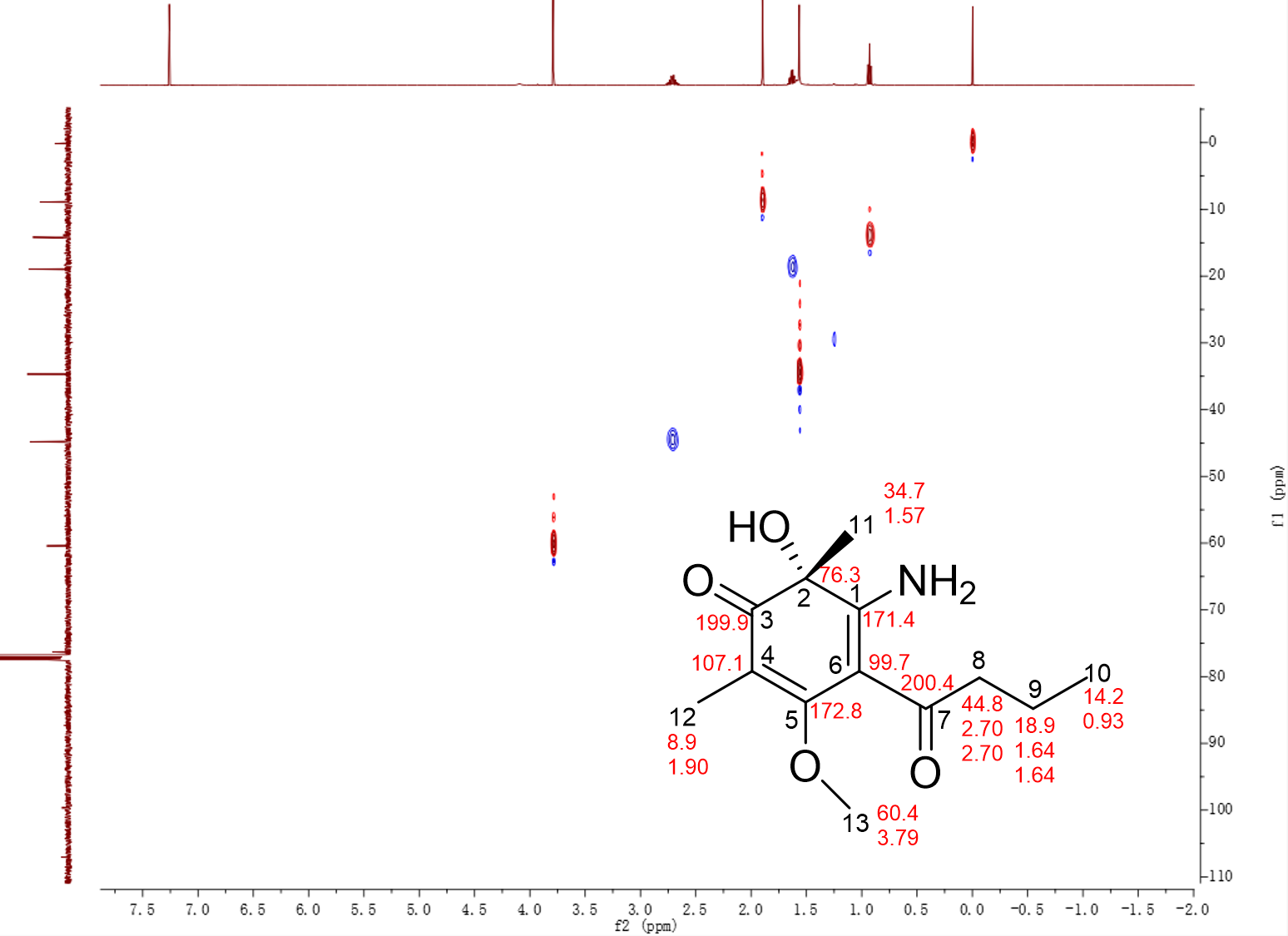


**Figure S23.** HSQC (500 MHz, CDCl_3_) of compound **2**


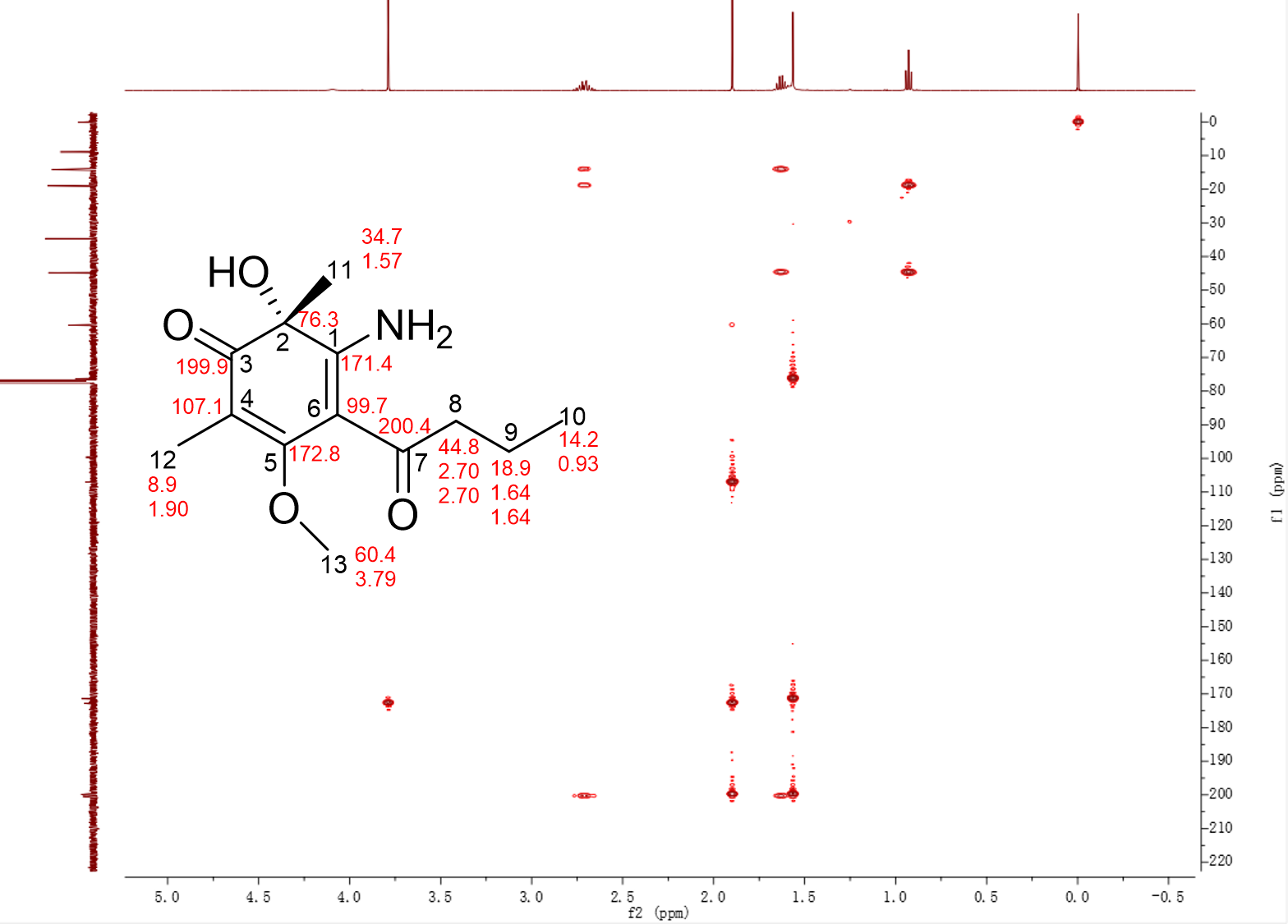


**Figure S24.** HMBC (125 MHz, CDCl_3_) of compound **2**


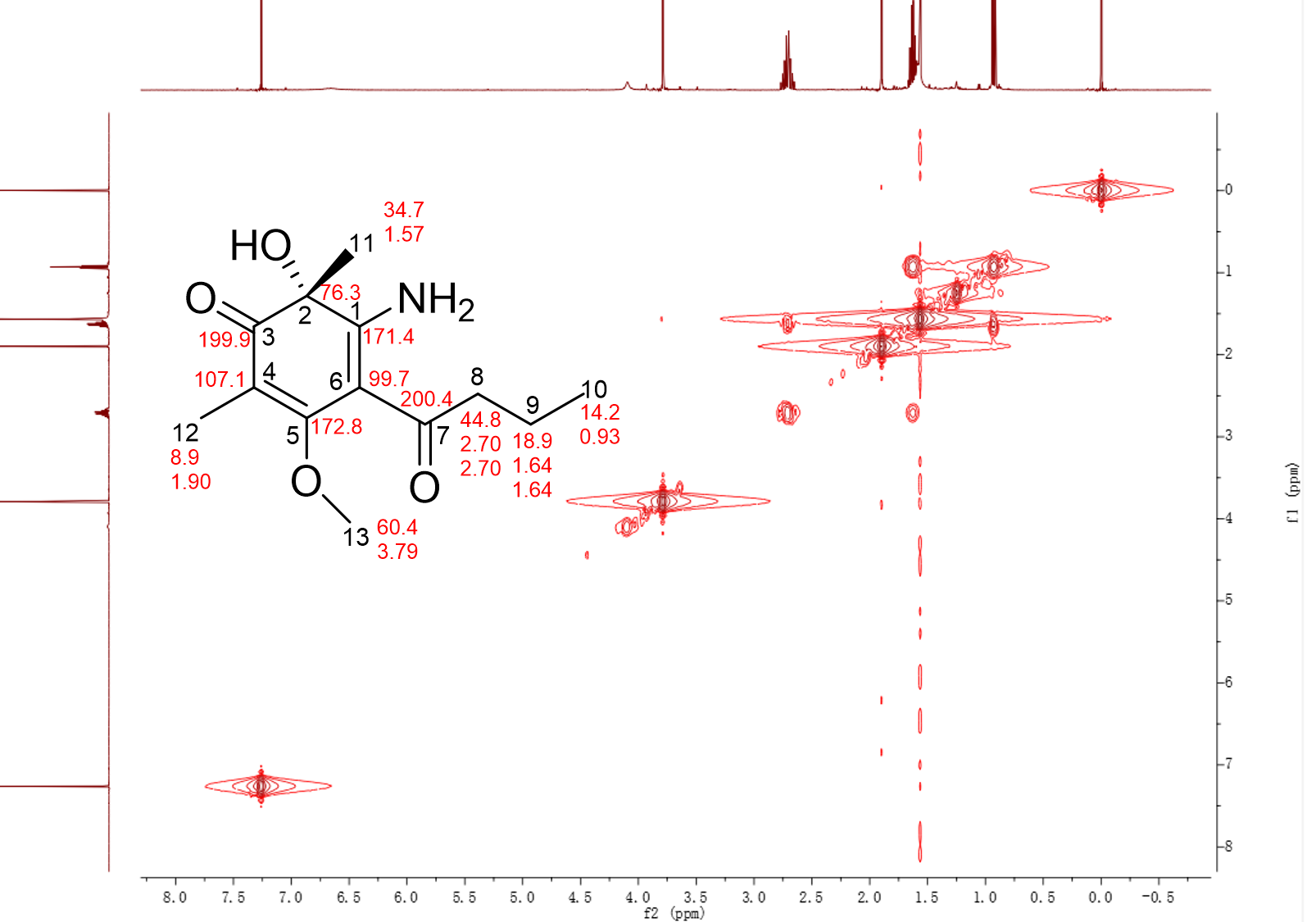


**Figure S25.** ^1^H-^1^H COSY (500 MHz, CDCl_3_) of compound **2**


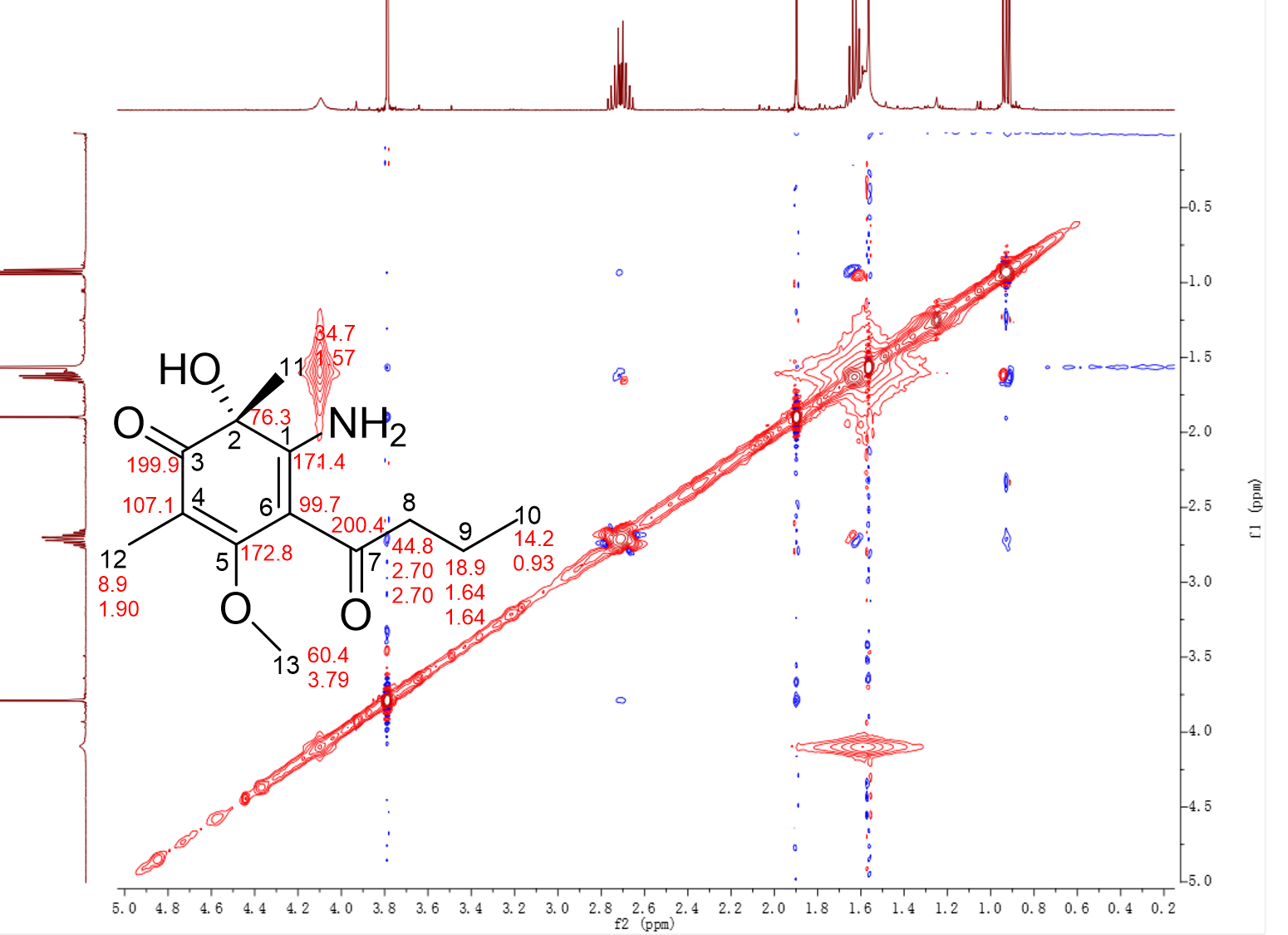


**Figure S26.** NOESY (500 MHz, CDCl_3_) of compound **2**

**Figure S27.** The positive HRESIMS spectrum of compound **3**


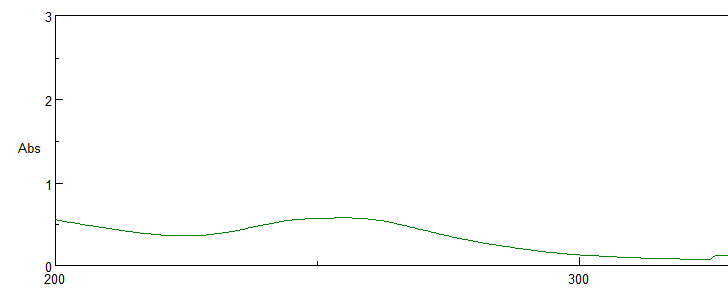


**Figure S28.** UV spectrum of compound **3**


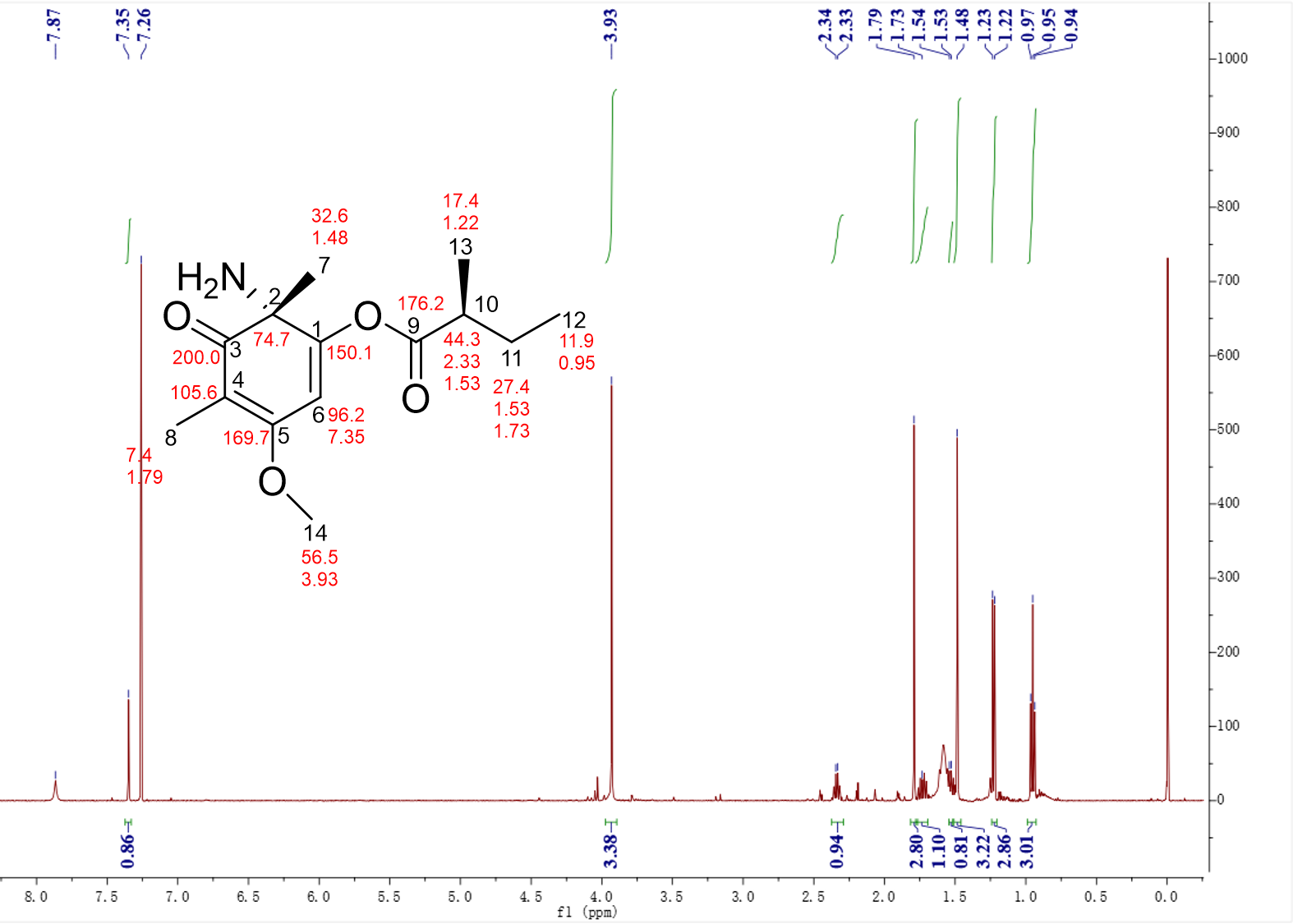


**Figure S29.** ^1^H NMR spectrum (500 MHz, CDCl_3_) of compound **3**

**
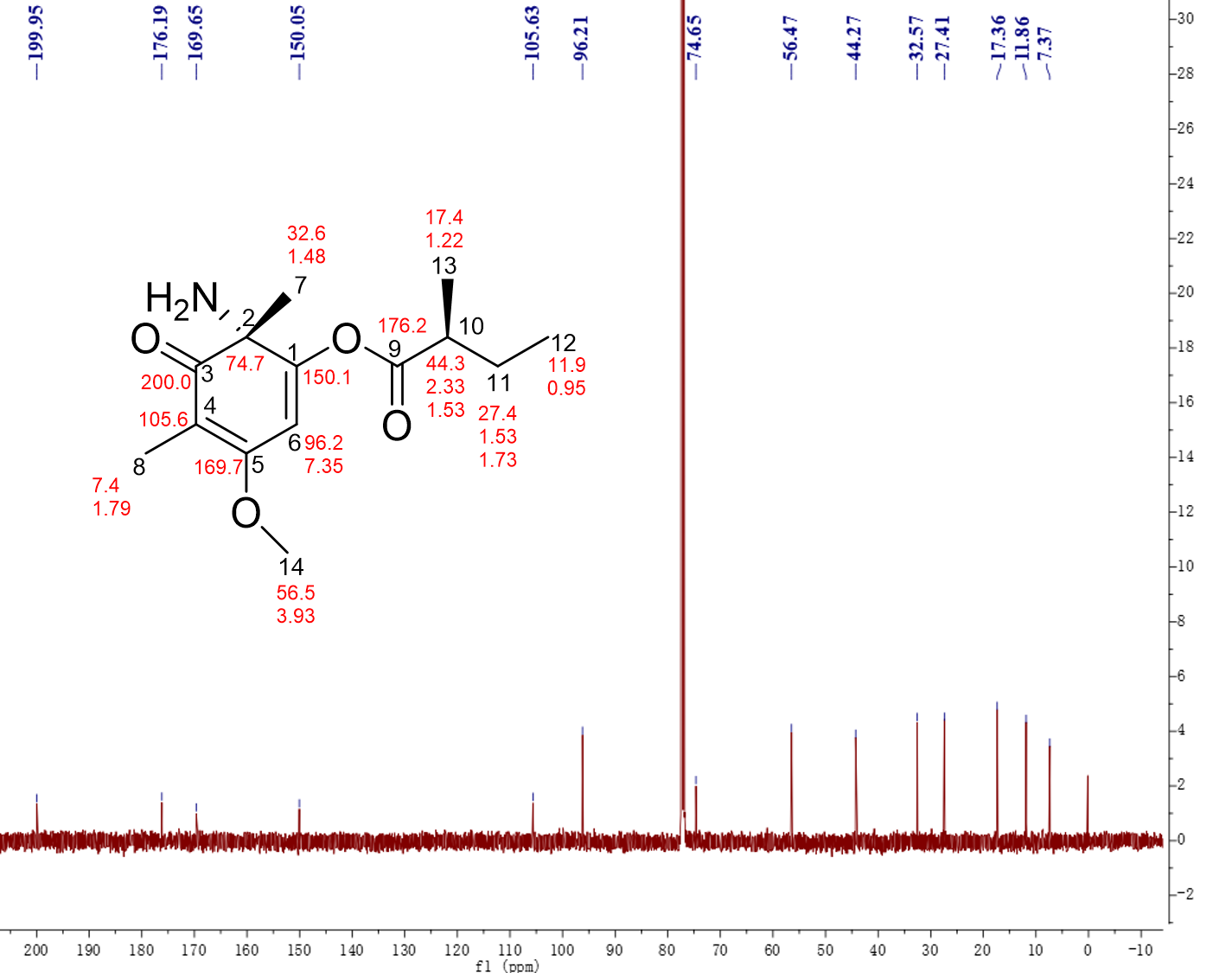
**

**Figure S30.** ^13^C NMR spectrum (125 MHz, CDCl_3_) of compound **3**

**
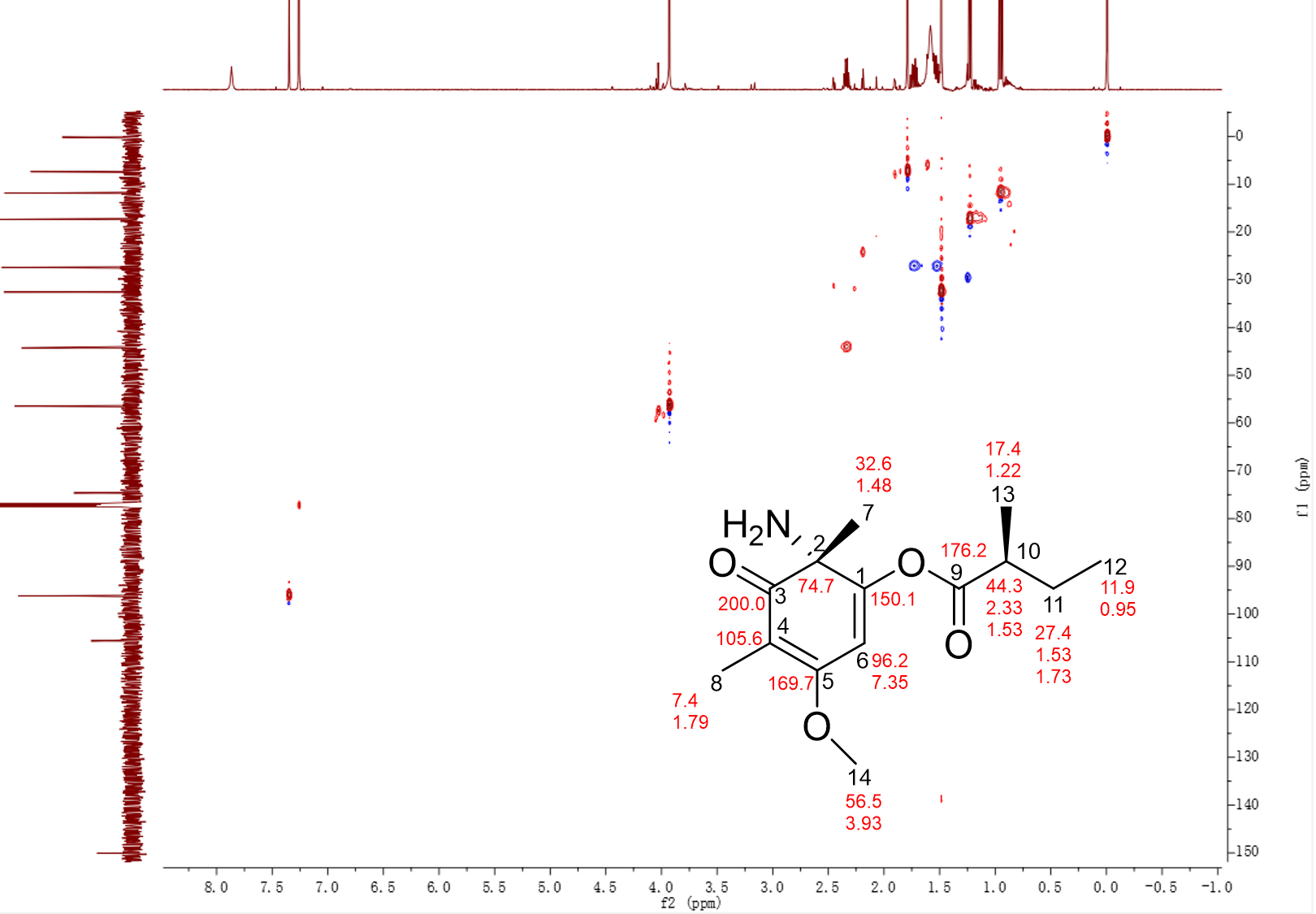
**

**Figure S31.** HSQC (500 MHz, CDCl_3_) of compound **3**


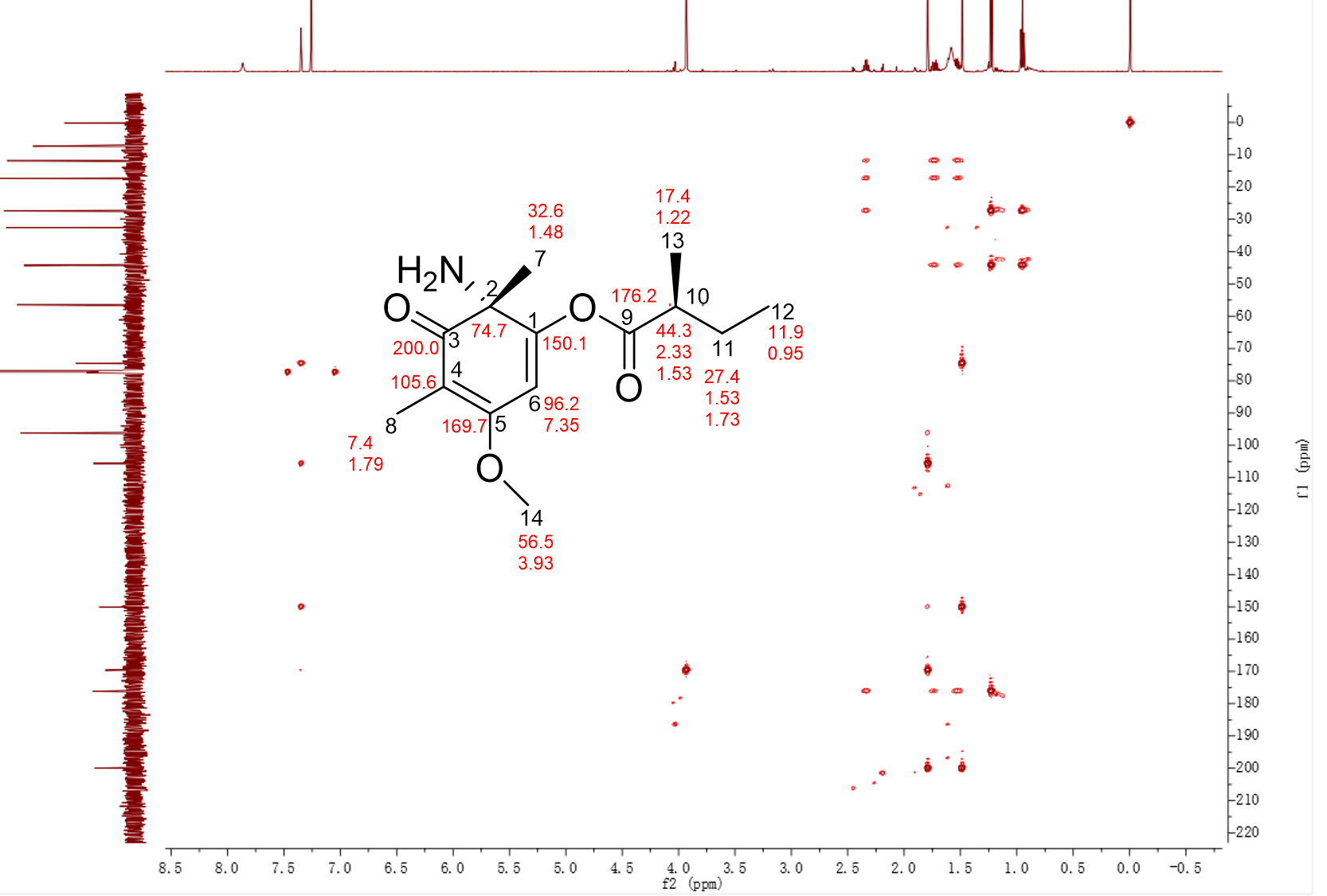


**Figure S32.** HMBC (125 MHz, CDCl_3_) of compound **3**


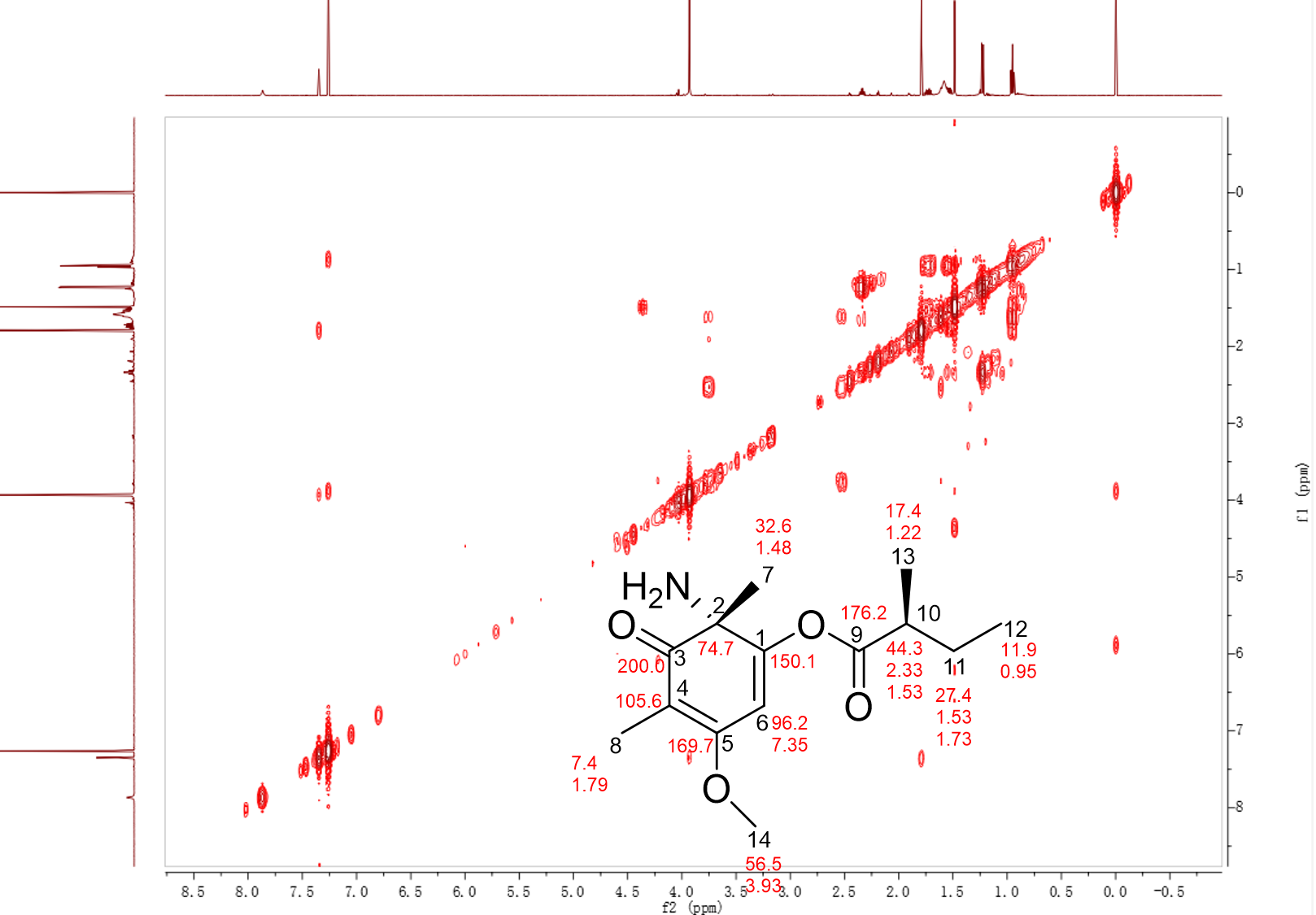


**Figure S33.** ^1^H-^1^H COSY (500 MHz, CDCl_3_) of compound **3**

**
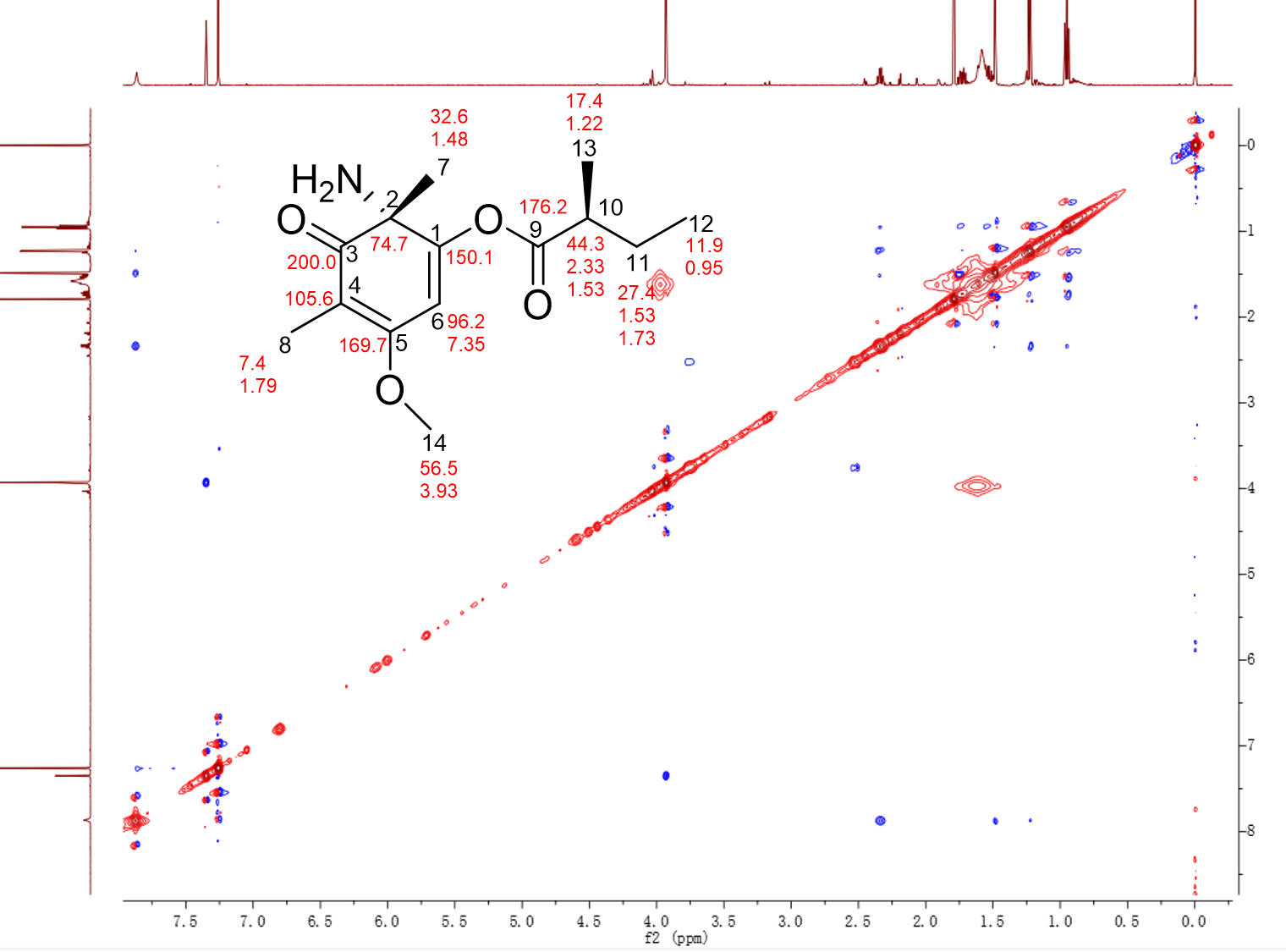
**

**Figure S34.** NOESY (500 MHz, CDCl_3_) of compound **3**

**Figure S35.** The positive HRESIMS spectrum of compound **4**


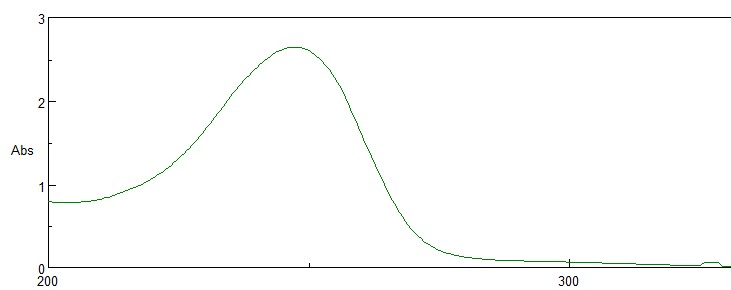


**Figure S36.** UV spectrum of compound **4**

**
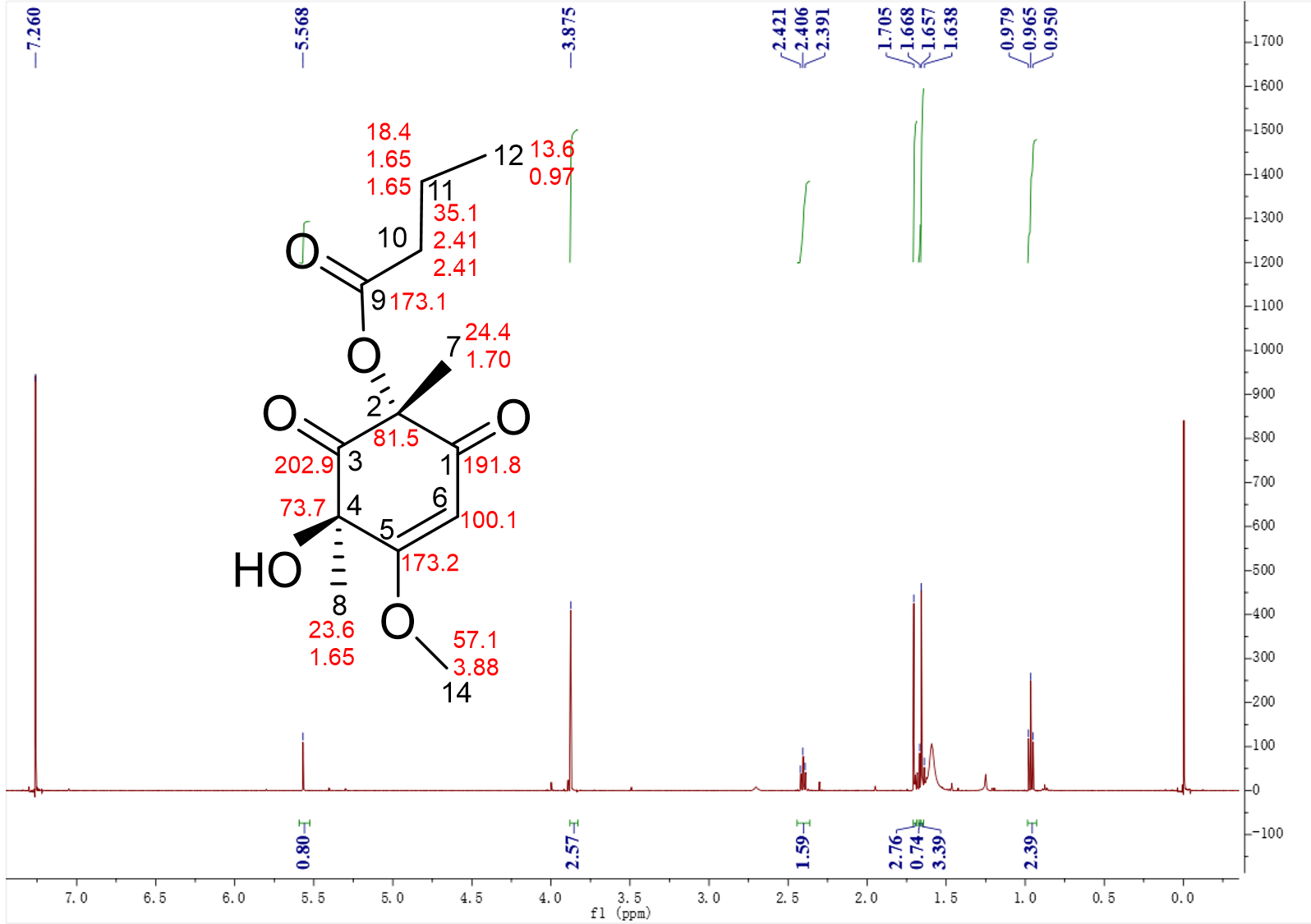
**

**Figure S37.** ^1^H NMR spectrum (500 MHz, CDCl_3_) of compound **4**

**
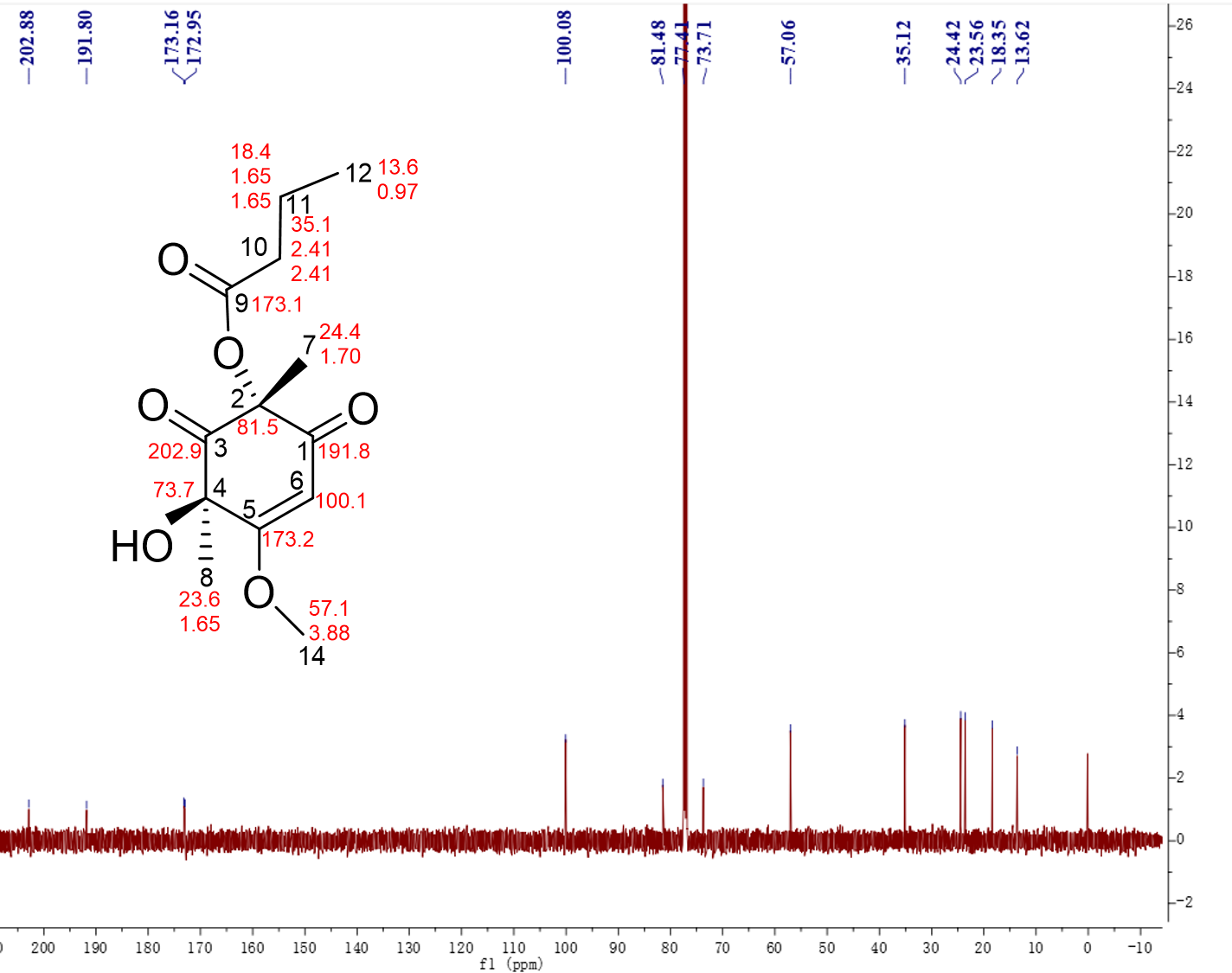
**

**Figure S38.** ^13^C NMR spectrum (125 MHz, CDCl_3_) of compound **4**

**
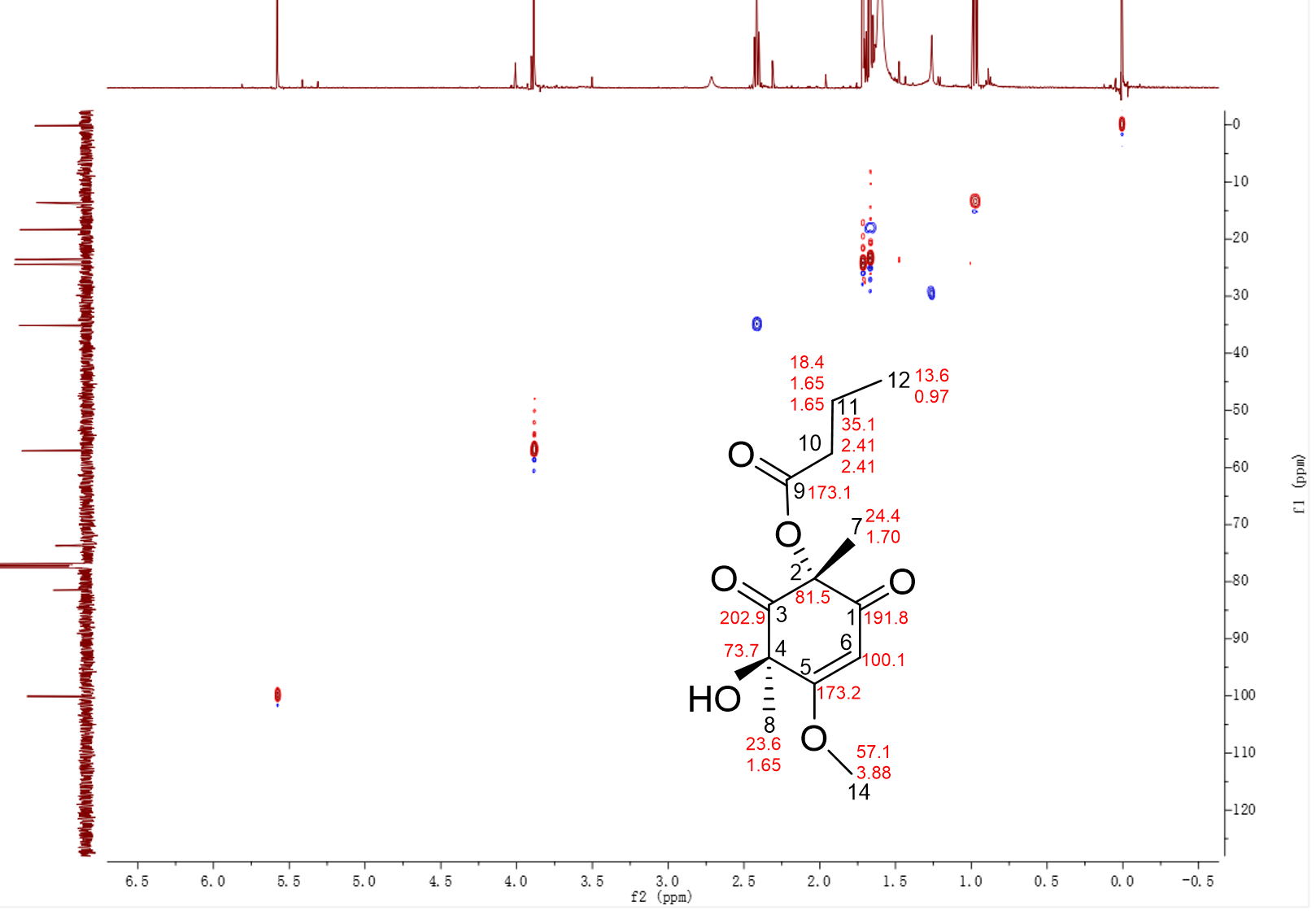
**

**Figure S39.** HSQC (500 MHz, CDCl_3_) of compound **4**

**
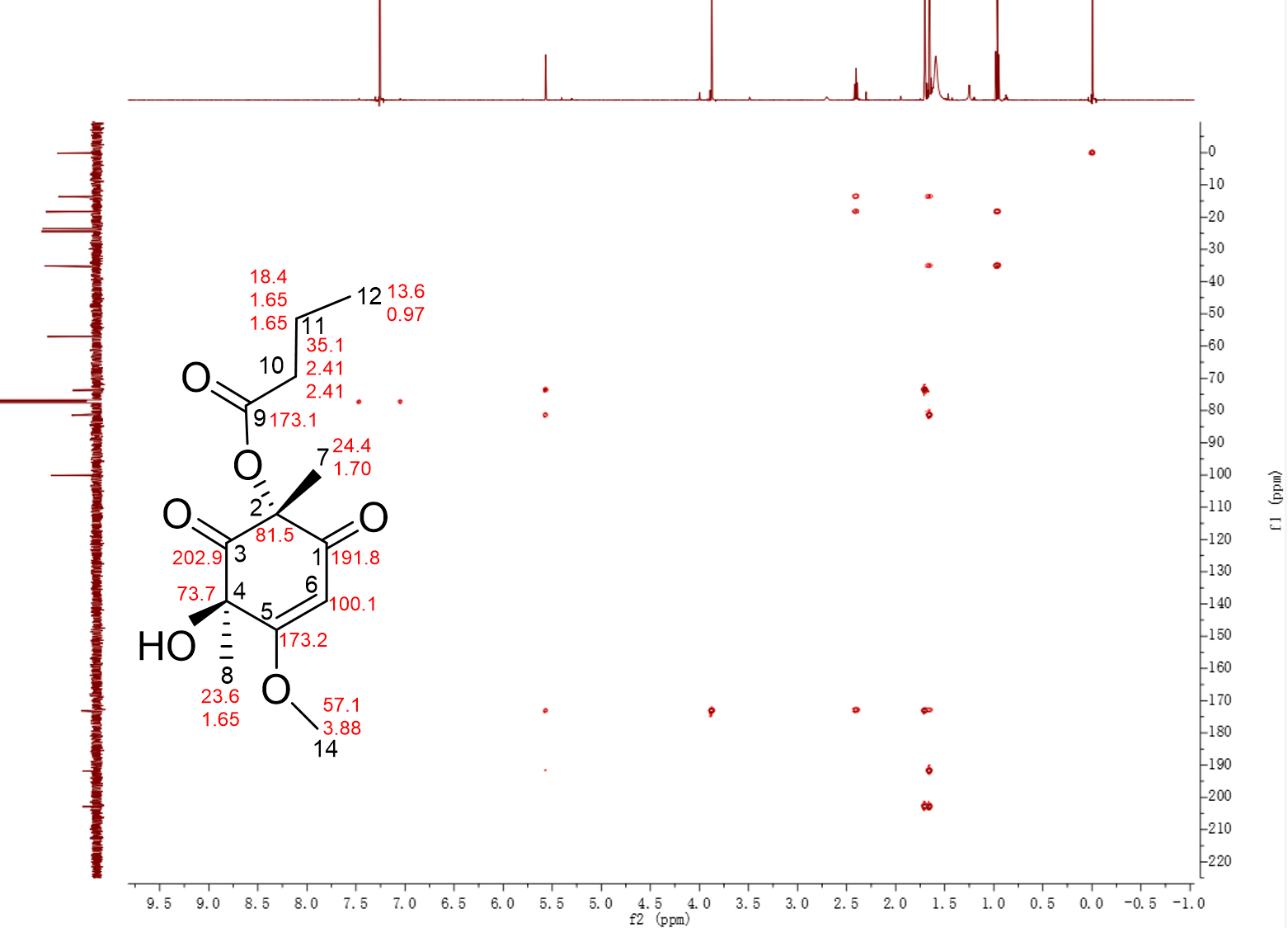
**

**Figure S40.** HMBC (125 MHz, CDCl_3_) of compound **4**

**
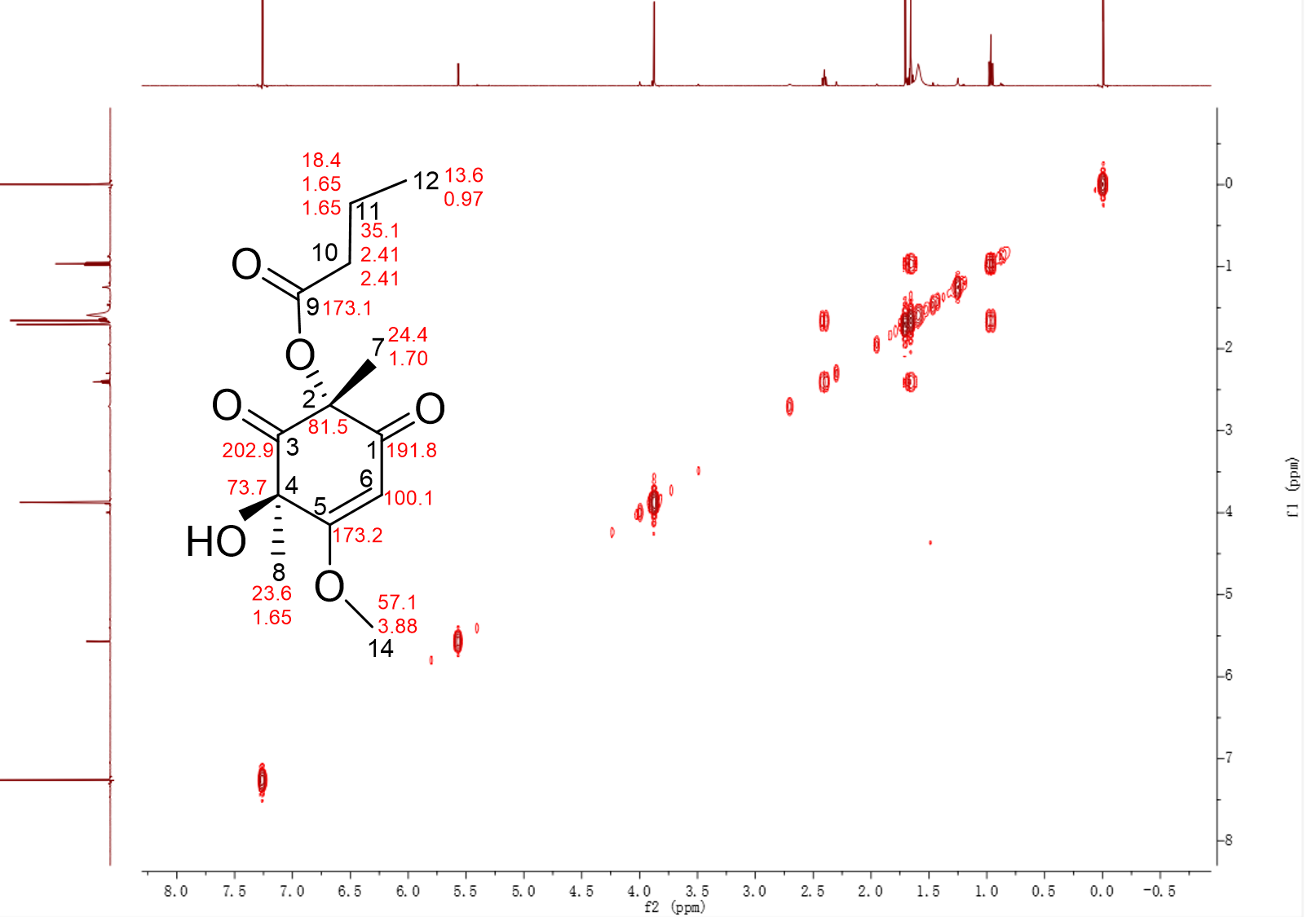
**

**Figure S41.** ^1^H-^1^H COSY (500 MHz, CDCl_3_) of compound **4**


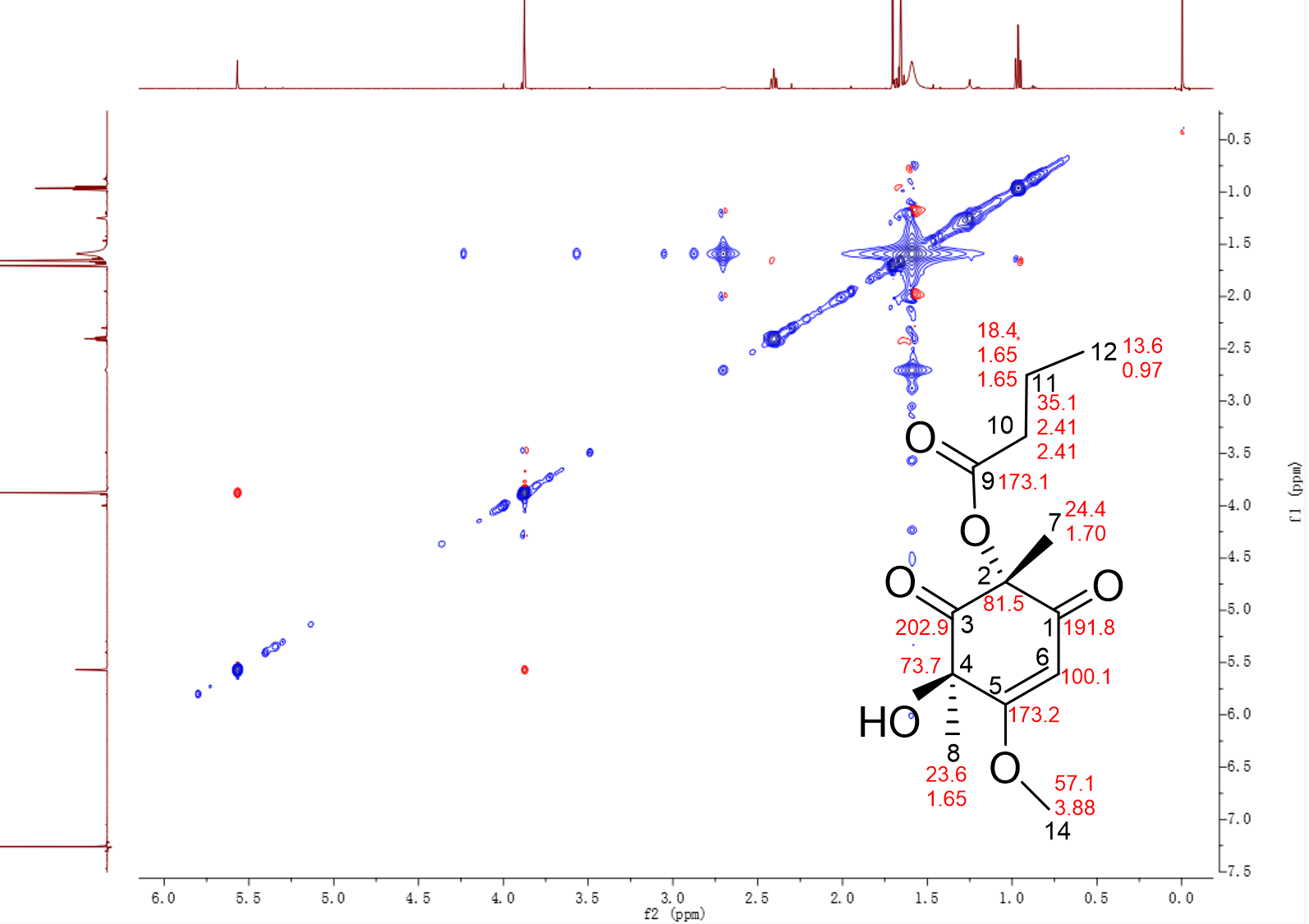


**Figure S42.** NOESY (500 MHz, CDCl_3_) of compound **4**

**Figure S43.** ^1^H NMR spectrum (500 MHz, CDCl_3_) of compound **5**

**Figure S44.** ^13^C NMR spectrum (125 MHz, CDCl_3_) of compound **5**

**Figure S45.** ^1^H NMR spectrum (500 MHz, CDCl_3_) of compound **6**

**Figure S46.** ^13^C NMR spectrum (125 MHz, CDCl_3_) of compound **6**

**Figure S47.** ^1^H NMR spectrum (500 MHz, CDCl_3_) of compound **7**

**Figure S48.** ^13^C NMR spectrum (125 MHz, CDCl_3_) of compound **7**
